# Tumor-infiltrating clonal hematopoiesis

**DOI:** 10.1056/NEJMoa2413361

**Published:** 2025-04-24

**Authors:** Oriol Pich, Elsa Bernard, Maria Zagorulya, Andrew Rowan, Constandina Pospori, Ramy Slama, Hector Huerga Encabo, Jennifer O’Sullivan, Despoina Papazoglou, Panayiotis Anastasiou, Chrysante S. Iliakis, Sally-Ann Clark, Krijn K. Dijkstra, Vittorio Barbè, Chris Bailey, Aaron J. Stonestrom, Katey S.S. Enfield, Mary Green, Charlotte K. Brierley, Alastair Magness, David R. Pearce, Robert E. Hynds, Rija Zaidi, Jayant K. Rane, Ángel F. Álvarez-Prado, Kerstin Thol, Rachel Scott, Supreet Kaur. Bola, Elena Hoxha, Steve K. Harris, Karl S. Peggs, Sergio A. Quezada, Allan Hackshaw, Simone Zaccaria, Johanna A. Joyce, Ilaria Malanchi, Michael F. Berger, Mariam Jamal-Hanjani, Andreas Wack, Julian Downward, William Grey, Cristina Lo Celso, Eva Gronroos, Charles M. Rudin, Adam J. Mead, Dominique Bonnet, Elli Papaemmanuil, Charles Swanton

**Affiliations:** 1 Cancer Evolution and Genome Instability Laboratory, The Francis Crick Institute, London, UK; 2 Computational Clinical Oncology Laboratory, UMR 981, Gustave Roussy, Villejuif, France; 3 Bone Marrow Dynamics, The Francis Crick Institute, London, UK; 4 Imperial College London, London, UK; 5 Haematopoietic Stem Cell Biology Laboratory, Medical Research Council Weatherall Institute of Molecular Medicine, University of Oxford, Oxford; 6 Haematopoietic Stem Cell Laboratory, The Francis Crick Institute, London, UK; 7 Oncogene Biology Laboratory, The Francis Crick Institute, London UK; 8 Immunoregulation Laboratory, The Francis Crick Institute, London, UK; 9 Flow Cytometry Facility, Medical Research Council Weatherall Institute of Molecular Medicine, University of Oxford, Oxford; 10 Department of Molecular Oncology and Immunology, the Netherlands Cancer Institute, 1066 CX Amsterdam, the Netherlands; 11 Oncode Institute, Utrecht, the Netherlands; 12 University College London Hospitals NHS Foundation Trust, London, UK; 13 Greenebaum Comprehensive Cancer Center, University of Maryland School of Medicine, Baltimore, USA; 14 Experimental Histopathology, The Francis Crick Institute, London, UK; 15 Oxford University Hospitals NHS Foundation Trust, Oxford, UK; 16 University College London Cancer Institute, London, UK; 17 Computational Cancer Genomics Research Group, University College London Cancer Institute, London, UK; 18 Department of Oncology, University of Lausanne, Lausanne 1011, Switzerland; 19 Ludwig Institute for Cancer Research, University of Lausanne 1011, Lausanne, Switzerland; 20 Agora Cancer Research Centre Lausanne, Lausanne 1011, Switzerland; 21 L. Lundin and Family Brain Tumor Research Center, Departments of Oncology and Clinical Neurosciences, Centre Hospitalier Universitaire Vaudois, Lausanne 1011, Switzerland; 22 Cancer Genome Evolution Research Group, Cancer Research UK Lung Cancer Centre of Excellence, University College London Cancer Institute, London, UK; 23 Cancer Research UK Lung Cancer Centre of Excellence, University College London Cancer Institute, London, UK; 24 Cancer Metastasis Laboratory, University College London Cancer Institute, London, UK; 25 Cancer Immunology Unit, Immune Regulation and Tumour Immunotherapy Group, Research Department of Haematology, University College London Cancer Institute, London, UK; 26 University College London Hospitals Biomedical Research Centre, London, UK; 27 Institute of Health Informatics, University College London, London, UK; 28 Cancer Research UK & UCL Cancer Trials Centre, London, UK; 29 Computational Cancer Genomics Research Group, University College London Cancer Institute, London, UK; 30 Tumour-Host Interaction Laboratory, The Francis Crick Institute, London, UK; 31 Department of Pathology and Laboratory Medicine, Memorial Sloan Kettering Cancer Center, New York, NY 10065, USA; 32 Cancer Metastasis Laboratory, University College London Cancer Institute, London, UK; 33 Department of Medical Oncology, University College London Hospitals, London, UK; 34 Proteostem laboratory, Centre for Blood Research, York Biomedical Research Institute, Department of Biology, University of York, UK; 35 Department of Medicine, Memorial Sloan Kettering Cancer Center, New York, NY, USA; 36 Computational Oncology Service, Department of Epidemiology and Biostatistics, Memorial Sloan Kettering Cancer Center, New York, NY, USA

## Abstract

**Background.:**

Clonal hematopoiesis of indeterminate potential (CHIP) is an age-related condition associated with increased mortality in patients with cancer. CHIP mutations with high variant allele frequencies can be detected in tumors, a phenomenon we term tumor-infiltrating clonal hematopoiesis (TI-CH). The frequency of TI-CH and its impact on tumor evolution is unclear.

**Methods.:**

We characterized CHIP and TI-CH in 421 patients with early-stage non-small cell lung cancer (NSCLC) from the TRACERx study and 49,351 patients from the MSK-IMPACT pan-cancer cohort. We studied the association of TI-CH with survival and disease recurrence and evaluated the functional impact of *TET2*-mutant-CHIP on lung tumor biology.

**Results.:**

In NSCLC, 42% of patients with CHIP had TI-CH. TI-CH independently predicted the risk of death or recurrence, with an adjusted hazard ratio of 1.80 [95% CI 1.23-2.66] compared to the absence of CHIP and of 1.62 [95% CI 1.02-2.56] compared to CHIP in absence of TI-CH. In solid tumors, 26% of patients with CHIP had TI-CH. TI-CH conferred a 1.17 [95% CI 1.06-1.29] greater risk of all-cause mortality compared to CHIP in absence of TI-CH. *TET2* mutations were the strongest genetic predictor of TI-CH, enhanced monocyte migration to lung tumor cells, fuelled a myeloid-rich tumor microenvironment in mice, and resulted in the promotion of tumor organoid growth.

**Conclusion.:**

TI-CH increases the risk of death or recurrence in NSCLC and of all-cause mortality in solid tumors. TI-CH remodels the tumor immune microenvironment and accelerates tumor organoid growth, supporting a role for an aging-related hematologic clonal proliferation in cancer evolution.

## INTRODUCTION

Clonal hematopoiesis of indeterminate potential (CHIP) is a prevalent age-associated condition involving the expansion of blood cells derived from a somatically mutated hematopoietic stem cell without hematologic disorders.^1,2^ CHIP increases the risk of hematological malignancies,^3–6^ and chronic inflammatory diseases, such as cardiovascular disease, chronic obstructive pulmonary disease, and chronic liver disease.^7–11^ CHIP is also associated with increased incidence of lung cancer,^12,13^ and risk of death in solid tumors.^14,15^

CHIP-derived immune cells have been detected in solid tumor tissues from patients with CHIP,^16–18^ with an enrichment in non-small cell lung cancer (NSCLC),^18^ where they have the potential to alter the local microenvironment and influence tumor evolution.^19,20^ While more than 20% of patients with cancer have CHIP,^14,15^ the impact of tumor-infiltrating immune cells with CHIP mutations on tumor progression and patient outcomes remains poorly understood.

Here, we define the presence of CHIP mutations with high variant allele frequencies within tumors as tumor-infiltrating clonal hematopoiesis (TI-CH). We determine the prevalence of TI-CH in 421 patients with early-stage NSCLC and 49,351 patients across 75 cancer types from a real-world cohort, study its associations with patient outcomes, and evaluate its functional impact using preclinical lung cancer models.

## METHODS

### Study cohorts

We studied 421 patients with treatment-naïve stage IA-IIIA NSCLC from the TRACERx study (NCT01888601) (Table 1 and Fig S1), including metastases sampled at autopsy from two patients also enrolled in the CRUK autopsy PEACE program (NCT03004755).^21,22^ Patients were subject to post-surgical multi-region tumor sampling and to blood collection before surgery. A total of 1,560 tumor regions were analyzed (median 3 regions per patient, range 2-10). We included a pan-cancer MSK-IMPACT cohort of 49,351 patients across 75 cancer types comprising primary (n=31,556) and metastatic (n=17,795) tumors with matched blood.^23,24^ Findings from TRACERx were validated in 2,602 patients with stage I-III NSCLC from MSK-IMPACT (Table 1).

### Genomic and immunophenotyping analyses

CHIP was detected in blood using a 2% variant allele frequency (VAF) cutoff across 77 myeloid driver genes (Table S1), as per prior studies.^5,15,25–27^ CHIP mutations were genotyped in the matched tumor samples. Spatial immunophenotyping was performed using multiplex imaging mass cytometry for 163 tumor samples from 70 TRACERx patients.^28^ Single-cell mutational analysis of immune cells was performed for two TRACERx patients harboring TI-CH.^29^

### Functional studies of lung adenocarcinoma and *TET2*-mutant CHIP

Tet2-mutant CHIP mice were generated by transplanting 1:1 mix of congenically-marked Tet2-mutant and wild-type bone marrow cells into busulfan-conditioned wild-type recipients.^30^ Lung tumors were induced via orthotopic transplantation of 3LL lung adenocarcinoma (LUAD) cells.^31^ Tumor, adjacent lungs and blood were analyzed by flow cytometry. For the organoid experiment, TRACERx patient-derived lung tumor organoid cells were cultured with human myeloid cells isolated from lungs of immunodeficient mice engrafted with human *TET2*-mutant or wild-type hematopoietic stem and progenitor cells.^32^ Detailed methods are available in the Supplementary Appendix.

### Statistical Analysis

CHIP and TI-CH presence was associated with clinical parameters, tumor genomic alterations, tumor microenvironment composition, and patient outcomes. Time-to-event analysis was performed using Cox proportional hazards regression controlled for patient age, sex, ethnicity, smoking status, treatment, tumor stage, and histology. Adjustments for multiplicity were not made to 95% confidence intervals (CI), where relevant. When examining the association between multiple cancer types and CHIP or TI-CH, the Benjamini-Hochberg correction for multiple testing was applied.

## RESULTS

### CHIP in early-stage NSCLC

The associations between CHIP and risk of mortality have not been evaluated in early-stage treatment-naïve NSCLC or with lung-cancer specific endpoints. In TRACERx, CHIP mutations were observed in 34% (143/421) of patients with early-stage treatment-naïve NSCLC (Table 1 and Fig S1), with a 5% median VAF (interquartile range [IQR] 2.9-11%), most commonly affecting *DNMT3A*, *TET2*, and *ASXL1*. Age was the only baseline variable associated with CHIP in multivariable analysis (Fig S1). CHIP was equally prevalent in LUAD and lung squamous carcinoma (LUSC) (Fig S2), and NSCLC genetic drivers were similar between CHIP-positive and negative patients (Table 1 and Fig S3).^21,22^

**Figure 1.**
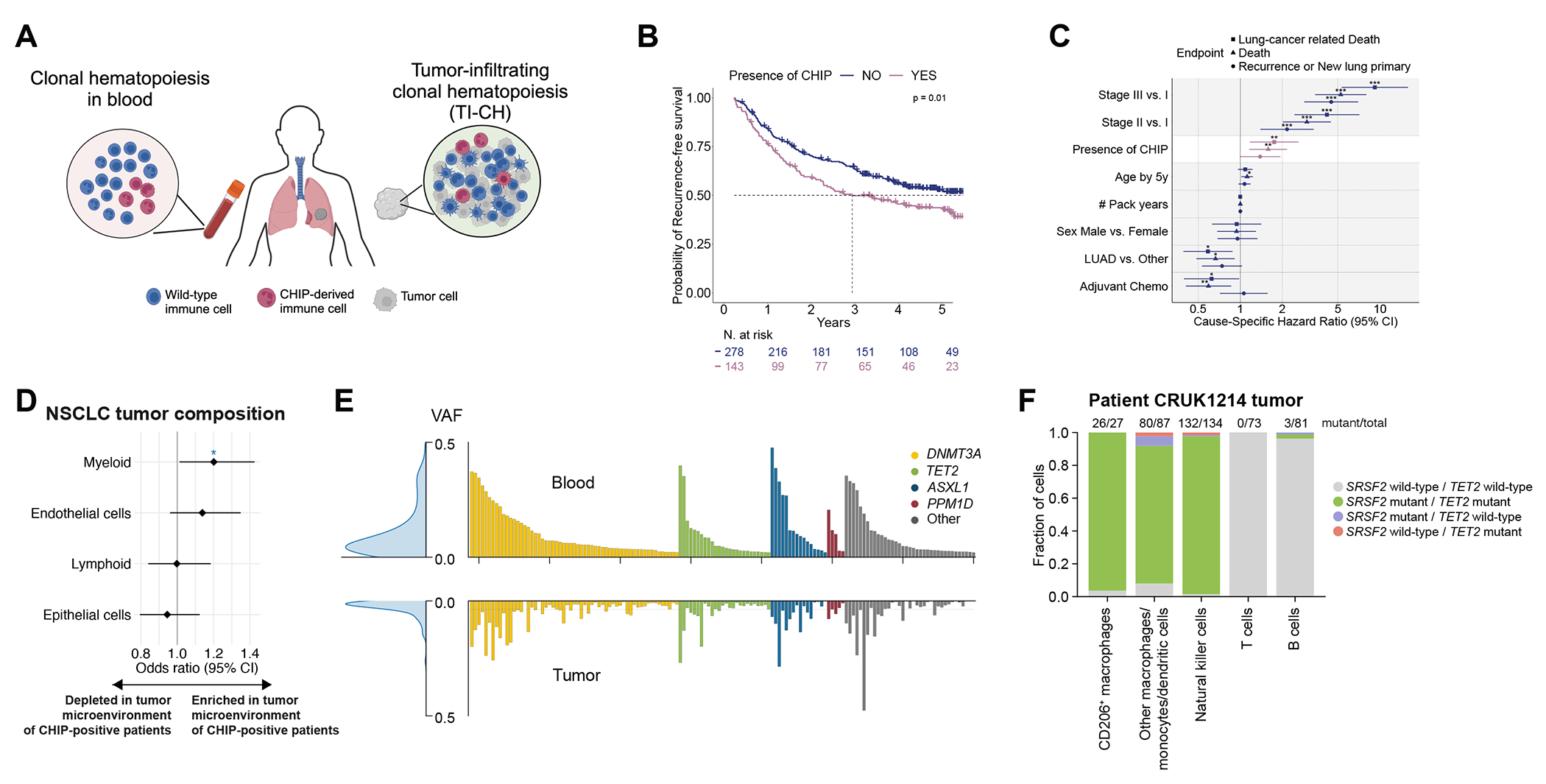
CHIP and TI-CH in patients with NSCLC enrolled in TRACERx. **A.** Schematic representation of CHIP and TI-CH in a patient with lung cancer. **B.** Kaplan-Meier probability estimates of recurrence-free survival across patients without (blue) or with (purple) CHIP in TRACERx. P-value is from the log-rank test. **C.** Cause-specific multivariable Cox models across three clinical endpoints, the risk of lung-cancer related death, the risk of all-cause mortality, and the risk of recurrence or new primary lung cancer. Age in years was used as a continuous variable, and divided by 5 so that the hazard ratio represents the change in risk for a 5-year increase. CI: confidence interval; LUAD: lung adenocarcinoma. *p<0.05, **p<0.01,***p<0.001. **D.** Age-adjusted logistic regression analysis for the association between the presence of CHIP and cellular densities (higher vs. lower than the cohort average for each cell type) in the lung tumor microenvironment. CI: confidence interval. *p<0.05. **E.** Barplot representing the variant allele frequency (VAF) of CHIP mutations in the blood (top) and tumor (bottom) samples for 143 patients with CHIP. Each column corresponds to a patient. The bars are colored according to the mutated gene. For cases with more than one CHIP mutation, the highest VAF was selected. Density distributions of VAF values in blood (top) and tumor (bottom) samples are depicted on the left. **F.** Single-cell genotyping of different immune cell populations isolated from a primary lung tumor of a patient with *TET2*- and *SRSF2*-mutant TI-CH. Cells are color coded according to their genotypes within each cell population.

Patients with CHIP had shorter recurrence-free and overall survival compared to patients without CHIP (hazard ratio [HR]=1.42, 95% CI 1.07-1.88 for recurrence-free survival, and HR=1.59, 95% CI 1.18-2.15 for overall survival). The median recurrence-free survival was 2.7 (95% CI 2.0-5.0) and 6.0 (95% CI 3.8-not reached) years in the presence and absence of CHIP, respectively (Fig 1B). For overall survival, it was 4.0 (95% CI 2.8-not reached) and 6.0 (95% CI 5.5-not reached) years, respectively (Fig S4).

We assessed the association in multivariable analysis between CHIP and patient outcomes for the risk of lung-cancer related death, all-cause mortality, and tumor recurrence or new primary lung cancer. The adjusted hazard ratios were 1.75 (95% CI 1.17-2.62), 1.59 (95% CI 1.16-2.17), and 1.39 (95% CI 0.99-1.94), respectively (Fig 1C and Fig S5).

### Associations between CHIP and lung tumor microenvironment

The associations between CHIP and lung-cancer specific outcomes prompted us to evaluate its relationship with the tumor microenvironment. The 46 primary tumor regions from 21 patients with CHIP had a higher representation of myeloid cells compared to the 117 tumor regions from 49 patients without CHIP (Fig 1D and Fig S6; age-adjusted odds ratio [OR]=1.20, 95% CI 1.01-1.43). Among myeloid cell subtypes, monocytes (odds ratio [OR]=1.37, 95% CI 1.18-1.59) and neutrophils (OR=1.31, 95% CI 1.14-1.51) were specifically enriched (Fig S6). There was no difference in the abundance of T and B cells.

### Tumor-infiltrating clonal hematopoiesis in NSCLC

The enrichment of myeloid cells within primary lung tumors of CHIP-positive patients suggested these cells may originate from CHIP lineages. Thus, for each patient with CHIP, we genotyped the corresponding CHIP mutation(s) in the matched lung tumor samples (179 mutations across 143 patients with CHIP, median 1 mutation per patient, range 1-4) (Fig S1 and Fig S7).

In 96% (137/143) of patients with CHIP, the corresponding CHIP mutation was detected in tumors. The VAF of CHIP mutations in tumors had a median of 1.6% (IQR 0.88-3.4%) (Fig 1E), positively correlated with the VAF in blood (Pearson’s r = 0.67, p<0.001, Fig S8), and was consistently lower than the VAF in blood.

To identify tumors with robust infiltration of CHIP-derived immune cells, we defined tumor-infiltrating clonal hematopoiesis, or TI-CH, as the presence of CHIP mutations within tumors at a VAF of 2% or more in at least one region. Among the 143 patients with CHIP, 42% (60/143) had TI-CH (Fig 1E). In LUAD and LUSC, TI-CH was observed in 34% (27/80) and 50% (25/50) of patients with CHIP, respectively. TI-CH was equally prevalent in patients with stage I (42%, 32/77), II (41%, 18/44), or III (45%, 10/22) disease. We assessed TI-CH intratumor heterogeneity by examining 226 tumor regions from the 60 TRACERx patients with TI-CH (median 4 regions per patient, range 2-8) (Fig S9). Among these patients, 83% had TI-CH in at least half of the tumor regions. In 4 patients with synchronous or metachronous primary lung tumors, the TI-CH clone identified in one primary tumor was also detected in the other primary tumor, and 84% (16/19) of tumor regions qualified for TI-CH (Fig S10).

We examined the prevalence of TI-CH in primary tumors from a validation cohort of 2,602 patients with stage I-III NSCLC (Table 1). CHIP mutations were detected in 35% (917/2,602) of patients, with 6.0% (IQR 3.2-13%) median VAF in blood and 1.3% (IQR 0.56-3.0%) in tumors. Among patients with CHIP, 36% (333/917) had TI-CH, a similar proportion to TRACERx. In LUAD and LUSC, TI-CH was observed in 35% (237/685) and 45% (59/132) of patients with CHIP, respectively. The higher prevalence of TI-CH in LUSC compared to LUAD (OR=1.53 95% CI 1.03-2.27, p=0.03) may reflect histology-specific differences in the composition of the tumor immune microenvironment.^28^ In patients with low or intermediate/high clonal hematopoiesis risk score,^6^ TI-CH was observed in 32% (128/405) and 45% (70/156) of cases, respectively. Patients with *TET2*-mutant CHIP had the highest frequency of TI-CH (44%, 63/144), followed by patients with *ASXL1*-mutant CHIP (41%, 20/49), *DNMT3A*-mutant CHIP (34%, 161/467), and *PPM1D*-mutant CHIP (24%, 14/59) (Fig S11).

To assess which immune cells harbored CHIP mutations in TI-CH, we performed single-cell genotyping^29^ on the tumor immune infiltrate from two TRACERx patients with TI-CH that were not part of the discovery cohort (Fig 1F and Fig S12A–B): CRUK1214, harboring mutations in *TET2* (VAF of 43% in blood and 26% in tumor) and *SRSF2* (VAF of 43% in blood and 20% in tumor), and CRUK1358, with a single *TET2* mutation (VAF of 12% in blood and up to 3% in tumor). Despite the high VAFs of CHIP mutations, CRUK1214 did not exhibit monocytosis (Table S2). In both patients, the CHIP mutations were enriched across myeloid populations, including CD206^+^ macrophages, other mononuclear phagocytes (macrophages, monocytes, dendritic cells), as well as natural killer cells, present in smaller fractions of B cells, and practically absent in T cells (Fig 1F and S12C). We further validated the enrichment of CHIP mutations in tumor-infiltrating myeloid cells using published data from blood and metastatic brain tumor samples from a NSCLC patient with *TET2*-mutant CHIP (Fig S12D–E).^33^

### Association between tumor-infiltrating clonal hematopoiesis and patient outcomes in NSCLC

We assessed the associations between TI-CH, recurrence-free and overall survival in TRACERx. We defined three categories of patients: (i) without CHIP; (ii) with CHIP in absence of TI-CH (i.e. blood-only CHIP); and (iii) with TI-CH (Table S3). There was no clinically relevant difference in age of patients with blood-only CHIP compared to TI-CH (median age 71 vs. 74 years, which was not significant).

**Figure 2.**
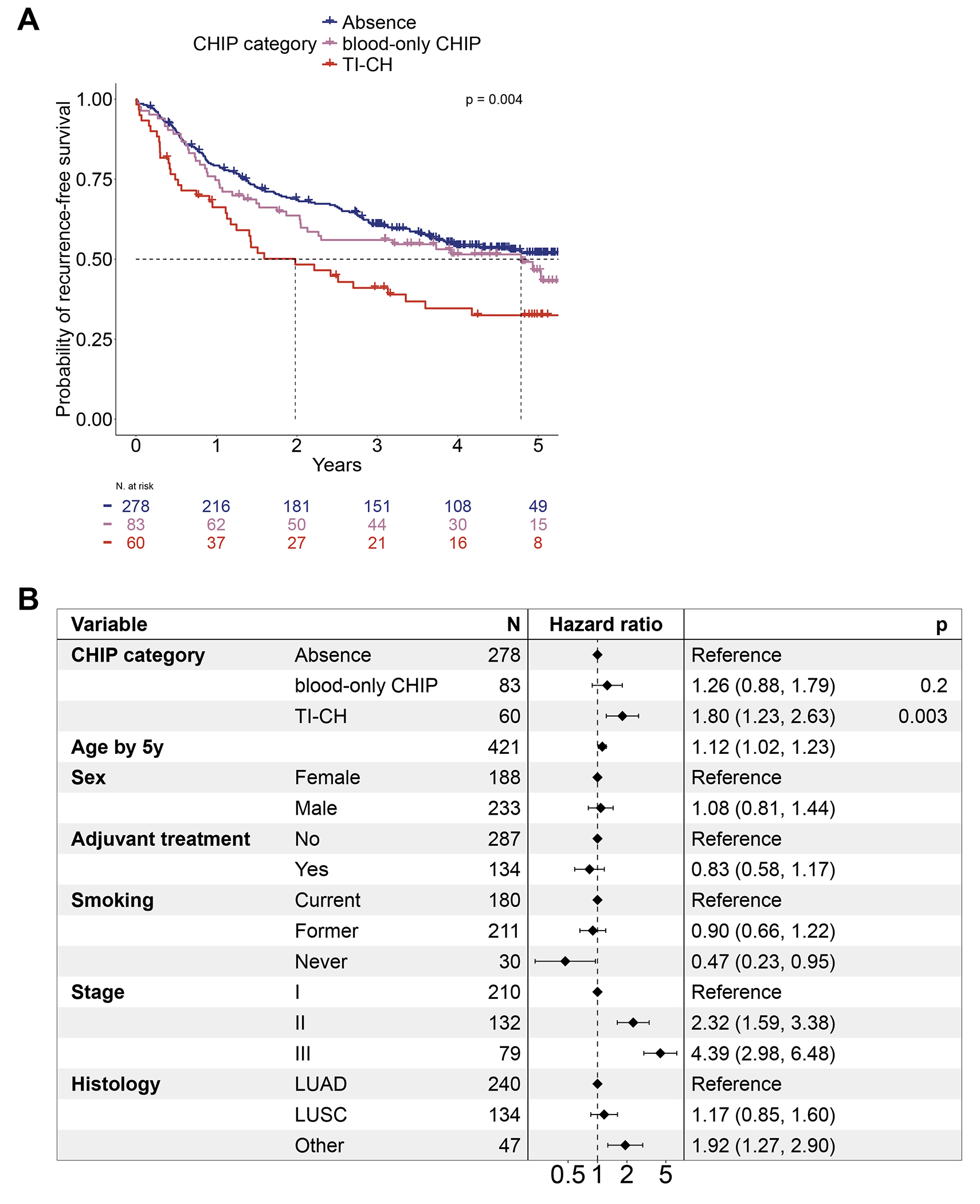
TI-CH and patient outcome in NSCLC **A.** Kaplan-Meir probability estimates of recurrence-free survival across patients without CHIP (blue), patients with blood-only CHIP (purple), and patients with TI-CH (red) in TRACERx. P-value is from the log-rank test. **B.** Multivariable Cox model for recurrence-free survival in TRACERx accounting for baseline clinical parameters and CHIP category (absence, blood-only CHIP, TI-CH). Hazard ratio and 95% confidence intervals are depicted. Age in years was used as a continuous variable, and divided by 5 so that the hazard ratio represents the change in risk for a 5-year increase. P-values derived from the multivariable analysis are shown only for the CHIP categories (the factor of interest). LUAD: lung adenocarcinoma; LUSC: lung squamous carcinoma.

Patients with TI-CH had shorter recurrence-free and overall survival compared to patients without CHIP (HR=1.84, 95% CI 1.28-2.65, for recurrence-free survival, and HR=2.01, 95% CI 1.37-2.95, for overall survival). Recurrence-free and overall survival did not differ between patients with blood-only CHIP and patients without CHIP. Across the three categories of patients, the median recurrence-free survival was 6.0 years (95% CI 3.84-not reached), 4.8 years (95% CI 2.1-not reached), and 2.0 years (95% CI 1.2-3.6), respectively (Fig 2A). The median overall survival was 6.0 years (95% CI 5.5-not reached), 4.9 years (95% CI 3.7-not reached), and 3.1 years (95% CI 1.6-not reached), respectively (Fig S13).

In a multivariable analysis, TI-CH was an independent predictor of shorter recurrence-free survival (HR=1.80, 95% CI 1.23-2.63) compared to patients without CHIP, while blood-only CHIP was not (HR=1.26, 95% CI 0.88-1.79) (Fig 2B). In the subset of patients with CHIP, the adjusted hazard ratio comparing TI-CH with blood-only CHIP was 1.62 (95% CI 1.02-2.56) (Fig S14A). These associations were independent of NSCLC genetic drivers and correlates of immune infiltration, including tumor purity, tumor-infiltrating neutrophils, and the proportion of myeloid cells (Fig S14B–D).^28^ We further recapitulated these associations using an alternative metric of TI-CH that estimates the fraction of non-tumor cells harboring CHIP mutations (Supp Methods, Fig S14E). TI-CH was also an independent predictor of the risk of all cause-mortality, lung-cancer related death, and tumor recurrence or new primary lung cancer in TRACERx (Fig S15).

Given the known association between CHIP VAF and disease outcomes,^6,14,15^ we further confirmed the prognostic value of TI-CH relative to blood-only CHIP using the ratio of tumor over blood VAF of CHIP mutations. This ratio was an independent predictor of shorter recurrence-free survival (Fig S16), suggesting that the impact of TI-CH on outcomes is not merely due to clonal expansions in the blood, and the degree of CHIP-derived tumor infiltration might influence disease outcomes.

In the MSK-IMPACT stage I-III NSCLC validation cohort, the adjusted hazard ratio for the risk of all-cause mortality for patients with TI-CH was 1.35 (95% CI 1.03-1.77) compared to patients without CHIP and was 1.20 (95% CI 0.94-1.52) for patients with blood-only CHIP (Fig S17).

### Tumor-infiltrating clonal hematopoiesis across cancer types

**Figure 3.**
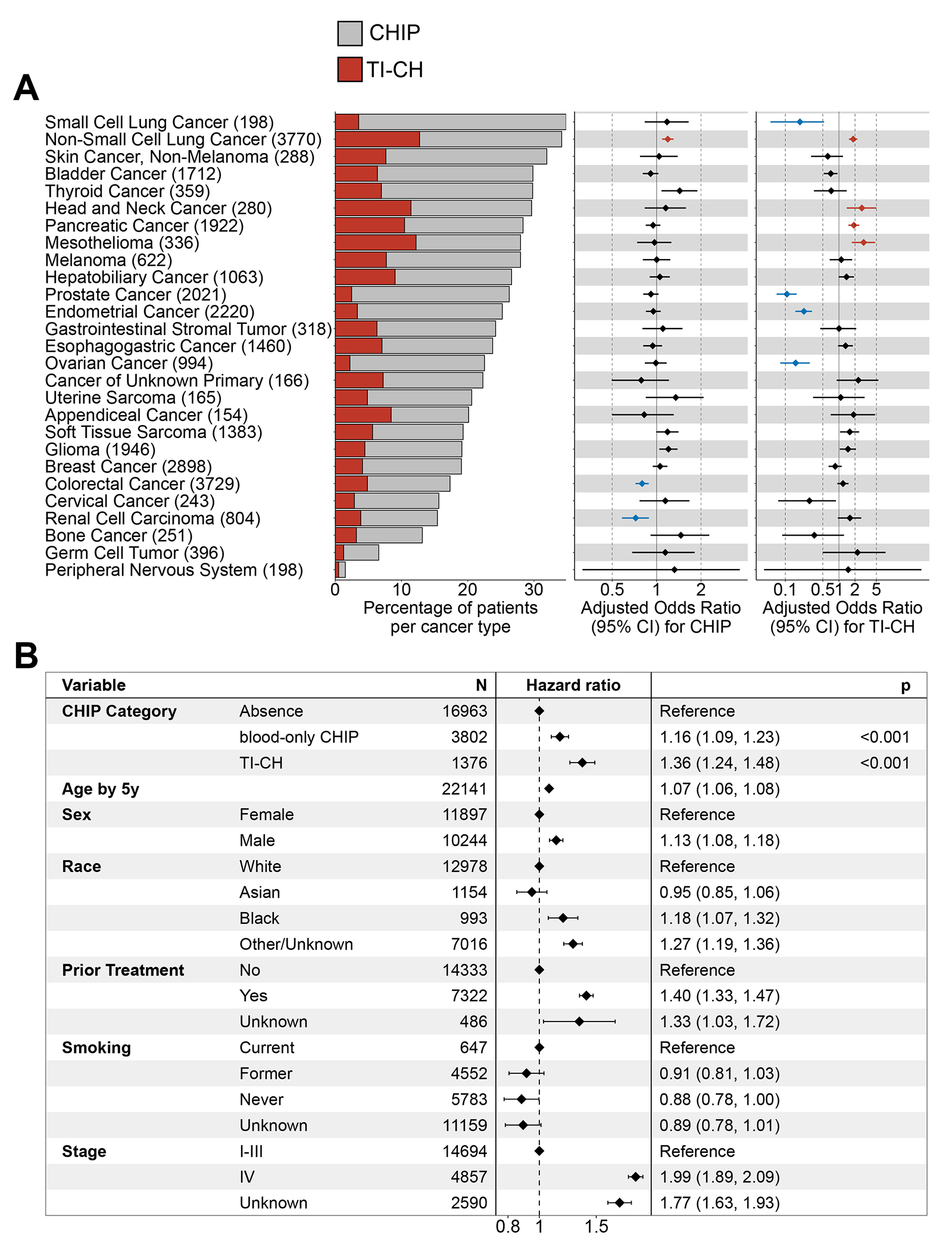
TI-CH in the pan-cancer primary MSK-IMPACT cohort. **A. Left:** Proportion of patients with CHIP (gray) and with TI-CH (red) across cancer types. The 31,556 patients with primary tumor samples were selected, including only cancer types with more than 150 patients. **Right:** adjusted odds ratio and 95% confidence intervals (CI) for the presence of CHIP and TI-CH in each cancer type compared to the others. Odds ratios were derived from multivariable logistic regressions, adjusting for age, sex, ethnicity, smoking status, prior treatment (CHIP and TI-CH) as well as tumor purity and the blood VAF of CHIP mutations (TI-CH). Benjamini-Hochberg correction for multiple testing was applied, with enrichments (q<0.05) highlighted in red and blue for positive and negative associations, respectively. **B.** Multivariable Cox model for the risk of death in 22,141 patients with primary tumor samples analyzed and overall survival available. Hazard ratio and 95% confidence intervals are depicted. Age in years was used as a continuous variable, and divided by 5 so that the hazard ratio represents the change in risk for a 5-year increase. P-values derived from the multivariable analysis are shown only for the CHIP categories (the factor of interest).

We investigated CHIP and TI-CH in 31,556 patients with matched blood and primary tumor samples across 75 cancer types (Table S4 and Fig S18–S19).^23,24^ CHIP was observed in 24% (7,450/31,556) of patients with a 5.3% median blood VAF (IQR 3.1-11.9). Adjusting for baseline parameters (see Methods), NSCLC was enriched for CHIP compared to other tumor types. In contrast, colorectal cancer and renal cell carcinoma had reduced likelihood of CHIP (Fig 3A).

Among patients with CHIP, 26% had TI-CH (1,974/7,450; range 9-42% across cancer types). Adjusting for baseline parameters, tumor purity and blood VAF of CHIP mutations, NSCLC, head and neck cancer, pancreatic cancer, and mesothelioma were enriched for TI-CH compared to other tumor types. Prostate, endometrial, ovarian, and small-cell lung cancers had reduced likelihood of TI-CH (Fig 3A).

We assessed the associations between TI-CH and outcome in 22,141 patients with available overall survival data. In a multivariable analysis, the risk of all-cause mortality associated with blood-only CHIP was 1.16 (95% CI 1.09-1.23) and for TI-CH was 1.36 (95% CI 1.24-1.48) relative to patients without CHIP (Fig 3B). TI-CH conferred a 1.17 (95% CI 1.06-1.29) greater risk of all-cause mortality relative to blood-only CHIP. In the subset of 14,694 patients with stage I-III disease, the adjusted hazard ratio for the risk of all-cause mortality for patients with TI-CH was 1.44 (95% CI 1.28-1.62) compared to patients without CHIP (Fig S20), and 1.25 (95% CI 1.10-1.42) compared to patients with blood-only CHIP. As in our discovery cohort (Fig S16), the tumor over blood VAF ratio of CHIP mutations was an independent predictor of shorter overall survival (Fig S21), suggesting that these associations were not solely the result of variations in the burden of CHIP in the blood. TI-CH remained an independent predictor for the risk of death after accounting for cases that subsequently transformed with hematologic neoplasms as censoring events (n=119 cases, treated as censoring events).

TI-CH was prevalent in metastatic samples. CHIP was observed in 26% (4,686/17,795) of patients with matched blood and metastatic tumors sampled from various organ sites, of which 28% (1,304/4,686) had TI-CH in the metastatic samples. For CHIP-positive patients with NSCLC, hepatobiliary cancer, and renal cell carcinoma, more than 35% of metastatic samples analyzed exhibited TI-CH (Fig S22).

In 54 primary tumors with TI-CH and their paired progression metastases in MSK-IMPACT (Fig S23A), 81% (44/54) of metastases exhibited TI-CH. The median CHIP mutation VAF was higher in metastases compared to primaries (6.1% vs. 4.3%). We further tracked TI-CH in 49 metastases obtained at progression or autopsy from two TRACERx patients with TI-CH-positive primary tumors enrolled in PEACE (Fig S23B), the first with *DNMT3A* and *TET2* mutations, and the second with a *DNMT3A* mutation. In both patients, the mutations were detected (without a VAF threshold) in all metastases, except for two located in the brain. In the first patient, 81% (18/22) of metastases qualified for TI-CH. In the second patient, all 9 liver metastases qualified for TI-CH, compared to 17% (3/18) of metastases at other organ sites. Collectively, these findings indicate that TI-CH recurs in metastases.

### *TET2*-mutant CHIP as a predictor of tumor-infiltrating clonal hematopoiesis

**Figure 4.**
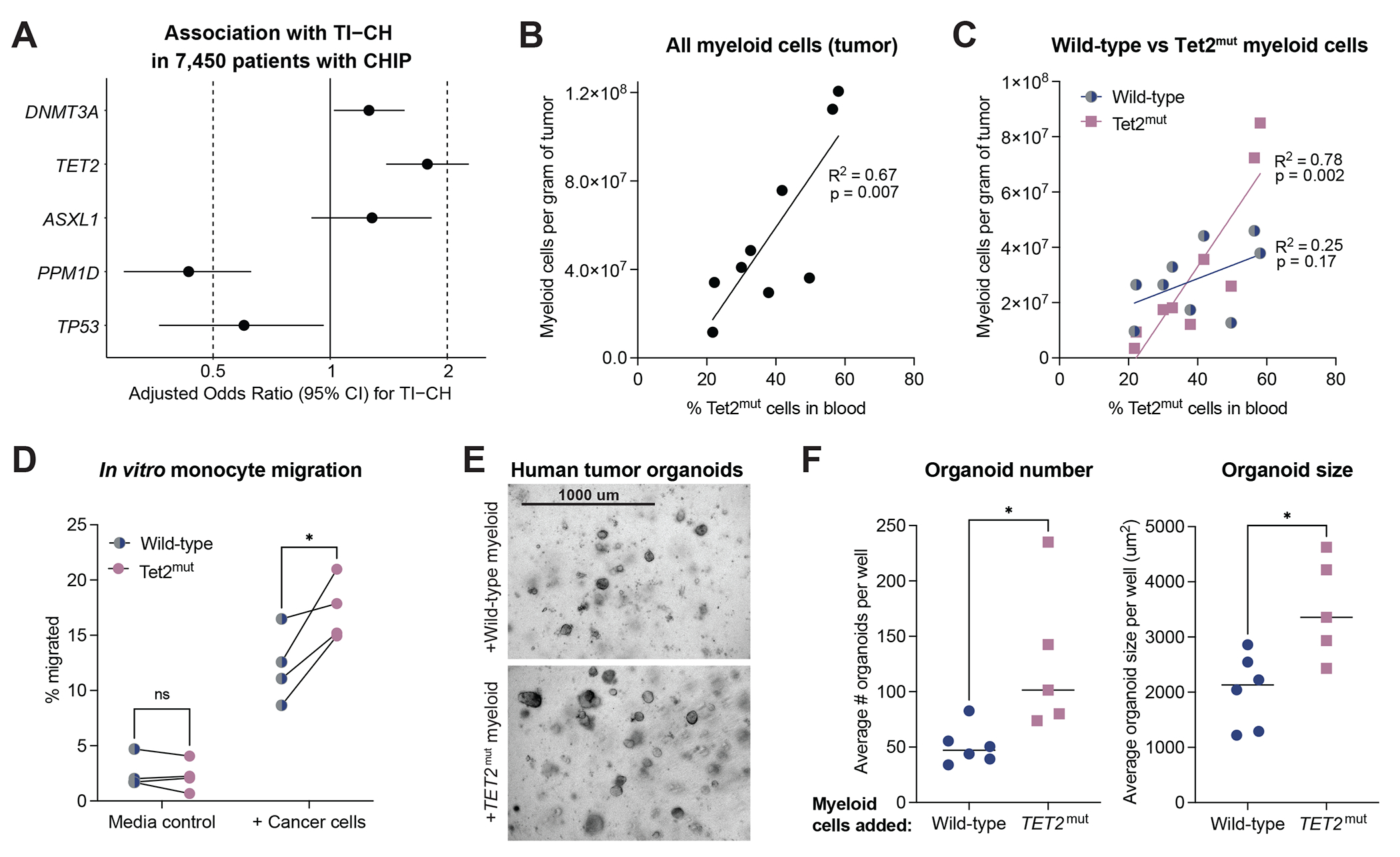
The functional impact of *TET2*-mutant CHIP in NSCLC. **A.** Adjusted odds ratio and 95% confidence intervals (CI) derived from a multivariable logistic regression for the presence of TI-CH in 7,450 patients with CHIP in the MSK-IMPACT cohort, adjusting for age, sex, ethnicity, prior treatment, smoking status, tumor purity, and the blood VAF of CHIP mutations. CHIP mutations were categorized as *DNMT3A, TET2, ASXL1, PPM1D, TP53*, or other gene mutations, with the “other” category used as the reference level. **B-C.** Correlation between percent of Tet2-mutant cells in blood and overall myeloid cell infiltration in the tumor microenvironment (**B**) or Tet2-mutant versus wild-type myeloid cell infiltration in the tumor microenvironment (**C**) in mice, using 9 biological replicates. **D.** Percent of Tet2-mutant or wild-type monocytes that migrated to the bottom chamber in the presence or absence of 3LL lung tumor cells (blood from 1-4 mice was pooled per datapoint). *p<0.05. **E.** Representative brightfield images of patient-derived tumor organoids after 14-day co-culture with *TET2*-mutant or wild-type human myeloid cells. **F.** Number and size of tumor organoids after 14-day co-culture with *TET2*-mutant or wild-type human myeloid cells (n=5-6 biological replicates across three independent experiments). *p<0.05.

Similar to the NSCLC validation cohort (Fig S11), patients with *TET2*-mutant CHIP from the pan-cancer cohort had the highest frequency of TI-CH (33%, 381/1191), followed by *ASXL1*-mutant (32%, 124/392), *DNMT3A*-mutant (25%, 926/3,753), and *PPM1D*-mutant CHIP (13%, 83/622) (Fig S24). *TET2* mutations had the largest relative tumor-infiltrating clone size compared to other CHIP gene mutations (median tumor over blood VAF ratio of 0.18 versus 0.13, p<0.001) (Fig S24). To assess whether distinct CHIP driver genes were differentially associated with TI-CH, we performed a multivariable analysis adjusted for baseline parameters, tumor purity and blood VAF of CHIP mutations (Fig S24D). *TET2* mutations were an independent positive predictor of TI-CH compared to other CHIP mutations (adjusted OR=1.78, 95% CI 1.39-2.27), while the opposite was observed for *PPM1D* mutations (adjusted OR=0.43, 95% CI 0.29-0.63) (Fig 4A). Determining genotype-specific risks associated with TI-CH for clinical outcomes will require larger cohorts.

### Impact of *TET2*-mutant CHIP on the lung tumor microenvironment

Given the preponderance of *TET2*-mutant cells to infiltrate tumors (Fig 4A), we explored the functional link between *TET2*-mutant CHIP and lung TI-CH using mouse models. Tet2-mutant CHIP mice were generated by transplanting a 1:1 mix of congenically-marked Tet2-mutant and wild-type bone marrow cells into busulfan-conditioned wild-type recipients (Fig S25A).^30^ Consistent with prior studies,^34^ Tet2-mutant cells expanded significantly more in blood relative to wild-type counterparts (Fig S25B–C). We induced lung tumors in Tet2-mutant CHIP mice by orthotopically transplanting Kras^G12C^ 3LL LUAD cells, and analyzed blood, tumor and adjacent lung tissues (Fig S26A–C, S27A).^31^ Similar to our human results (Fig 1E), in mouse tumors Tet2-mutant cells were enriched within the myeloid and natural killer cell compartments relative to other cell populations (Fig S27B).

Consistent with the clinical association between CHIP and a myeloid-rich lung tumor microenvironment (Fig 1D), the percentage of Tet2-mutant cells in blood positively correlated with the overall myeloid infiltration in the tumor (Fig 4B). The correlation was driven by Tet2-mutant tumor-infiltrating myeloid cells (Fig 4C), including mononuclear phagocytes (monocytes, macrophages and dendritic cells) and granulocytes (neutrophils and eosinophils) (Fig S27C–D), although the former were affected more profoundly by blood CHIP levels (Fig S27E).

In a functional migration assay,^35^ blood-derived Tet2-mutant monocytes migrated to 3LL LUAD cells significantly more than their wild-type counterparts (Fig S27F, 4D). In contrast, neutrophil migration was not affected by Tet2 status (Fig S27G). *In vivo* quantification of Tet2-mutant versus wild-type myeloid cell subsets revealed that Tet2-mutant CD11b^+^ monocyte-derived macrophages preferentially accumulated in the tumor relative to adjacent normal lung (Fig S27H), an effect that was not observed for their monocyte precursors or neutrophils (Fig S27H–I). These data suggest that Tet2-mutant monocytes preferentially migrate towards lung cancer cells and accumulate as CD11b^+^ monocyte-derived macrophages in the tumor microenvironment.

### Impact of *TET2*-mutant myeloid cells on lung tumor cell growth

To evaluate whether *TET2*-mutant myeloid cells influence lung tumor cell growth, we cultured TRACERx patient-derived LUAD organoids^36^ with human *TET2*-mutant or wild-type myeloid cells from lungs of humanized mice engrafted with *TET2*-mutant or wild-type hematopoietic stem cells (Fig S28A–B).^32^ Co-cultures of tumor organoid cells with *TET2*-mutant myeloid cells resulted in larger and more numerous organoids compared to co-cultures with wild-type myeloid cells (Fig 4E–F). Thus, *TET2* mutations in myeloid cells can functionally impact tumor cell clonogenicity and growth.

## DISCUSSION

Cancer evolution, once regarded as a step-wise accumulation of oncogenic mutations, is now seen as a multifactorial process impacted by the tissue microenvironment, local and systemic immune responses, environmental exposures and aging.^37^ Recent studies have highlighted the influence of the aging hematopoietic system on lung cancer development.^38^ Here, age-associated tumor-infiltrating clonal hematopoiesis, or TI-CH, emerges as an important facet influencing cancer progression.

TI-CH is a pan-cancer phenomenon, observed in 25% of patients with CHIP, representing over 6% of all solid tumor cases, and associated with adverse patient outcomes. The likelihood of having TI-CH varies across cancer types, with enrichment in NSCLC,^18^ head and neck cancer, pancreatic cancer, and mesothelioma. In NSCLC, approximately 40% of patients with CHIP had TI-CH, which independently increased their risk of death or recurrence. Across CHIP mutations, *TET2* emerged as an independent predictor of TI-CH. In a NSCLC model, Tet2-mutant CHIP caused increased myeloid cell infiltration into tumors, mimicking observations in human disease. Tet2-mutant monocytes preferentially migrated towards tumor cells and accumulated as macrophages within murine tumors. Consistent with published evidence that *TET2* mutations can functionally remodel myeloid responses,^39^ *TET2*-mutant myeloid cells displayed enhanced protumorigenic capacity in tumor organoid co-cultures compared to wild-type myeloid cells.

The associations between TI-CH and adverse outcomes, the increased odds of TI-CH with *TET2*-mutant CHIP, and the impact of *TET2*-mutant immune cells on the tumor microenvironment and cancer cell growth, support the role of TI-CH in tumor evolution and its potential utility in cancer diagnostics.^18^ Future studies should evaluate these findings in larger and more ethnically diverse cancer cohorts and further interrogate the functional impact of TI-CH on tumor progression.

CHIP is linked to cardiovascular disease, where mutant myeloid cells produce proinflammatory cytokines, accelerating atherosclerosis.^7,40–42^ Tet2-mutant macrophages were shown to secrete higher levels of IL-1β, and IL-1β inhibition reduced the risk of cardiovascular complications.^7,40–42^ Interestingly, anti-IL1β therapy reduces the incidence of lung cancer,^43^ particularly in patients with *TET2*-mutant CHIP.^44^ However, clinical trials have shown that anti-IL-1β therapy is ineffective in established NSCLC.^45^ Several other cytokines upregulated in Tet2-mutant macrophages, including IL-6, PF4, CXCL1, CXCL2 and OSM,^7,40^ are implicated in promoting lung tumor progression in model systems.^46–49^ Understanding the impact of TI-CH on myeloid-cancer cell communication in solid cancer initiation and growth may be crucial for therapeutic interception.

The ability of age-associated somatic clonal expansions in one tissue, such as the hematopoietic compartment, to regulate malignant progression in another tissue represents a new perspective in cancer biology. Given the pervasiveness of somatic mosaicism in humans,^50^ more examples of the interplay between non-malignant somatic clones, aging, and human disease may emerge, opening a role for prevention strategies attenuating somatic clonal proliferation and tissue inflammation.

## Supplementary Material

Supplement

## Figures and Tables

**TABLE 1:** Characteristics of the TRACERx and MSK-IMPACT cohort of stage I-III NSCLC. CHIP refers to CHIP in the blood.

	TRACERx Primary Stage I-III NSCLC (n=421)		MSK-IMPACT Primary Stage I-III NSCLC (n=2,602)	
	No CHIP	CHIP	No CHIP	CHIP
**Characteristics**	278 (66%)	143 (34%)	1,685 (65%)	917 (35%)
**Age in years, median (IQR)**	68 (62-74)	73 (66-78)	67 (60-73)	72 (66-77)
**Sex, No. (%)**				
Female	130 (47%)	58 (41%)	843 (50%)	441 (48%)
Male	148 (53%)	85 (59%)	524 (32%)	286 (31%)
Missing	-	-	318 (19%)	190 (21%)
**Smoking status, No. (%)**				
Current smoker *median pack-years (IQR)*	125 (45%) *42 (30-54)*	55 (38%) *45 (30-57)*	63 (4%)	28 (3%)
Former smoker *median pack-years (IQR)*	130 (47%) *36 (19-52)*	81 (57%) *40 (21-62)*	484 (29%)	270 (29%)
Never smoker	23 (8%)	7 (5%)	152 (9%)	51 (6%)
Missing	-	-	986 (58%)	568 (62%)
**Race, No (%)**				
White	267 (96%)	137 (96%)	868 (52%)	497 (54%)
Asian	2 (1%)	0 (0%)	86 (5%)	28 (3%)
Black	4 (1%)	3 (2%)	46 (3%)	19 (2%)
Other/Missing	5 (2%)	3 (2%)	685 (41%)	373 (41%)
**Histology, No (%)**				
LUAD	160 (58%)	80 (56%)	1264 (75%)	685 (75%)
LUSC	84 (30%)	50 (35%)	222 (13%)	132 (14%)
Other	34 (12%)	13 (9%)	199 (12%)	138 (11%)
**Main oncogenic driver, No (%)**				
*TP53* mutation	176 (63%)	103 (72%)	742 (44%)	407 (44%)
*KRAS* mutation	87 (31%)	40 (28%)	484 (29%)	294 (32%)
*STK11* mutation	37 (13%)	26 (18%)	201 (12%)	117 (13%)
*EGFR* mutation	22 (8%)	7 (5%)	387 (23%)	193 (21%)
Oncogenic isoforms^$^	15 (5%)	4 (3%)	70 (4%)	16 (2%)
**Follow-up in years, median (95% CI)**	4.6 (4.5-4.8)	4.9 (4.5-5.1)	2.4 (2.3-2.6)	2.4 (2.2-2.7)
**TI-CH, No (%)**	-	60 (42%)	-	333 (36%)

$: *RET, ROS1, ALK* and *MET* oncogenic isoforms

IQR: interquartile range; No: number; CI: confidence interval.
