# Supplementary material for: Tumor-infiltrating clonal hematopoiesis
Source: N Engl J Med. Author manuscript; Available in PMC 2025 Apr 24. (PMC12021423; doi:10.1056/NEJMoa2413361)
Supplement: Supplement [file NIHMS2050705-supplement-Supplement.pdf]

Supplementary Appendix

Supplement to: Pich O.*, Bernard E.*, Zagorulya M.*, et al. **Tumor-infiltrating clonal hematopoiesis.**

1. Supplementary Methods
    1.1. Study cohorts
        1.1.1. TRACERx cohort of stage I-IIIA non-small cell lung cancer
        1.1.2. MSK-IMPACT cohort
    1.2. CHIP and TI-CH calling
        1.2.1 Variant calling
        1.2.2. CHIP annotation
        1.2.3. Genotyping CHIP mutations in tumor sequencing data
        1.2.4 Normal Cell Fraction (NCF) of a CHIP mutation
    1.3. Reanalysis of published data from Alvarez-Prado et al.15
    1.4. Statistical analyses
    1.5. Single-cell genotyping of tumor immune infiltrate from CHIP patient
        1.5.1. Lung tumor tissue processing into single cell suspensions
        1.5.2. Single-cell FACS-sorting
        1.5.3. Single-cell genotyping
        1.5.4. Single-cell genotyping analysis
    1.6. Lung tumor studies in murine model of Tet2mut CHIP
        1.6.1. Murine model of Tet2mut CHIP
        1.6.2. 3LL LUAD cell line and tumor injections
        1.6.3. Blood and tissue harvest and processing
        1.6.4. Flow cytometry analysis
        1.6.5. Ex vivo migration assay
    1.7. Co-culture of patient-derived tumor organoids with TET2mut myeloid cells
        1.7.1. Lung tumor organoid establishment and passaging
        1.7.2. Humanized mouse model of TET2mut CHIP
        1.7.3. Isolation of human lung myeloid cells
        1.7.4. Tumor:myeloid cell co-culture
2. Supplementary Figures
3. Supplementary Tables
    3.1. Table S1
    3.2. Table S2
    3.3. Table S3
    3.4. Table S4
4. Supplementary References

1. Supplementary Methods

1.1. Study cohorts

1.1.1. TRACERx cohort of stage I-IIIA non-small cell lung cancer

Human subjects

The study participants were enrolled in the Tracking Cancer Evolution through Therapy (TRACERx) clinical study (NCT01888601), approved by an independent ethics committee (13/LO/1546). All participants provided written informed consent before joining the study. The TRACERx 421 cohort represents the first 421 patients with treatment-naive stage IA-IIIA non-small cell lung cancer (NSCLC) prospectively recruited in TRACERx (**Table 1**). Eligible patients were over 18 years (median 69, range 34-92) and had not received prior chemotherapy or neoadjuvant chemotherapy for NSCLC. Histological subtypes included 240 patients with lung adenocarcinoma (LUAD) and 134 with lung squamous carcinoma (LUSC). Detailed cohort characteristics have been previously described.[1]

Tissue collection and processing

Primary NSCLC samples were obtained from surgical resections. Tumor tissue was collected in Advanced DMEM/F12 medium (Gibco) supplemented with 2 mM Ultraglutamine I (Lonza), 10 mM HEPES (Gibco), 100/100 U/mL Penicillin/Streptomycin (Gibco), 1x Primocin (Invivogen) and transported on ice to the laboratory.

Paired blood and tumor samples from TRACERx patients included in this study were profiled by whole exome sequencing (WES), with a median depth of 465X (range 84-1000) and 467X (range 57-1398), respectively.

The different data modalities from TRACERX used in this study are described in **Figure S1A**. Genomic data analysis from the TRACERx 421 cohort has been previously described.[1] Multiplex imaging mass cytometry data analysis of tumor samples from the TRACERx 421 cohort has also been previously described.[2]

1.1.2. MSK-IMPACT cohort

Tumor samples (31,556 primary tumor samples and 17,795 metastatic tumor samples) with matched blood samples from 49,351 patients sequenced at Memorial Sloan Kettering Cancer Center, using the Memorial Sloan Kettering Integrated Molecular Profiling of Actionable Cancer Targets (MSK-IMPACT) clinical sequencing assay, were included in the study.[3] Tumor types were classified using the OncoTree classification system.[4] The cohort spanned 75 cancer types under this system using the “tissue” level. Clinical and genomic characteristics have been previously described.[5–8] The pan-cancer cohort included a cohort

of 2,602 patients with stage I-III NSCLC that was used as a validation cohort. This study was approved by the MSK Cancer Center Institutional Review Board (IRB number 23-345).

1.2. CHIP and TI-CH calling

1.2.1 Variant calling

We performed somatic variant calling to identify CHIP mutations in blood samples. Pipelines were run uniformly across the TRACERx and MSK-IMPACT cohorts. Variant calling was conducted using `MuTect2` (version 4.2), part of the Genome Analysis Toolkit (GATK) suite. `MuTect2` was run in "tumor-only" mode, i.e. treating each blood sample as a single sample without a matched reference sample. GnomAD was used as a germline resource. The software was run with the following options:

```
gatk --java-options "-Xmx7g" Mutect2 -R $FASTA_FILE -I $ID_BAM -tumor
$SAMPLE_NAME --germline-resource $GERMLINE_RES -O $SAMPLE_NAME.vcf.gz
--callable-depth 3 --f1r2-tar-gz f1r2.tar.gz -L $gatk_bedfile

gatk --java-options "-Xmx7g" LearnReadOrientationModel -I f1r2.tar.gz -O
read-orientation-model.tar.gz

gatk --java-options "-Xmx7g" GetPileupSummaries -I $ID_BAM -V $path_small_exac
-L $path_small_exac -O getpileupsummaries.table

gatk --java-options "-Xmx7g" CalculateContamination  -I
getpileupsummaries.table -tumor-segmentation segments.table -O
contamination.table

gatk --java-options "-Xmx7g" FilterMutectCalls -V $SAMPLE_NAME.vcf.gz
--tumor-segmentation segments.table -R $FASTA_FILE --contamination-table
contamination.table --ob-priors read-orientation-model.tar.gz
--max-events-in-region 3 -O $SAMPLE_NAME.filtered.vcf.gz
```

1.2.2. CHIP annotation

Mutations were annotated using `VEP 109`, using the following command:

```
vep --species homo_sapiens --assembly GRCh37 --dir_cache $dir_cache --offline
--cache --compress_output gzip --vcf  --format vcf --protein --force_overwrite
--symbol --no_intergenic --pick --af_gnomade --af_gnomadg --canonical --fasta
$fa --fork 4
```

Candidate somatic mutations were selected if they were passed or were flagged for quality with a maximum of one `MuTect2` filter. Artifacts and germline calls were filtered out using the calculated recurrences of the mutations, distributions of variant allele frequency (VAF), and occurrences in gnomAD.

CHIP mutations were then annotated from a consensus list of 77 myeloid driver genes, derived from a set of published resources.[7,9–12] Using those resources, consensus rules for CHIP driver mutations were defined for each gene based on the type of mutations (frameshift, nonsense, splice-site, non-synonymous) and/or the affected amino acid or protein domain (**Table S1**). All candidate CHIP mutations were further manually curated. CHIP was defined using a 2% VAF cutoff on the annotated mutations.

1.2.3. Genotyping CHIP mutations in tumor sequencing data

To genotype patient-specific CHIP mutations derived from the blood samples (see above) in the aligned sequencing data from the TRACERx and the MSK-IMPACT tumor samples, we utilized the `bam2R` function (within the R package `deepSNV`, v1.44). The software was run with a minimum phred quality of 25. Secondary alignments were removed (mask=256), and only paired reads were kept (keepflag=1). A minimum of 2 supporting reads was required for evidence of the presence of the CHIP mutations in the tumor samples. Furthermore, tumor-infiltrating clonal hematopoiesis (TI-CH) was deemed present if the VAF in the tumor samples was higher than 2%.

Our genotyping method was not biased by tumor sequencing coverage and demonstrated a >99% specificity (**Fig S7**). We validated the detection of CHIP mutations in blood and in the matched tumors for 112 samples using digital droplet PCR (**Fig S8**).

1.2.4 Normal Cell Fraction (NCF) of a CHIP mutation

The bulk tumor VAF does not directly reflect the proportion of mutant hematopoietic cells within the tumor microenvironment, as tumor VAF also depends on the total proportion of hematopoietic cells (both mutant and wild type), and of the copy-number states at the genomic locus of the CHIP mutation.

We derived the mathematical formula to calculate the proportion of mutant non-tumoral cells within the tumor microenvironment. We termed this value NCF (normal cell fraction), analogous to the concept of “cancer cell fraction” in cancer genomics.[13] NCF is a function of the bulk tumor VAF, the tumor purity (i.e., the proportion of tumoral cells within the bulk sample), and the copy-numbers of tumor and normal cells at the mutation position. We estimated tumor purity (ρ) and tumor copy-number (c) in TRACERx using state-of-the-art copy-number algorithms.[14] We reasonably assumed that normal cells were diploid at the locus of the CHIP mutation. A schematic illustrating the concept of NCF and its mathematical derivation is provided below:

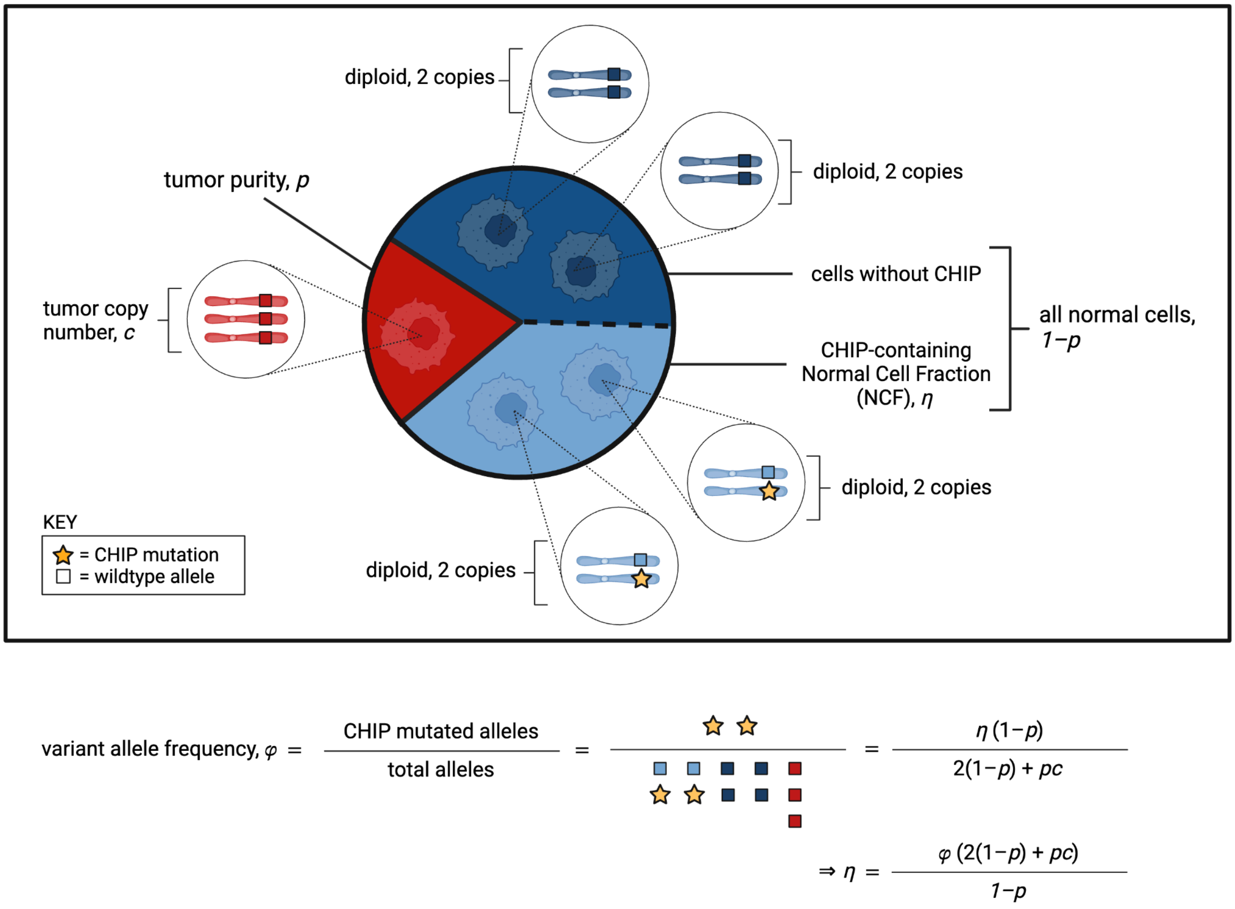

Estimating the Normal Cell Fraction (NCF) of a CHIP mutation. In a sample comprising both tumor (red) and normal (light and dark blue) cells, the tumor purity describes the proportion of tumor cells, such that the proportion of normal cells is equal to 1- ρ. The fraction of normal cells containing a CHIP mutation (light blue) over all normal cells is denoted by the NCF. All normal cells are assumed to be diploid, and a single copy of a CHIP mutation is assumed to be harbored by each CHIP-containing normal cell. In tumor cells, the copy number (c) describes the number of copies of the corresponding genomic locus. As such, the variant allele frequency (VAF) of a CHIP mutation is defined by dividing the number of CHIP mutated alleles by the total number of alleles (yellow stars indicate CHIP mutated alleles and colored squares indicate wildtype alleles) at that genomic locus across the entire sample. Therefore, we can model the NCF as a function of VAF, tumor purity, and copy number of the corresponding genomic locus in tumor cells.

1.3. Reanalysis of published data from Alvarez-Prado et al.[15]

Fastq files from the blood and tumor sequencing data were aligned to the hg38 reference genome using `bwa-mem` (v0.7.10). Duplicated reads were marked using `MarkDuplicates` from Picard tools, and `BQSR` was applied following `GATK4` best practices.[16] The same analysis pipelines described in sections 1.2.1 and 1.2.2 were applied to identify patients with CHIP.

Fastq files for FACS sorted transcriptomic data for the patient with *TET2*-mutant CHIP were trimmed using `fastp` (v0.23) and aligned to the hg38 reference genome using a 2-pass `STAR` (v2.7.11, with GTF from GENCODE v42) alignment using the following parameters:

```
STAR --runThreadN 16 --genomeDir $INDEX_GENOME --outSAMunmapped Within
--outFilterType BySJout --outFilterMultimapNmax 20 --outFilterMismatchNmax 999
--outFilterMismatchNoverLmax 0.04 --alignIntronMin 20 --alignIntronMax 1000000
--alignMatesGapMax 1000000 --alignSJoverhangMin 8 --alignSJDBoverhangMin 1
--sjdbScore 1 --limitBAMsortRAM=42589777693 --limitSjdbInsertNsj=2000000
--readFilesIn $SAMPLE_NAME.R1.trimmed.gz $SAMPLE_NAME.R2.trimmed.gz
--outSAMattrRGline $RGrup --outSAMtype BAM Unsorted --outFileNamePrefix
$SAMPLE_NAME"." --quantMode TranscriptomeSAM --outSAMattributes All
--readFilesCommand zcat
```

Duplicated reads were marked using `GATK4`. Finally, the TET2 mutation was genotyped in the transcriptomic data as described in section 1.2.3.

1.4. Statistical analyses

Distributions of categorical and continuous variables between two subgroups were compared using Fisher's exact test and the Wilcoxon rank sum test, respectively. Odds ratios for the presence of CHIP or TI-CH were derived from multivariable logistic regression adjusted for age, sex, ethnicity, treatment, smoking status, tumor stage, tumor purity, and blood VAF of the CHIP mutations. Two-sided p-values <0.05 were considered statistically significant. P-values were corrected for multiple testing with the Benjamini-Hochberg procedure when appropriate.

In TRACERx, overall survival (OS) and recurrence-free survival (RFS) were measured from the time of study entry to the time of the event (death from any cause for OS, tumor recurrence or death from any cause for RFS) or last follow-up. In MSK-IMPACT, OS was measured from the time of blood draw to death from any cause or last follow-up. OS and RFS probabilities were estimated using the Kaplan-Meier method and comparisons between subgroups were conducted using the log-rank test.

Multivariable Cox proportional hazards models were used to estimate the association between CHIP or TI-CH with the elapsed time for the events of interest (e.g. death from any cause, or recurrence or death from any cause), when adjusting for age, sex, ethnicity, treatment, smoking status, and tumor stage. In TRACERx, cause-specific Cox models were also performed for the risk of lung-cancer related death, and for the risk of tumor recurrence or a new primary lung cancer. Median follow-up time was estimated using the reverse Kaplan-Meir method.

1.5. Single-cell genotyping of tumor immune infiltrate from CHIP patient

1.5.1. Lung tumor tissue processing into single cell suspensions

Fresh lung tumor samples were washed in PBS, cut into small fragments, and digested using the Tumor Dissociation Kit (Miltenyi) on a gentleMACS system for 60 minutes following manufacturer's instructions. The digested tumor fragments were passed through a 70 µm strainer to generate a single-cell suspension and centrifuged at 300 g for 4 min at room temperature. If pellets were red, cells were incubated in red blood cell lysis buffer (8.26 g/L ammonium chloride, 1 g/L sodium bicarbonate, 0.1 mM EDTA in distilled water) for 5 min at room temperature, followed by two washes with PBS. Cell suspensions were frozen in ice cold fetal bovine serum (FBS) with 10% DMSO or Recovery Cell Culture Freezing medium (Gibco) in a Mr. Frosty freezing container at -80°C and transferred to liquid nitrogen the next day.

1.5.2. Single-cell FACS-sorting

Cryopreserved healthy donor (HD) peripheral blood mononuclear cells and TRACERx lung tumor single-cell suspensions were rapidly thawed and diluted with FACS media: 10 mL warm IMDM (Iscove's Modified Dulbecco's Medium) supplemented with 10% FBS. Samples were spun down for 5 min at 400 g, washed and incubated with 100 µg/mL DNase (Sigma-Aldrich) for 15 min at room temperature. Cell suspensions were filtered using 100 µm strainers as needed to remove cell aggregates. Lung tumor immune infiltrate was isolated from lung tumor samples by magnetic enrichment using human CD45 (TIL) MicroBeads (Miltenyi Biotec, cat# 130-118-780) following manufacturer's instructions. For FACS staining, cells were first incubated with Fc block (BD #564219, 1:100) for 10 min on ice, and then stained with an antibody cocktail to capture cell markers of interest: CD45-FITC (clone HI30, Biolegend #304006, 1:100), CD19-PE (clone HIB19, Biolegend #302208, 1:100), CD3-BV510 (clone SK7, Biolegend cat# 344828, 1:100), CD56-BV605 (clone HCD56, Biolegend #318334, 1:50), CD14-PE-Daz594 (clone M5E2, Biolegend #301852, 1:100), CD206-PE-Cy7 (clone 19.2, ThermoFisher #25-2069-42, 1:100), CD11b-APC-Cy7 (clone ICRF44, Biolegend #301342, 1:100), HLA-DR-AF700 (clone L243, Biolegend #307626, 1:100) in FACS media for 30 min on ice. Cells were then washed with FACS media and resuspended in FACS media containing 20 nM TOPRO-3 live-dead dye (ThermoFisher #T3605). Single-cell FACS-sorting into 384-well plates was carried out using the MA900 Cell Sorter (Sony Biotechnology) sorter as previously described.[17] Briefly, single cells were directly sorted into 384-well plates containing 2 µL of lysis buffer: Triton X-100 (Sigma-Aldrich #T8787), final concentration 0.18%, Rnase Inhibitor (Takara #2313A) 0.097 U/µL, dNTPs (Life Technologies #19155) 2.4 mM, Barcoded OligodT (custom) 2.4 µM, Protease (Qiagen #19155) 2.7 e-5 AU/µL and snap frozen on dry ice. Flow cytometry data was analyzed using the FlowJo software (version 10.1).

1.5.3. Single-cell genotyping

Single-cell genotyping of the lung tumor immune infiltrate was performed following the TARGET-Seq approach.[17] When possible, primers were designed to amplify both intronic and exonic regions using genomic DNA, mRNA and cDNA as templates. Primers were validated in bulk and at the single-cell level using peripheral blood mononuclear cells, which were also used to determine cDNA amplification cycle number.

384-well plates containing single cell lysate were subject to heat inactivation at 72°C for 15 minutes followed by SMART-Seq 2 cDNA synthesis. The first step was retrotranscription: 5X Buffer (Takara #639537), final concentration 1X, Rnase Inhibitor (Takara #2313A) 1U/μL, LNA-TSO (custom) 1 μM, SMARTScribe Reverse Transcriptase (Takara #639537) 10.3 U/μL, mRNA pre-amplification primers (custom) 718nM , RT-PCR-grade water (Life Technologies #AM9935). RT cycling programme was: 1 cycle 42°C (90min), 10 cycles of 50°C (2min) + 42°C (2min), 1 cycle 70°C (15 min), 4°C hold. Following RT, PCR was performed: 2X Buffer (Takara #638509), final concentration 1X, ISPCR (custom) 50nM, SeqAmp DNA Polymerase (Takara #638509) 2U/μL, gDNA pre-amplification primers (custom) 808nM, cDNA pre-amplification primes (custom) 28.2nM, RT-PCR-grade water (Life Technologies #AM9935). PCR cycling programme was: 1 cycle 98°C (3min), 24 cycles of 98°C (15s) + 67°C (20s) + 72°C (6 min), 1 cycle 72°C (5 min), 4°C hold.

Following cDNA synthesis, Genotyping Stock Plates, consisting of a 1:1 dilution, were made. Genotyping PCR1 reaction was then performed: KAPA2G Robust HS Ready Mix (Roche # KK5702), final concentration 1X, nested genotyping primer (custom) 158nM, RT-PCR-grade dH2O, amplicon mix from Genotyping Stock Plate (1.5 μL).Genotyping PCR1 cycling programme was: 1 cycle 95°C (3min), 20 cycles of 95°C (15s) + 60°C (20s) +72°C (1 min), 1 cycle 72°C (5 min), 4°C hold. Plate barcodes were added at this stage and nested genotyping reactions were separated by primer combination (3 total) for each Genotyping Stock Plate.

Following genotyping PCR1, genotyping PCR2 was performed (volumes reported per well): Buffer 10X (1μL), MgCl2 (1.8μL), DMSO (0.5μL), Nucleotide Mix (0.2μL), Enzyme (0.1μL) (Roche #04-738-292 001), PCR1 amplicons (2.5μL), barcoded PCR2 primers (2μL) (Standard Biotools 100-4876). Well barcodes and P5 and P7 sequences were added during this reaction.

Wells were then pooled and 0.8:1 beads:DNA ratio bead clean-ups were performed for each pooled plate. Libraries underwent quality control checks via Agilent Bioanalyzer (High SensitivityDNA Kit, Agilent, Cat# 5067-4626), TapeStation (High Sensitivity D1000 ScreenTape and Reagents, Agilent, Cat# 5067-5583 and Cat# 5067-5584) and Qubit dsDNA HS Assay Kit (Invitrogen, Cat# Q32854). Each library was diluted to 10nM, pooled in equimolar concentration and further diluted to a 4nM final concentration.

Libraries were sequenced on the MiSeq platform (Illumina) with custom primers using the following configuration: 151 cycles (read1) + 10 cycles (index read) + 151 cycles (read2).

Primers used in the pre-amplification of genomic DNA amplicons

Coordinates (hg38)	Gene	Forward Primer	Reverse Primer
chr4:105,235,136-105,235,670	TET2	ACCACCACCATCACAATTGC	TTCCGCTTGGTGAAAACGAG
chr17: 76,736,568-76,736,987	SRSF2	CTTCGTTCGCTTTCACGACAA	CTTCGTTCGCTTTCACGACAA

Primers used in the pre-amplification of mRNA amplicons

Coordinates (hg38)	Gene	Forward Primer	Reverse Primer
chr4:105,235,136-105,235,670	TET2	ACCACCACCATCACAATTGC	TTCCGCTTGGTGAAAACGAG
chr17: 76,736,324 -76,736,987	SRSF2	CTTCGTTCGCTTTCACGACAA	CTTCGTGCGGATCTGGACTT

Primers used in the pre-amplification of mRNA amplicons

Coordinates (hg38)	Gene	Forward Primer	Reverse Primer
chr4:105,235,136-105,235,670	TET2	AAGCAGTGGTATCAACGCAGAGTACCACCACCATCACAATTGC	AAGCAGTGGTATCAACGCAGAGTTTCCGCTTGGTGAAAACGAG
chr17: 76,736,324 -76,736,987	SRSF2	AAGCAGTGGTATCAACGCAGAGTCTTCGTGCGGATCTGGACTT	AAGCAGTGGTATCAACGCAGAGTCTTCGTTCGCTTTCACGACAA

Forward primers used for target-specific genotyping PCR1 reactions

Target-Specific Forward Primer	Gene	CS1 Adaptor	Forward Barcode*	Genotyping PCR1 Forward Primer
ACCACCTTCCCAGAGTCCTA	TET2	ACACTGACGACATGGTTCTACA	AACGGT	CACTGACGACATGGTTCTACAAACGGTACCACCTTCCCAGAGTCCTA
CTGAGGACGCTATGGATGCC	SRSF2	ACACTGACGACATGGTTCTACA	AACGGT	ACACTGACGACATGGTTCTACAAACGGTCTGAGGACGCTATGGATGCC

CTTCGTTCGCTTTC ACGACAA	SRSF2	ACACTGACGACA TGGTTCTACA	AACGGT	ACACTGACGACATGGTTCTACAAA CGGTCTTCGTTCGCTTTCACGACA A

*Example provided with one plate barcode

Reverse primers used for target-specific genotyping PCR1 reactions

Target-Specific Reverse Primer	Gene	CS2 Adaptor	Reverse Barcode*	Genotyping PCR1 Forward Primer
TTCCGCTTGGTGAA AACGAG	TET2	TACGGTAGCAGA GACTTGGTCT	AAGCCT	TACGGTAGCAGAGACTTGGTCTAA GCCTTTCCGCTTGGTGAAAACGAG
CGCGGACCTTTGTG AGGTCG	SRSF2	TACGGTAGCAGA GACTTGGTCT	AAGCCT	TACGGTAGCAGAGACTTGGTCTAA GCCTCGCGGACCTTTGTGAGGTCG
GAGACCTGGAACGA CTCCGA	SRSF2	TACGGTAGCAGA GACTTGGTCT	AAGCCT	TACGGTAGCAGAGACTTGGTCTAA GCCTGAGACCTGGAACGACTCCGA

*Example provided with one plate barcode

1.5.4. Single-cell genotyping analysis

The analysis was performed as described in https://github.com/albarmeira/TARGET-seq/. Briefly, reads were aligned to the human genome (hg38) and to GENCODEv42 using STAR (version 2.7). Samtools (version 1.19) pileup was used to count the number of reads supporting the alternative and the reference alleles for the mutation loci of interest. A cell was deemed mutant if the coverage at the mutation loci was higher than 50, and the VAF of the mutation when present was between 10% and 90%. Cells with less than 50 reads covering the mutations were removed from the analysis.

1.6. Lung tumor studies in murine model of Tet2^{mut} CHIP

1.6.1. Murine model of Tet2^{mut} CHIP

All animal regulated procedures were approved by The Francis Crick Institute Biological Research Facility Strategic Oversight Committee, incorporating the Animal Welfare and Ethical Review Body, conforming with UK Home Office guidelines and regulations under the Animals (Scientific Procedures) Act 1986 including Amendment Regulations 2012. Animals were housed in individually ventilated cages with unlimited access to food (2018 Autoclavable Rodent Breeding Diet, ENVIGO RMS UK LTD, T.2018S.12) and water with a 12-hour light cycle. C57Bl6/J mice expressing Ptprc^{a}, Ptprc^{b} or Ptprc$^{a/b}$ were obtained from the breeding facility at the Francis Crick Institute. Tet2$^{tm1.2Rao}$ mice were a generous gift from professor Anjana Rao, La Jolla Institute for Immunology, CA, USA.

Busulfan-conditioned bone marrow chimeras were generated as previously described.[18] Briefly, male 10-13 week old congenically-marked wild-type (CD45.1^{+}CD45.2^{+}) recipients

were preconditioned with two 10 mg/kg doses of busulfan (Pierre Fabre "Busilvex") administered 24 hours apart by intraperitoneal injection. 24 hours after the second busulfan dose, $8x10^6$ total bone marrow cells consisting of a 1:1 mixture of $Tet2^{-/-}$ ($CD45.2^+$) and $4x10^6$ wild-type ($CD45.1^+$) cells were transferred intravenously. Donor bone marrow from $Tet2^{-/-}$ ($CD45.2^+$) and congenically-marked wild-type ($CD45.1^+$) mice was isolated by crushing femur and tibia bones in RPMI (Gibco), supplemented with 10% FBS and 100/100 U/ml penicillin/streptomycin (Gibco), using a mortar and pestle. The cell suspension was filtered through a 40 µm strainer and red blood cell lysis was performed using ACK lysing buffer (Gibco) following manufacturer's instructions. Donor bone marrow cells were cryopreserved in FBS with 10% DMSO and stored in liquid nitrogen until the bone marrow transplant. For transplantation, donor bone marrow cells were thawed in warm RPMI media (Gibco) supplemented with 10% FBS and 100/100 U/ml Penicillin/Streptomycin, pelleted by centrifugation at 1250 rpm for 5 min at 4°C and depleted of T cells by staining with biotinylated murine anti-CD3, anti-CD4, and anti-CD8 (Biolegend) in FACS buffer (PBS with 2% FBS and 2mM EDTA (Invitrogen)) for 10 min at room temperature followed by incubation with Streptavidin RapidSpheres 50001 (EasySep) and magnetic enrichment following manufacturer's instructions. After T cell depletion, donor bone marrow cells were washed twice with PBS, counted and resuspended in PBS for injection. Transferred cell engraftment and expansion was evaluated by tail vein blood sampling 6 and 16 weeks after bone marrow chimera establishment.

1.6.2. 3LL LUAD cell line and tumor injections

Murine 3LL LUAD cells were obtained from the Francis Crick Institute Cell Services facility and cultured at 37°C and 5% CO_2 in RPMI (Gibco) supplemented with 10% FBS, 100/100 U/ml penicillin/streptomycin (Gibco) and 4 mM L-glutamine (Sigma) as previously described.[19] Both adherent and suspension 3LL cells were used for passaging and expansion. For lung tumor implantation, adherent 3LL cells were harvested by trypsinization (Gibco), resuspended in PBS and $1x10^6$ tumor cells were injected intravenously into the bone marrow chimeras 17 weeks after bone marrow transplant. Lung tumor growth was monitored by computed tomography (CT) scanning twice per week, wherein mice were anesthetized by inhalation of isoflurane and scanned using the Quantum GX2 micro-CT imaging system (PerkinElmer) at a 50-µm isotropic pixel size.[20] Mice were sacrificed when the largest tumor reached 6 mm in diameter, approximately 3-5 weeks after tumor implantation.

1.6.3. Blood and tissue harvest and processing

Peripheral blood sampling was performed twice before tumor induction (6 and 16 weeks after bone marrow transplant) and once at the 3 weeks time point after tumor induction (20 weeks after bone marrow transplant) via tail vein bleeds. For endpoint tissue harvest,

mice were sacrificed by overdose of pentobarbital, followed by collection of cardiac blood, lung perfusion with 20 mL of PBS and harvest of tumor-bearing lungs. Tumor-bearing lungs were further dissected to separate tumor nodules and adjacent lung tissue without visible lesions. Tail vein and cardiac blood was collected in heparin-coated tubes and 3-4 rounds of red blood cell lysis were performed using ACK lysing buffer (Gibco) following manufacturer's instructions, until the cell pellets were no longer red. While blood cells were then washed twice with FACS buffer and stained for flow cytometry analysis or used for the *ex vivo* migration assay, as described below. Lung tumors and adjacent lung tissues were dissected, minced, then incubated in 250 mg/mL Liberase (Sigma-Aldrich) and 50 mg/mL DNase (Sigma-Aldrich) in RPMI (Gibco) for 30 min at 37°C and mashed through a 70 μm filter to generate a single cell suspension. Cells were then washed with FACS buffer, pelleted by centrifugation at 500 g for 3 min at 4°C and stained for flow cytometry analysis.

1.6.4. Flow cytometry analysis

Cells were resuspended in FACS buffer containing Fixable Viability Dye eFluor 780, (eBioscience #65-0865-14, 1:1000) to distinguish live and dead cells and aCD16/CD32 (clone 93, BioLegend #101302, 1:100) to prevent non-specific antibody binding, and incubated for 15 min at 4°C. Cells were then washed with FACS buffer and stained for surface proteins for 20 min at 4°C using fluorophore-conjugated antibodies including CD3-BUV395 (clone 17A2, BD #740268), CD45.2-BUV563 (clone 104, BD #741273), CD8a-BUV737 (clone 53-6.7, BD #612759), CD11c-PacBlue (clone N418, Biolegend #117322), NK1.1-BV421 (clone PK136, Biolegend #108741), CD103-BV510 (clone 2E7, Biolegend #121423), Ly6C-BV570 (clone HK1.4, Biolegend #128030), CD115-BV605 (clone AFS98, Biolegend #135517), CD4-BV650 (clone RM4-5, Biolegend #100555), CD11b-BV711 (clone M1/70, Biolegend #101242), CD19-BV785 (clone 6D5, Biolegend #115543), MerTK-PE (clone 2B10C42, Biolegend #151506), SiglecF-PE-CF594 (clone E50-2440, BD #562757), MHCII-PE-Cy5 (clone M5/114.15.2, Biolegend #107612), CD64-PE-Cy7 (clone X54-5/7.1, Biolegend #139314), CD45.1-APC (clone A20, eBioscience #17045382), Ly6G-AF700 (clone 1A8, Biolegend #127622) resuspended in 1:5 mix of Brilliant Stain Buffer (BD) and FACS buffer. After surface staining, cells were washed twice with FACS buffer and analyzed directly or fixed for downstream intracellular staining and/or analysis the next day. Cell fixation was performed using the Foxp3 Transcription Factor Fixation/Permeabilization buffer (eBioscience) when staining for transcription factors, or using the Cytofix/Cytoperm Fixation/Permeabilization buffer (BD Biosciences), following manufacturer's instructions. After fixation, cells were washed twice with FACS buffer, and if required, further stained for intracellular proteins in FACS buffer overnight at 4°C and then washed twice with FACS buffer prior to flow analysis. To obtain absolute counts of cells, Precision Count Beads (BioLegend) were added to samples following manufacturer's instructions. Flow cytometry sample acquisition was performed on the

ID7000 Spectral Analyzer (Sony Biotechnology), and data was analyzed using FlowJo v10.5.3 software (TreeStar).

1.6.5. *Ex vivo* migration assay

The *ex vivo* migration assay was adapted from Mugarza et al.[20] $1x10^5$ 3LL LUAD cells (including adherent and suspension cells) were seeded in 600 µL of 3LL culture medium per well in the bottom chamber of a 24-well transwell plate with 5.0 µm pore polycarbonate membrane insert (Corning). The next day, peripheral blood was harvested via tail bleeds from bone marrow chimeric mice with $Tet2^{mut}$ CHIP (at the 3 week time point after 3LL lung tumor induction). Blood processing and red blood cell lysis were performed as described above. White blood cells were counted and pooled as needed to plate $1x10^5$ cells in 100 µL per well in the top chamber i.e. transwell insert. Immune-tumor cell transwell co-cultures were set up alongside immune-media only transwell cultures to control for the effect of 3LL tumor cells on immune cell migration. After 2 hours of co-culture, cells from top and bottom chambers were harvested and stained for flow cytometry analysis. Precision count beads were added to quantify absolute numbers of cells in each well. Percent migrated $Tet2^{mut}$ monocytes was calculated as the absolute number of $CD45.2^+$ monocytes in the bottom chamber divided by the sum of the absolute numbers of $CD45.2^+$ monocytes in top and bottom chambers, multiplied by 100. For wild-type monocytes, the same calculation was performed using the combined $CD45.1^+$ and $CD45.1^+$ $CD45.2^+$ congenic fractions.

1.7. Co-culture of patient-derived tumor organoids with *TET2*^{mut} myeloid cells

1.7.1. Lung tumor organoid establishment and passaging

Organoids were derived from a previously described patient-derived xenograft (PDX) established from patient CRUK0718 (tumor region 7).[21] We used published methods with minor adaptations for organoid establishment and culture.[22–24] Briefly, a tumor single-cell suspension was generated from the PDX tumor (passage 2) using a gentleMACS tumor dissociation kit (Miltenyi Biotec). Single cells were seeded as organoids as previously described,[22,24] and cultured in minimal basic medium.[23] Organoids were expanded, passaged, and cryopreserve.[22,24] Organoids were authenticated using short tandem repeat (STR) profiling and regularly tested for Mycoplasma contamination.

1.7.2. Humanized mouse model of *TET2*^{mut} CHIP

The humanized mouse model of *TET2*-derived clonal hematopoiesis was generated as previously described.[25] Briefly, umbilical cord blood was processed to isolate human hematopoietic stem cells and *TET2* loss-of-function mutations were introduced by

CRISPR-editing. Umbilical Cord Blood was obtained from full-term donors after informed consent at the Royal London Hospital (London, U.K.) and under approval by the East London Ethical Research committee. Mononuclear cells were isolated by density centrifugation using Ficoll-Paque (GE 67 Healthcare) and depleted for lineage$^+$ cells using an EasySep Human Progenitor Cell Enrichment Kit (Stem Cell Technologies). Hematopoietic stem and progenitor cells (HSPC) were isolated using FACS as previously described.[26] Lineage$^-$CD34$^+$CD38$^-$ cells were cultured in StemSpanSFEM medium (Stem Cell Technologies) with 100 ng/mL rhFLT-3L, 100 ng/mL rhSCF, and 100 ng/mL rhTPO for 48 hours. CRISPR editing was then performed with the NEON Transfection system (Thermo Fisher) to introduce the ribonucleoprotein complex using the small guide RNAs and donor templates previously described.[25] These animal experiments were performed under the U.K Home Office project license (70/8904) in accordance with The Francis Crick Institute animal ethics committee guidance and bred in isolators with aseptic standard operating procedures in the Biological Research Facility of The Francis Crick Institute. NBSGW (NOD/SCID/IL2rγ$^{-/-}$/Tyr$^+$/Kit^{W41J}) mice between 8-12 weeks of age were injected with human hematopoietic stem cells (10,000-20,000 Lin$^-$CD34$^+$CD38$^-$ cells/mouse) via intravenous administration. Engraftment of the reconstituted human hematopoietic system was validated for each mouse by bone marrow aspiration at 6 weeks.

1.7.3. Isolation of human lung myeloid cells

Human myeloid cells were isolated from humanized *TET2*^{mut} or wild-type control HSPC immunodeficient mice 12 weeks after human bone marrow reconstitution. Humanized mouse lungs were harvested, minced and digested with DNAse (0.2 mg/ml) and Collagenase IV (4000 U/ml) for 20 min at 37°C and filtered through a 100 μm strainer. Cells were then prepared for fluorescence-activated cell sorting (FACS) by incubating for 15 minutes with human TruStain FcX (Biolegend, cat#422302), followed by staining with the flow antibody panel: mCD45-BV421 (clone 30-F11, BD Horizon), hCD45-APC/Cy7 (clone HI30, Biolegend) and CD33-PE (clone P67.6, Biolegend). Dead cells were excluded by staining with the fluorescent dye DAPI (1 μg/ml; BD Biosciences, cat# 564907) and gating out the positive cells. For sorting, cell suspensions were filtered through a 35-μm nylon mesh (Falcon, cat# 352235) and sorted using a BD FACS FUSION cell sorter equipped with 488-nm, 561-nm, 633-nm, and 405-nm lasers. All experiments were analyzed with FACSDiva 6.2 (BD Biosciences).

1.7.4. Tumor:myeloid cell co-culture

Lung tumor organoid cells were dissociated into single cells using accutase (Gibco) and mixed with human lung myeloid cells at a 1:1 ratio (each cell population was prepared at a concentration of 1.5×10^6 cells/mL prior to mixing) in co-culture media: DMEM/F12 (Gibco) supplemented with 100/100 U/ml Penicillin/Streptomycin, 1% human serum (Sigma) and

B27 (Gibco). Two parts volume of Geltrex LDEV-free reduced growth factor basement membrane extract (Gibco) was added to the cell suspension and 20µL cell-Geltrex drops were plated in 48-well plate (1 drop per well, each drop containing 5000 tumor cells and 5000 myeloid cells). Plates were left to solidify upside-down in the 37°C incubator for 30 min, after which cells were overlaid with co-culture media (200µL/well). Media was refreshed one week later. Cells were cultured for a total of two weeks, at which point the organoids were imaged to quantify organoid number and size using the Fiji software.[27]

2. Supplementary Figures

Figure S1. CHIP in patients with treatment-naïve early-stage NSCLC before surgery enrolled in TRACERx.

A. Schematic of the TRACERx non-small cell lung cancer (NSCLC) cohort and the different data modalities analyzed.

B. Association between age and the prevalence of CHIP in TRACERx. R^2 and p-value are from pearson correlation.

C. Prevalence of CHIP in TRACERx and number of patients with 1, 2, or more than 2 CHIP mutations.

D. Density distribution of the variant allele frequency (VAF) values of the 179 CHIP mutations identified in TRACERx. The red dotted line represents the median value (5%).

E. Frequency distribution of the top mutated genes in CHIP in TRACERx color-coded by the level of variant allele frequency (VAF) of the mutations.

F. Heatmap representing the co-mutation patterns of CHIP mutations in TRACERx. Mutations are color-coded by the level of VAF. Each column represents one of the 143 patient with CHIP.

G. Multivariable logistic regression for the presence of CHIP in TRACERx. Explanatory covariates include demographic variables (age, sex), smoking status, and histology. Age (in years) was used as a continuous variable, and divided by 5. LUAD: lung adenocarcinoma, LUSC: lung squamous carcinoma.

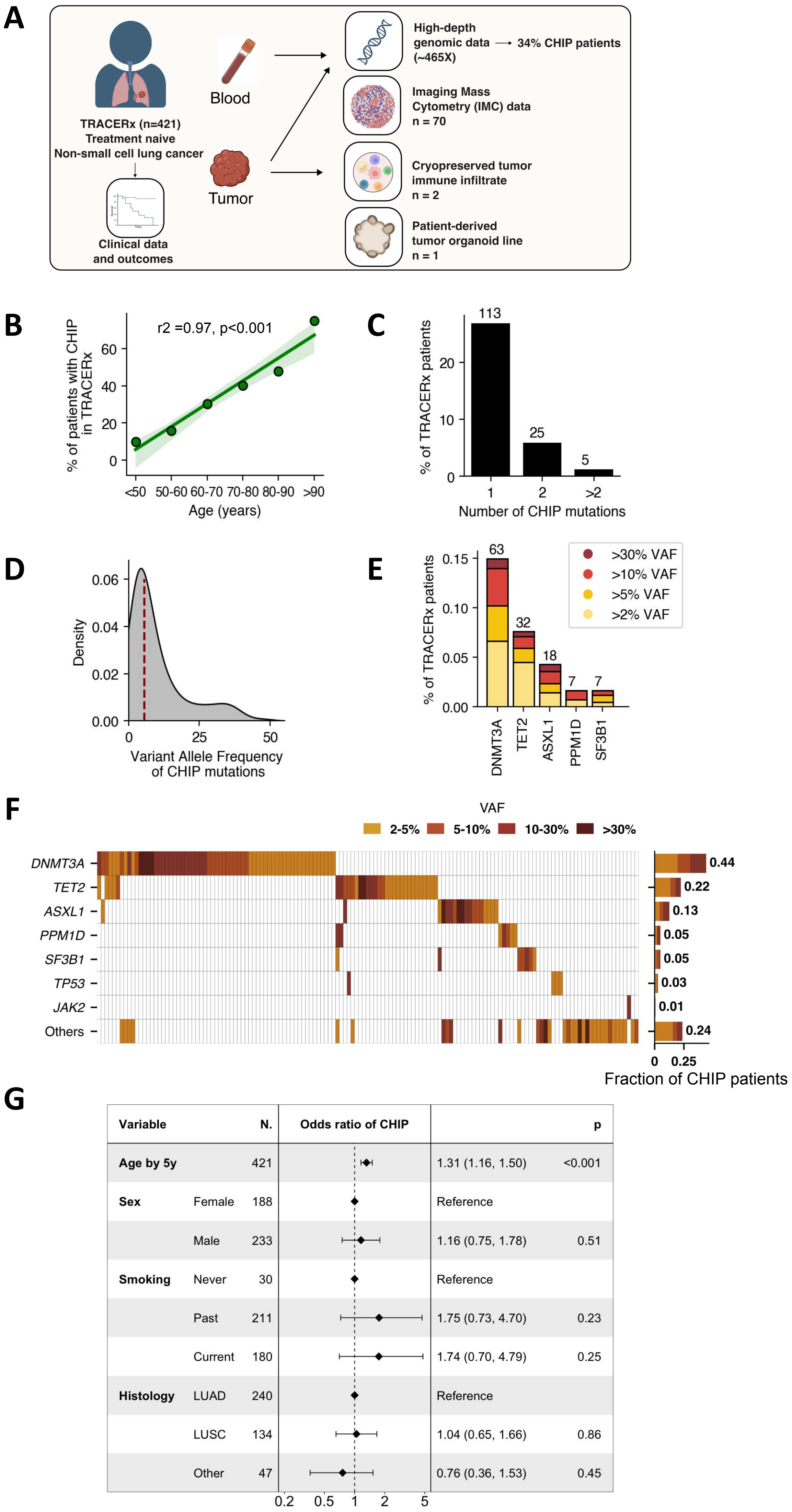
A
TRACERx (n=421)
Treatment naive
Non-small cell lung cancer
Clinical data
and outcomes
Blood
Tumor
High-depth
genomic data → 34% CHIP patients
(~465X)
Imaging Mass
Cytometry (IMC) data
n = 70
Cryopreserved tumor
immune infiltrate
n = 2
Patient-derived
tumor organoid line
n = 1
B
r2 =0.97, p<0.001
% of patients with CHIP in TRACERx
Age (years)
<50 50-60 60-70 70-80 80-90 >90
C
% of TRACERx patients
Number of CHIP mutations
113
25
5
D
Density
Variant Allele Frequency
of CHIP mutations
E
% of TRACERx patients
>30% VAF
>10% VAF
>5% VAF
>2% VAF
DNMT3A
TET2
ASXL1
PPM1D
SF3B1
63
32
18
7
7
F
VAF
2-5%
5-10%
10-30%
>30%
DNMT3A
TET2
ASXL1
PPM1D
SF3B1
TP53
JAK2
Others
0.44
0.22
0.13
0.05
0.05
0.03
0.01
0.24
Fraction of CHIP patients
G
Variable
N.
Odds ratio of CHIP
p
Age by 5y 421 1.31 (1.16, 1.50) <0.001
Sex Female 188 Reference
Male 233 1.16 (0.75, 1.78) 0.51
Smoking Never 30 Reference
Past 211 1.75 (0.73, 4.70) 0.23
Current 180 1.74 (0.70, 4.79) 0.25
Histology LUAD 240 Reference
LUSC 134 1.04 (0.65, 1.66) 0.86
Other 47 0.76 (0.36, 1.53) 0.45

Figure S2. CHIP in patients with LUAD or LUSC in the TRACERx cohort.

A. Proportion of patients with CHIP in the lung adenocarcinoma (LUAD, n=240 total patients) or lung squamous carcinoma (LUSC, n=134 total patients) subsets of the TRACERx cohort.

B. Density distribution of the variant allele frequency (VAF) values of the CHIP mutations in the LUAD (n=104 mutations) and LUSC (n=59 mutations) subsets.

C. Comparison of the frequency of mutated genes across patients with CHIP in the LUAD (n=80 CHIP patients) and LUSC (n=50 CHIP patients) subsets.

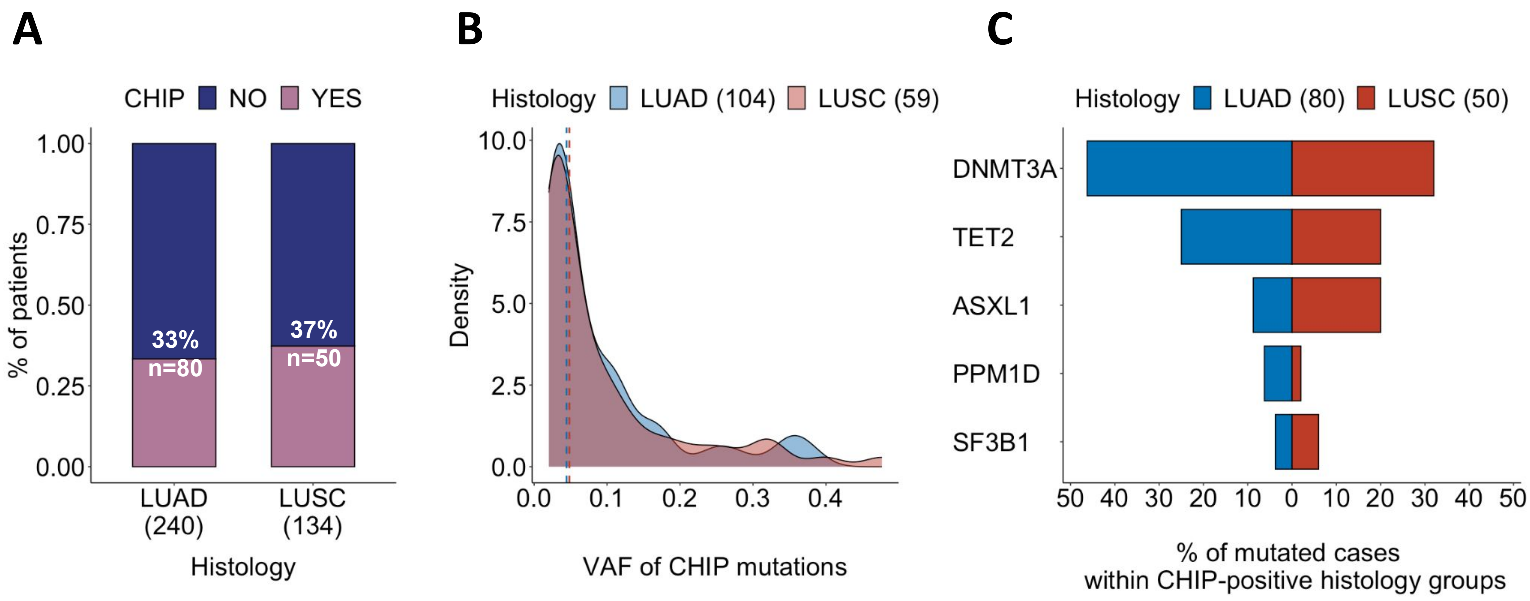

Figure S3. Comparison of the distribution of tumor driver alterations between patients with or without CHIP in TRACERx.

Frequency of mutated genes within the group of patients with (pink) or without (blue) CHIP in the whole TRACERx cohort (**A**) or in the LUAD subset (**B**). The distribution of oncogenic isoforms affecting *MET*, *ALK*, *RET*, and *ROS1* is provided in a separate representation. No significant differences were observed in the distribution of mutations between the two groups. LUAD: lung adenocarcinoma.

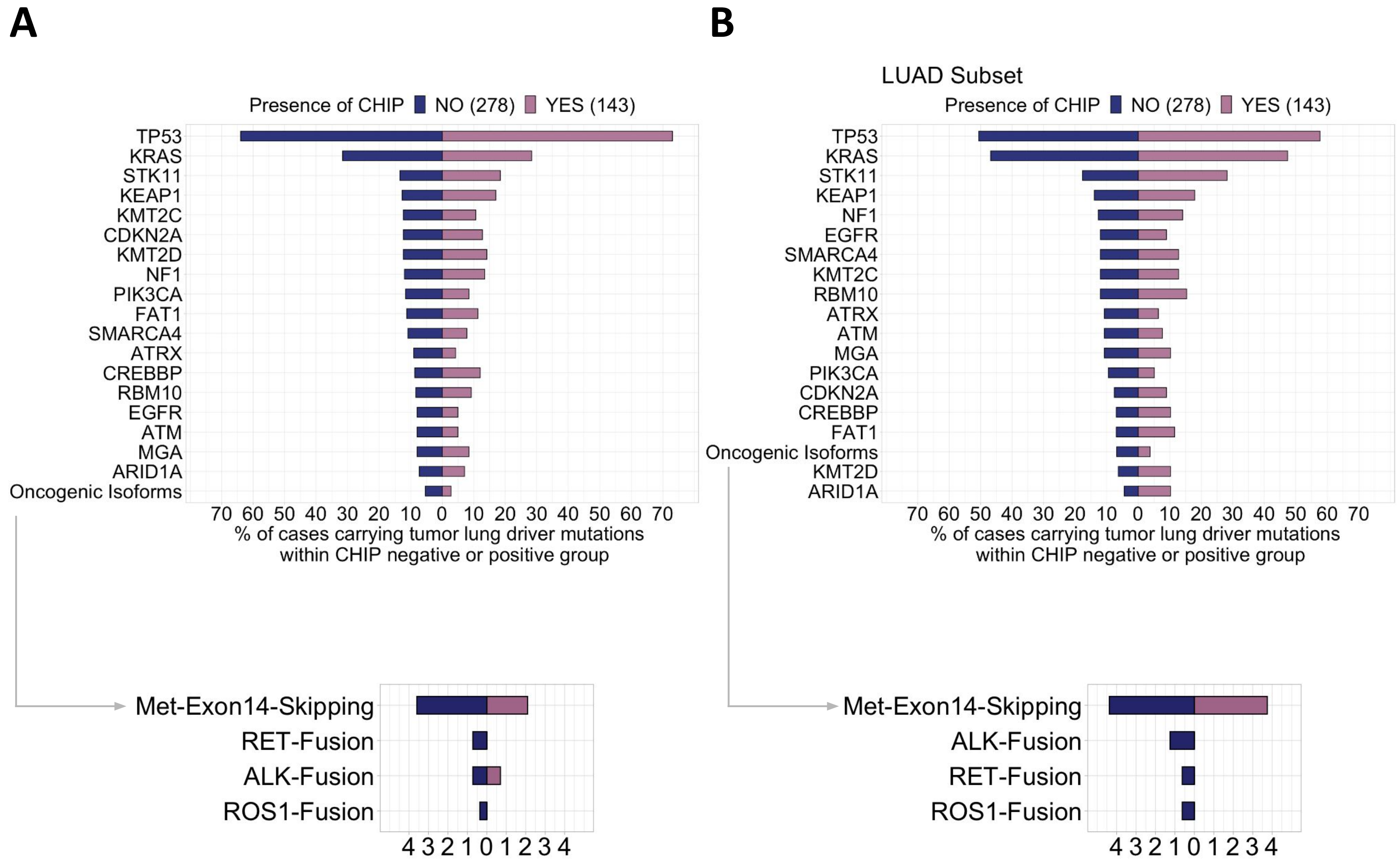

Figure S4. Association between CHIP and overall survival in TRACERx.

Kaplan-Meier probability estimates of overall survival across patients without (blue) or with (purple) CHIP in TRACERx. P-value is from the log-rank test.

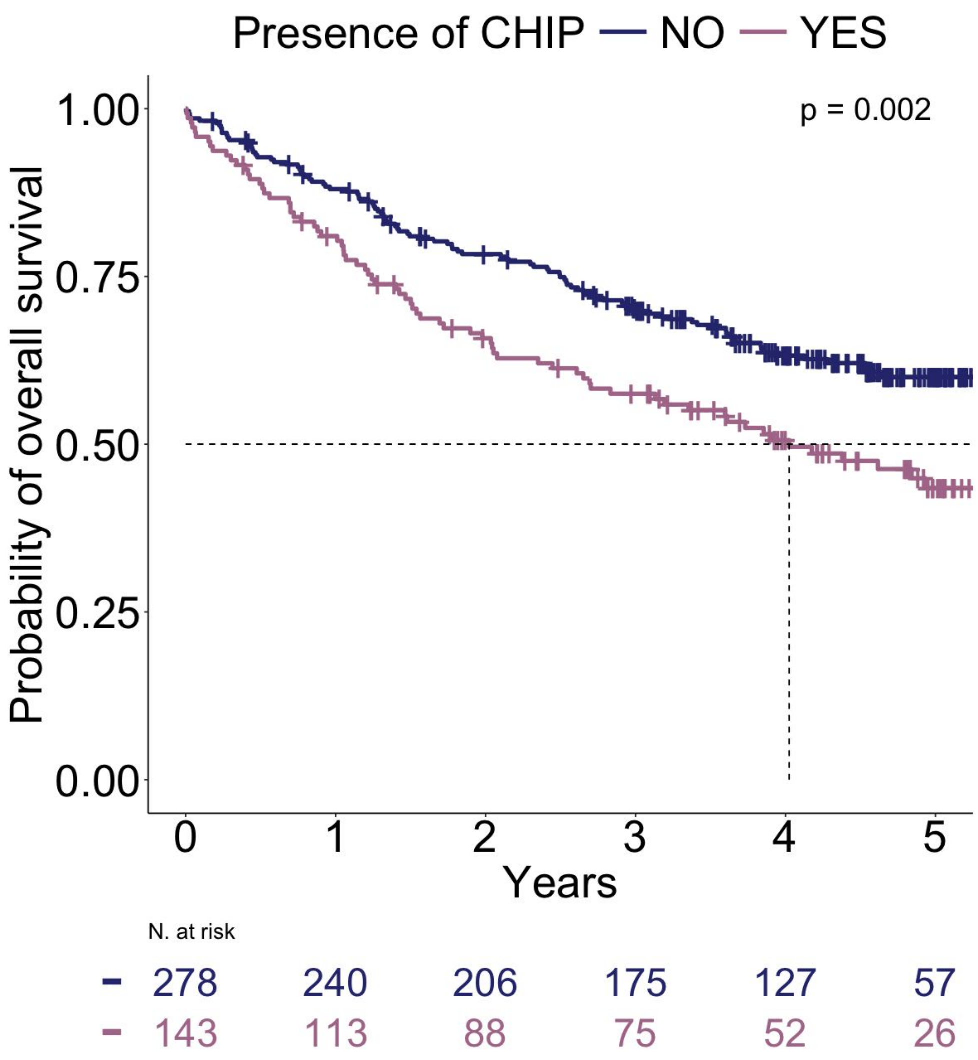

Figure S5. Incorporation of lung tumor driver alterations in multivariable analysis for the association between CHIP and patient outcomes in TRACERx, including the risk of all-cause mortality (**A**) and the risk of tumor recurrence or death (**B**). The models account for demographics (age, sex), adjuvant treatment, smoking status, tumor stage, and histology, as well as the main lung tumor driver mutations (*TP53*, *KRAS*, *STK11*, and *EGFR* mutations) and oncogenic isoforms (*MET* exon 14 skipping, and *RET*, *ALK*, and *ROS1* fusions). Age in years was used as a continuous variable, and divided by 5 so that the hazard ratio represents the change in risk for an increase of 5 years.

A Risk of all-cause mortality

Variable		N	Hazard ratio		p
**Presence of CHIP**	NO	278		Reference	
	YES	143		1.65 (1.21, 2.26)	0.002
**Age by 5y**		421		1.13 (1.02, 1.26)	0.02
**Sex**	Female	188		Reference	
	Male	233		1.05 (0.76, 1.45)	0.76
**Adjuvant treatment**	No	287		Reference	
	Yes	134		0.62 (0.42, 0.90)	0.01
**Smoking**	Current	180		Reference	
	Former	211		0.96 (0.68, 1.35)	0.82
	Never	30		0.44 (0.18, 1.06)	0.07
**Stage**	I	210		Reference	
	II	132		2.76 (1.85, 4.11)	<0.001
	III	79		5.51 (3.58, 8.46)	<0.001
**Histology**	LUAD	240		Reference	
	LUSC	134		1.64 (1.07, 2.50)	0.02
	Other	47		2.81 (1.73, 4.56)	<0.001
**TP53 lung driver mutation**	Wild type	142		Reference	
	Mutant	279		0.83 (0.58, 1.20)	0.32
**KRAS lung driver mutation**	Wild type	294		Reference	
	Mutant	127		1.44 (0.95, 2.19)	0.08
**STK11 lung driver mutation**	Wild type	358		Reference	
	Mutant	63		1.00 (0.64, 1.56)	1
**EGFR lung driver mutation**	Wild type	392		Reference	
	Mutant	29		0.65 (0.27, 1.54)	0.33
**Oncogenic Isoforms**	Absence	402		Reference	
	Presence	19		1.34 (0.63, 2.82)	0.45

B Risk of tumor recurrence or death

Variable		N	Hazard ratio		p
**Presence of CHIP**	NO	278		Reference	
	YES	143		1.50 (1.12, 2.01)	0.007
**Age by 5y**		421		1.12 (1.02, 1.24)	0.02
**Sex**	Female	188		Reference	
	Male	233		1.14 (0.85, 1.53)	0.38
**Adjuvant treatment**	No	287		Reference	
	Yes	134		0.81 (0.57, 1.15)	0.25
**Smoking**	Current	180		Reference	
	Former	211		0.88 (0.65, 1.20)	0.43
	Never	30		0.41 (0.19, 0.89)	0.02
**Stage**	I	210		Reference	
	II	132		2.30 (1.58, 3.35)	<0.001
	III	79		4.65 (3.13, 6.91)	<0.001
**Histology**	LUAD	240		Reference	
	LUSC	134		1.48 (1.00, 2.18)	0.05
	Other	47		2.30 (1.47, 3.62)	<0.001
**TP53 lung driver mutation**	Wild type	142		Reference	
	Mutant	279		0.79 (0.57, 1.10)	0.17
**KRAS lung driver mutation**	Wild type	294		Reference	
	Mutant	127		1.36 (0.93, 1.99)	0.12
**STK11 lung driver mutation**	Wild type	358		Reference	
	Mutant	63		1.03 (0.68, 1.55)	0.89
**EGFR lung driver mutation**	Wild type	392		Reference	
	Mutant	29		0.91 (0.44, 1.84)	0.78
**Oncogenic Isoforms**	Absence	402		Reference	
	Presence	19		1.42 (0.72, 2.78)	0.31

Figure S6. Association between CHIP and tumor microenvironment in TRACERx.

A. Representative example of spatial immunophenotyping performed by multiplex imaging mass cytometry (IMC) in TRACERx. Colors represent cell types.

B. Age-adjusted logistic regression for the association between the presence of CHIP and cellular densities (higher vs. lower than the cohort average for each cell type) in the lung tumor microenvironment. 117 tumors from 49 CHIP-negative patients and 46 tumors from 21 CHIP-positive patients were included. I: confidence interval. ***p<0.001; **p<0.01. P-values were adjusted using the Benjamini-Hochberg method.

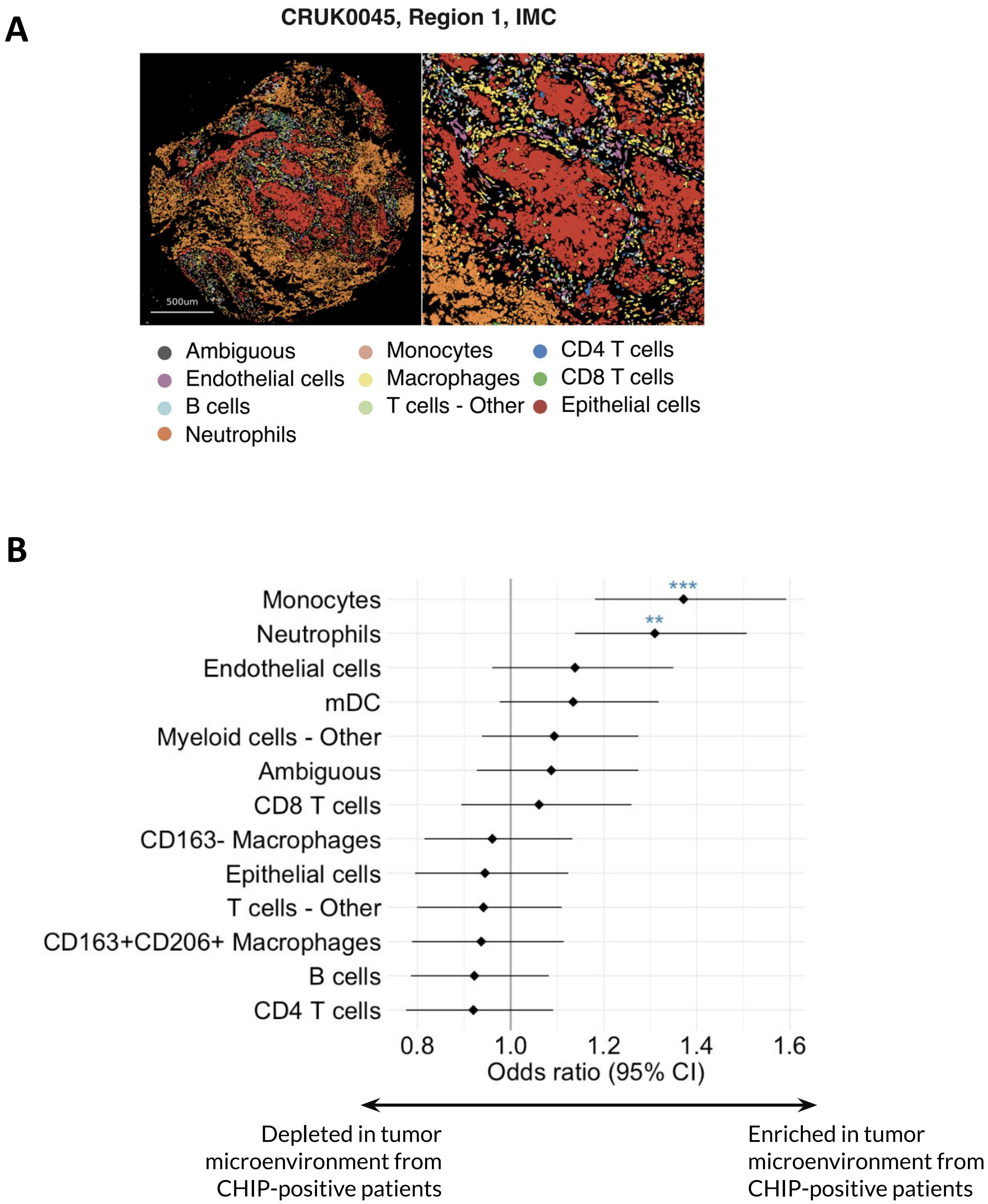

Figure S7. Methodology for TI-CH detection and specificity analysis.

A. Schematic depicting the genotyping of patient-specific CHIP mutations in the matched tumor samples.

B. Sequencing coverage distribution in tumor samples across patients without CHIP, with blood-only CHIP, or with TI-CH.

C. Schematic and results of the specificity analysis. Top frequent mutations in CHIP were genotyped in both blood and tumors samples from TRACERx whole exome sequencing data. We quantified how many times each mutation was observed with at least two supporting reads in tumor samples when it was not observed in the blood. The analyses revealed that <1% of the examined tumor-mutation pairs had the mutation in the tumor but not in the blood.

A

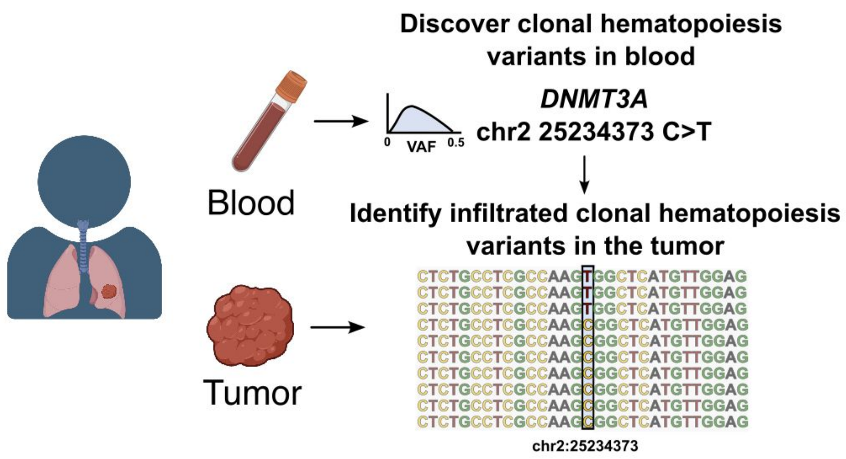

B

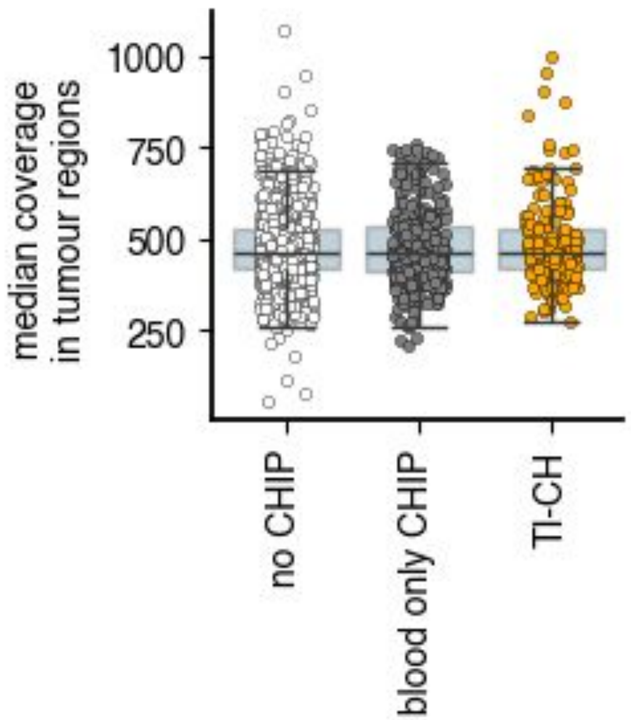

C

n=52 top frequent CHIP mutations

Select tumor samples from patients without the CHIP mutations in blood

<u>Tumor from patients without each CHIP mutation in matched blood sample</u>

n=74920 total tumor sample-mutation pairs
n=465 tumor samples with mutation identified with >1 supporting reads

specificity = 1 - (465/74920) = 99.3%

Figure S8. Variant allele fraction of CHIP mutations in blood and matched tumors and ddPCR validation.

A. Correlation between VAF of CHIP mutations in the blood and the VAF of the same mutations in the matched tumors. Colors represent the mutated genes. The VAF of CHIP mutations in tumors was consistently lower than the VAF in blood, except for one case with *JAK2* V617F hotspot mutation (VAF of 26% in tumors vs. 19% in blood, red color) where pathology assessment reported a high level of tumor-infiltrating neutrophils.

B. Comparison between the VAF of CHIP mutations in the blood (triangle) or tumor (star) samples assessed by NGS sequencing (y-axis) or digital droplet based PCR (x-axis).

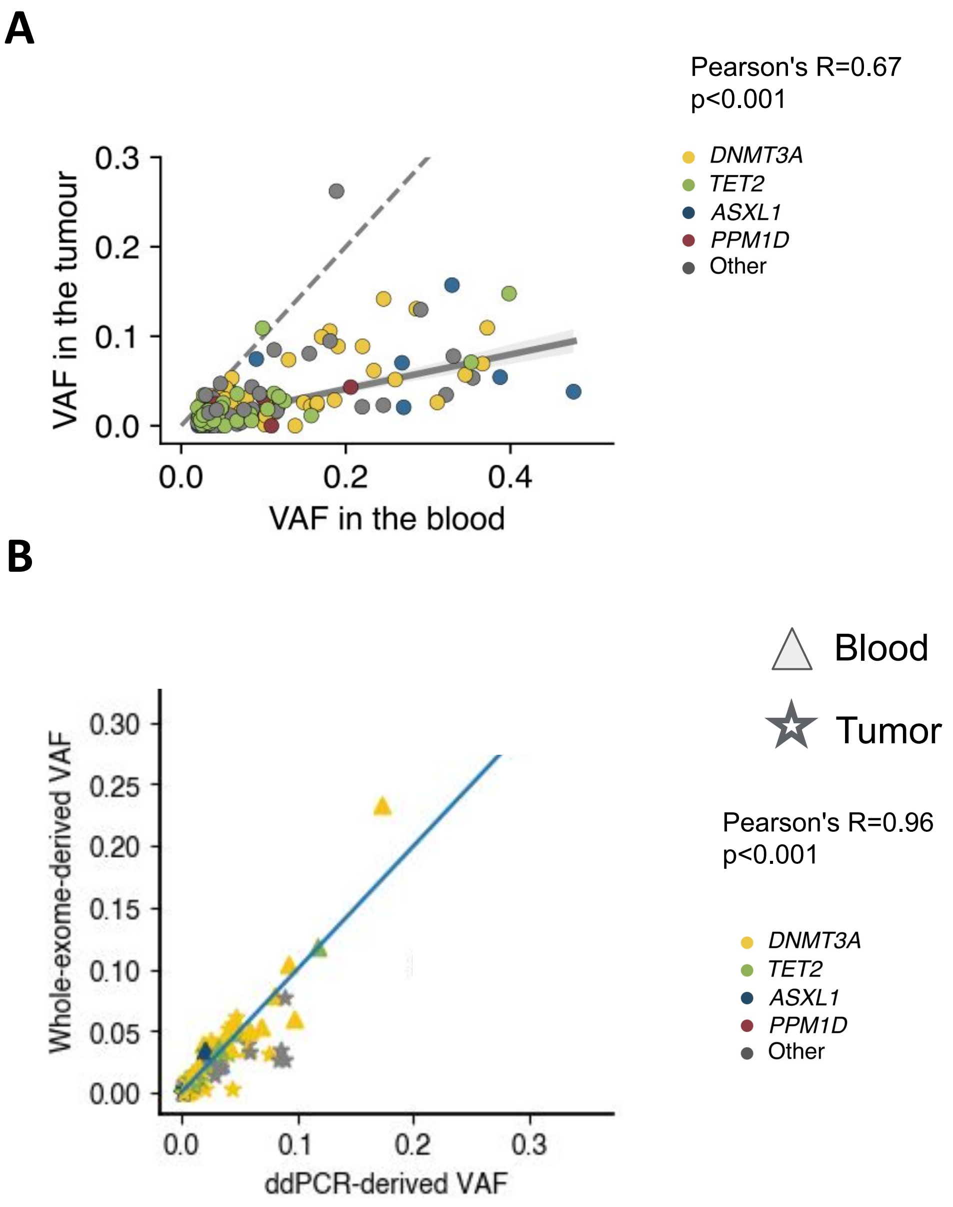

Figure S9. A. Variant allele frequency (VAF) of CHIP mutations across 226 tumor regions from 60 TRACERx patients with TI-CH. Each patient is depicted with a pie chart, where the number of slices corresponds to the number of tumor regions analyzed. The colors of each slice indicate the level of VAF, and the exact VAF values are provided in adjacent boxes. The patient ID and the mutated gene are indicated on top of each pie chart. **B.** Classification of the 60 TRACERx patients with TI-CH according to the fraction of tumor regions that were positive for TI-CH (i.e. VAF in tumor above 2%).

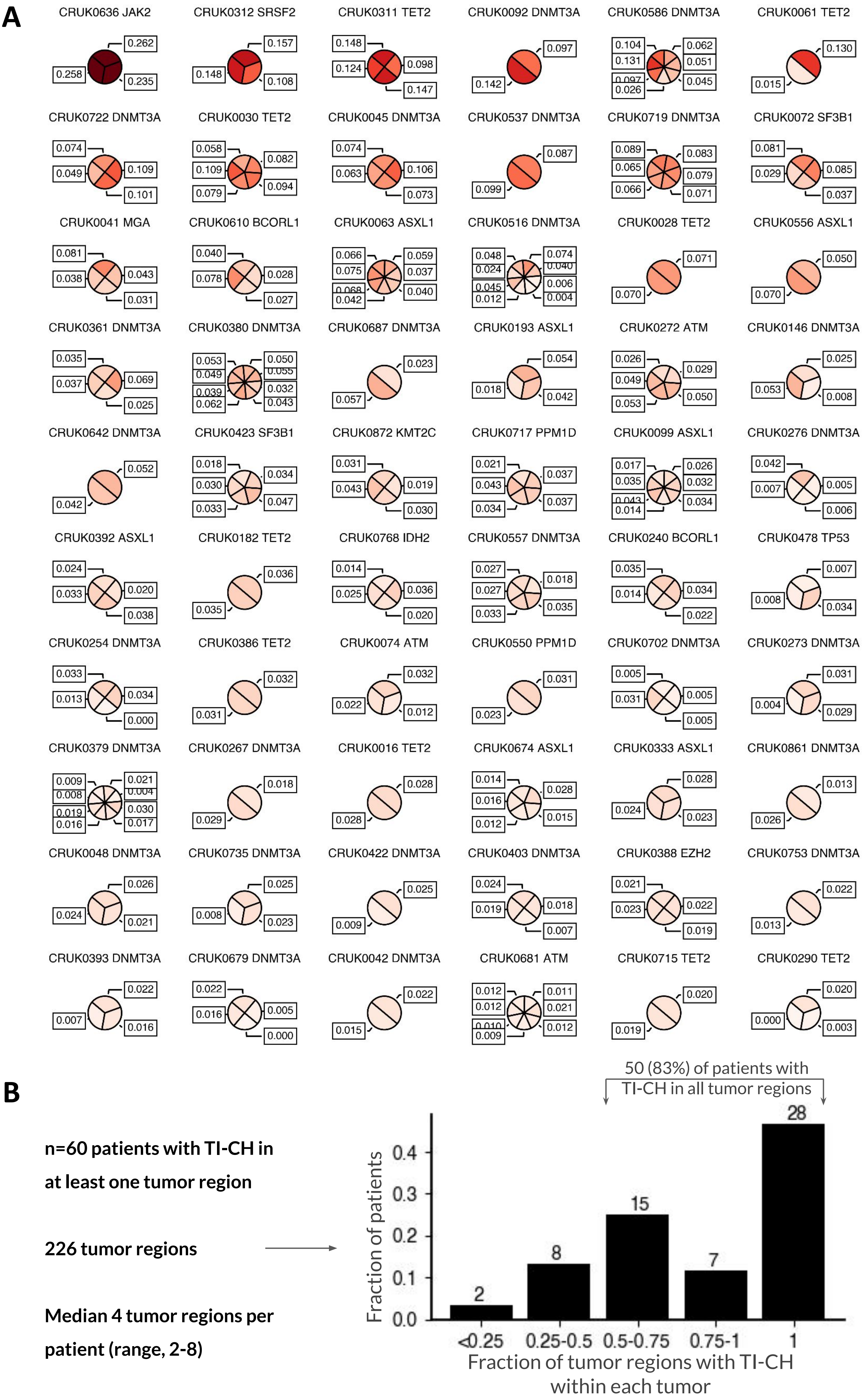
A
CRUK0636 JAK2
CRUK0312 SRSF2
CRUK0311 TET2
CRUK0092 DNMT3A
CRUK0586 DNMT3A
CRUK0061 TET2
CRUK0722 DNMT3A
CRUK0030 TET2
CRUK0045 DNMT3A
CRUK0537 DNMT3A
CRUK0719 DNMT3A
CRUK0072 SF3B1
CRUK0041 MGA
CRUK0610 BCORL1
CRUK0063 ASXL1
CRUK0516 DNMT3A
CRUK0028 TET2
CRUK0556 ASXL1
CRUK0361 DNMT3A
CRUK0380 DNMT3A
CRUK0687 DNMT3A
CRUK0193 ASXL1
CRUK0272 ATM
CRUK0146 DNMT3A
CRUK0642 DNMT3A
CRUK0423 SF3B1
CRUK0872 KMT2C
CRUK0717 PPM1D
CRUK0099 ASXL1
CRUK0276 DNMT3A
CRUK0392 ASXL1
CRUK0182 TET2
CRUK0768 IDH2
CRUK0557 DNMT3A
CRUK0240 BCORL1
CRUK0478 TP53
CRUK0254 DNMT3A
CRUK0386 TET2
CRUK0074 ATM
CRUK0550 PPM1D
CRUK0702 DNMT3A
CRUK0273 DNMT3A
CRUK0379 DNMT3A
CRUK0267 DNMT3A
CRUK0016 TET2
CRUK0674 ASXL1
CRUK0333 ASXL1
CRUK0861 DNMT3A
CRUK0048 DNMT3A
CRUK0735 DNMT3A
CRUK0422 DNMT3A
CRUK0403 DNMT3A
CRUK0388 EZH2
CRUK0753 DNMT3A
CRUK0393 DNMT3A
CRUK0679 DNMT3A
CRUK0042 DNMT3A
CRUK0681 ATM
CRUK0715 TET2
CRUK0290 TET2
B
n=60 patients with TI-CH in at least one tumor region
226 tumor regions
Median 4 tumor regions per patient (range, 2-8)
50 (83%) of patients with TI-CH in all tumor regions
Fraction of patients
0.0
0.1
0.2
0.3
0.4
2
8
15
7
28
<0.25
0.25-0.5
0.5-0.75
0.75-1
1
Fraction of tumor regions with TI-CH within each tumor

Figure S10. Variant allele fraction (VAF) of CHIP mutations in the blood (blue) and in tumor regions from two TRACERx patients with synchronous primary tumors (**A**) and two TRACERx patients with metachronous primary tumors (**B**). The patient ID and the mutated gene are indicated on top of each graph. Synchronous primary tumors: multiple primary lung tumors with distinct genomic origins diagnosed simultaneously. Metachronous tumors: multiple primary tumors with distinct genomic origins diagnosed at different times.

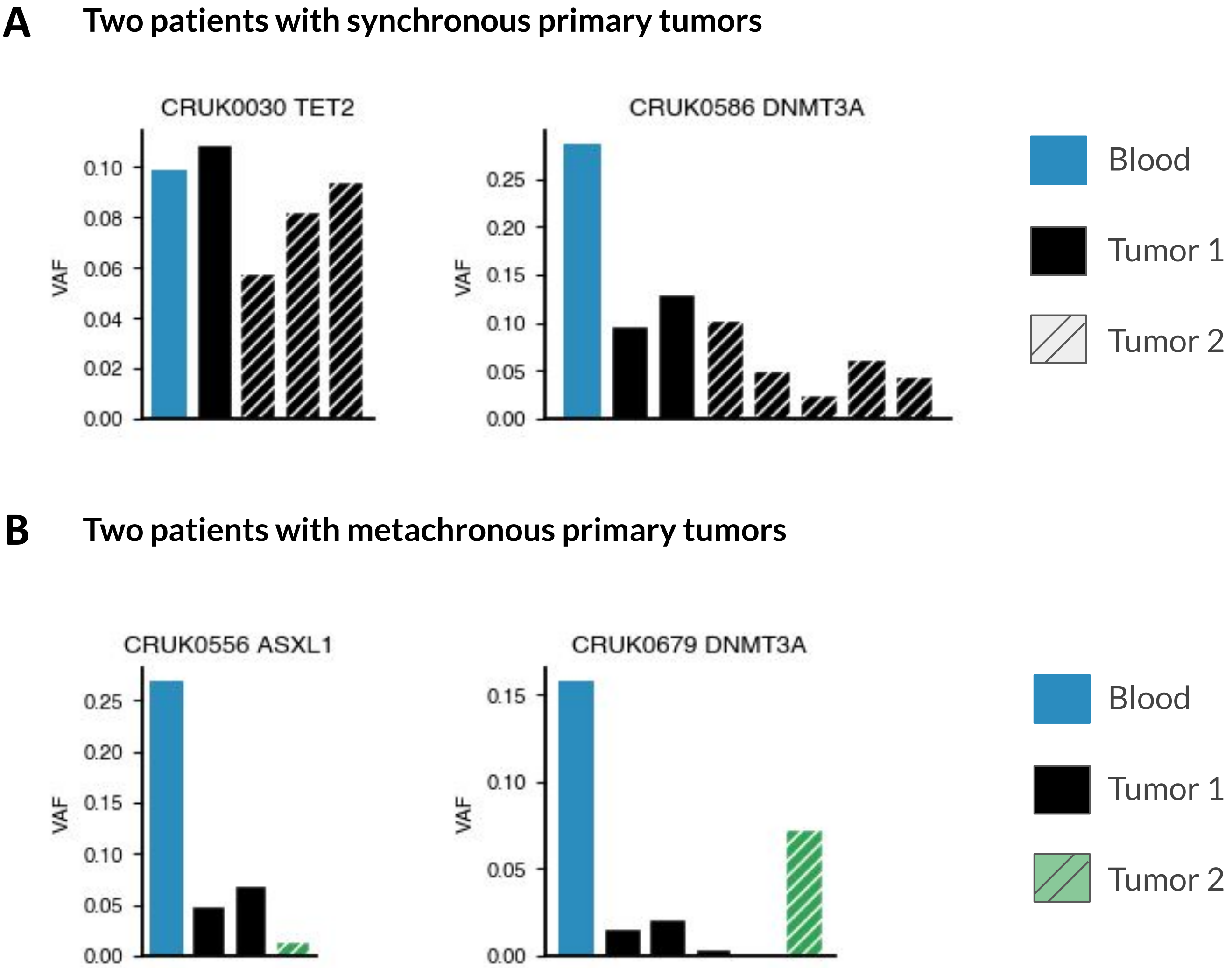

Figure S11. Gene-specific TI-CH in the NSCLC validation cohort.

A. Mutated gene frequency distribution in CHIP (n=917) and TI-CH (n=333) in the validation cohort of patients with stage I-III NSCLC.

B. Frequency of TI-CH within patients with gene-specific CHIP. For example, within 467 patients with *DNMT3A*-mutant CHIP, 161 (34%) had TI-CH.

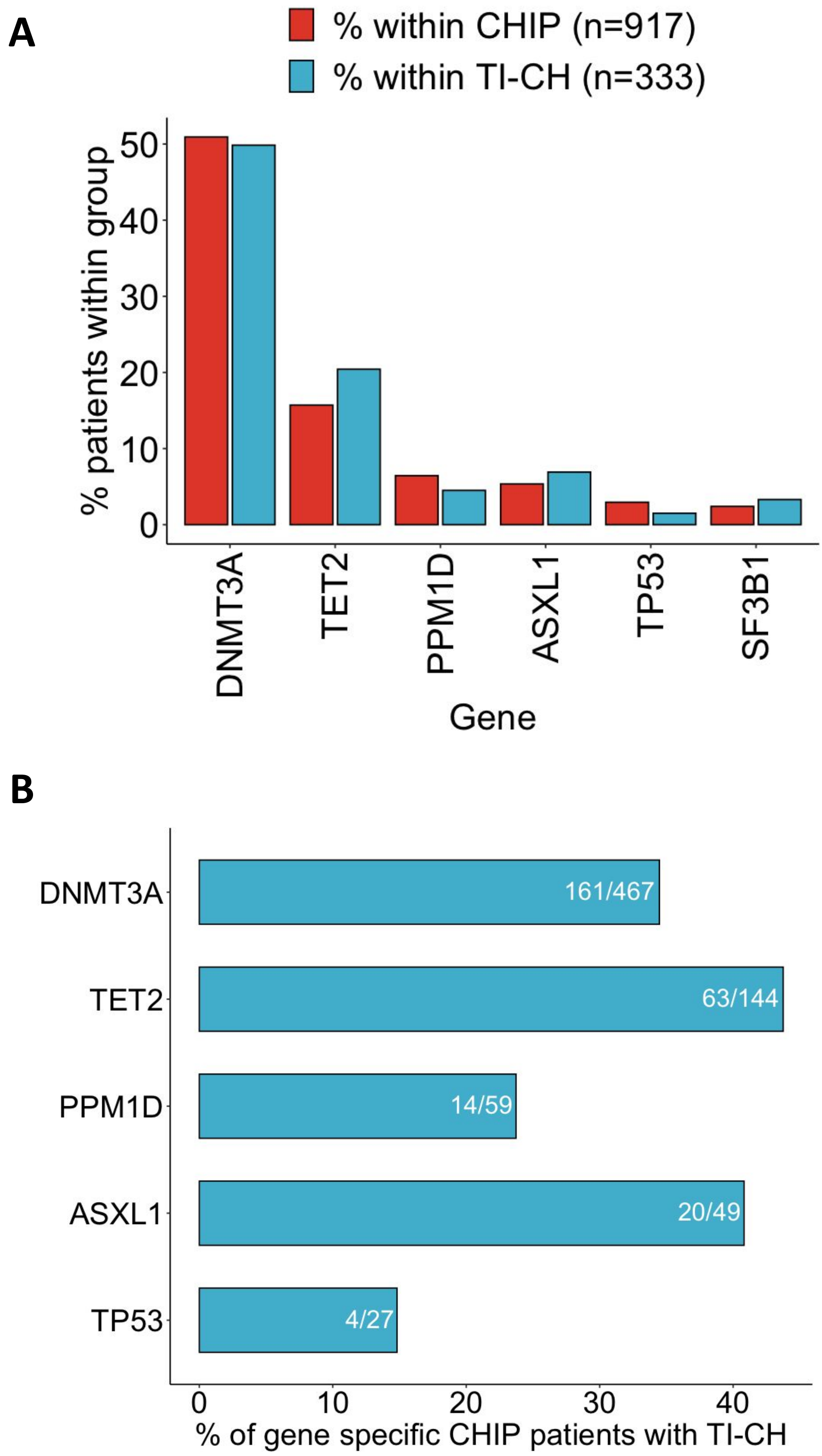

Figure S12. Validation of CHIP-mutant immune cell infiltration in NSCLC.

A. Schematic of the single-cell genotyping experiment focused on immune cell populations isolated from a primary lung tumor of a patient with *TET2*- and *SRSF2*-mutant TI-CH.

B. Flow cytometry gating strategy for human tumor-infiltrating immune cells and peripheral blood mononuclear cells (PBMC).

C. Single-cell genotyping of different immune cell populations isolated from a primary lung tumor and blood of a patient with *TET2*-mutant TI-CH. Natural killer cells were absent in the tumor samples from this patient, so we also analyzed the blood. Cells are color coded according to their genotypes within each cell population.

D. Schematic of the experimental design profiling lung cancer metastasis in the brain and blood of patients using both whole exome sequencing and RNA sequencing of sorted immune components. CHIP is identified in the whole exome of blood samples, and mutations are analyzed in the immune components from both the blood and the tumor microenvironment.

E. *TET2* frameshift mutation is observed in the DNA of the blood and a bulk metastasis from a lung cancer patient. The variant is present in sorted neutrophils from the blood but absent in $CD4^+$ and $CD8^+$ T cells, and it is present in higher frequency in monocyte-derived macrophages and neutrophils from the TME compared to $CD4^+$ and $CD8^+$ T cells.

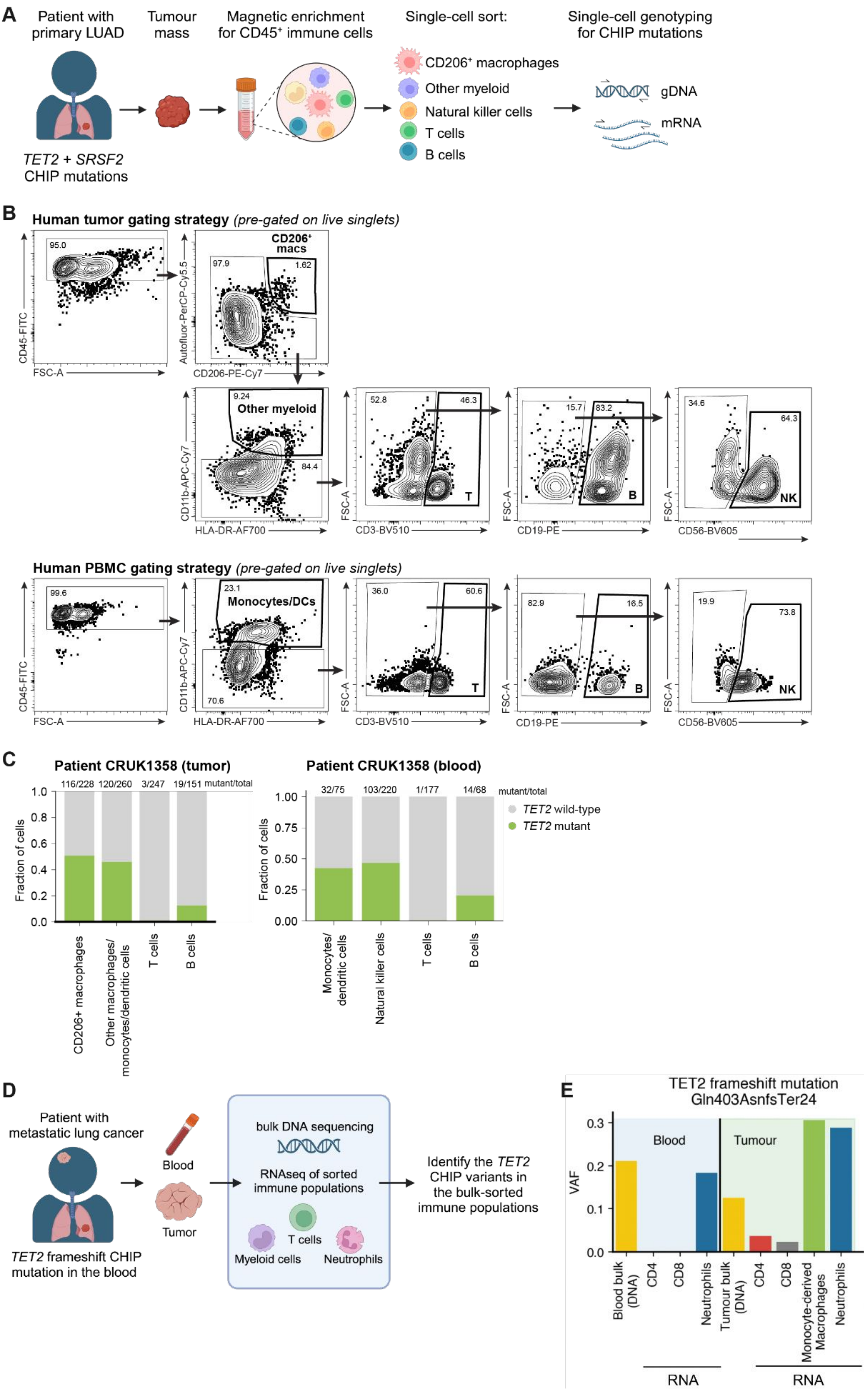
A
Patient with primary LUAD
Tumour mass
Magnetic enrichment for CD45⁺ immune cells
Single-cell sort:
Single-cell genotyping for CHIP mutations
CD206⁺ macrophages
Other myeloid
Natural killer cells
T cells
B cells
gDNA
mRNA
TET2 + SRSF2 CHIP mutations
B
Human tumor gating strategy (pre-gated on live singlets)
95.0
CD45-FITC
FSC-A
CD206⁺ macs
97.9
1.62
Autofluor-PerCP-Cy5.5
CD206-PE-Cy7
9.24
Other myeloid
84.4
CD11b-APC-Cy7
HLA-DR-AF700
52.8
46.3
T
FSC-A
CD3-BV510
15.7
83.2
B
CD19-PE
34.6
64.3
NK
CD56-BV605
Human PBMC gating strategy (pre-gated on live singlets)
99.6
23.1
Monocytes/DCs
70.6
36.0
60.6
82.9
16.5
19.9
73.8
C
Patient CRUK1358 (tumor)
116/228
120/260
3/247
19/151
mutant/total
Fraction of cells
CD206+ macrophages
Other macrophages/ monocytes/dendritic cells
T cells
B cells
Patient CRUK1358 (blood)
32/75
103/220
1/177
14/68
TET2 wild-type
TET2 mutant
Monocytes/ dendritic cells
Natural killer cells
D
Patient with metastatic lung cancer
TET2 frameshift CHIP mutation in the blood
Blood
Tumor
bulk DNA sequencing
RNAseq of sorted immune populations
T cells
Myeloid cells
Neutrophils
Identify the TET2 CHIP variants in the bulk-sorted immune populations
E
TET2 frameshift mutation Gln403AsnfsTer24
VAF
Blood
Tumour
Blood bulk (DNA)
CD4
CD8
Neutrophils
Tumour bulk (DNA)
Monocyte-derived Macrophages
RNA

Figure S13. Association between TI-CH and overall survival in TRACERx.

Kaplan-Meier probability estimates of overall survival across patients without CHIP (blue), patients with blood-only CHIP (purple), and patients with TI-CH (red) in TRACERx. P-value is from the log-rank test.

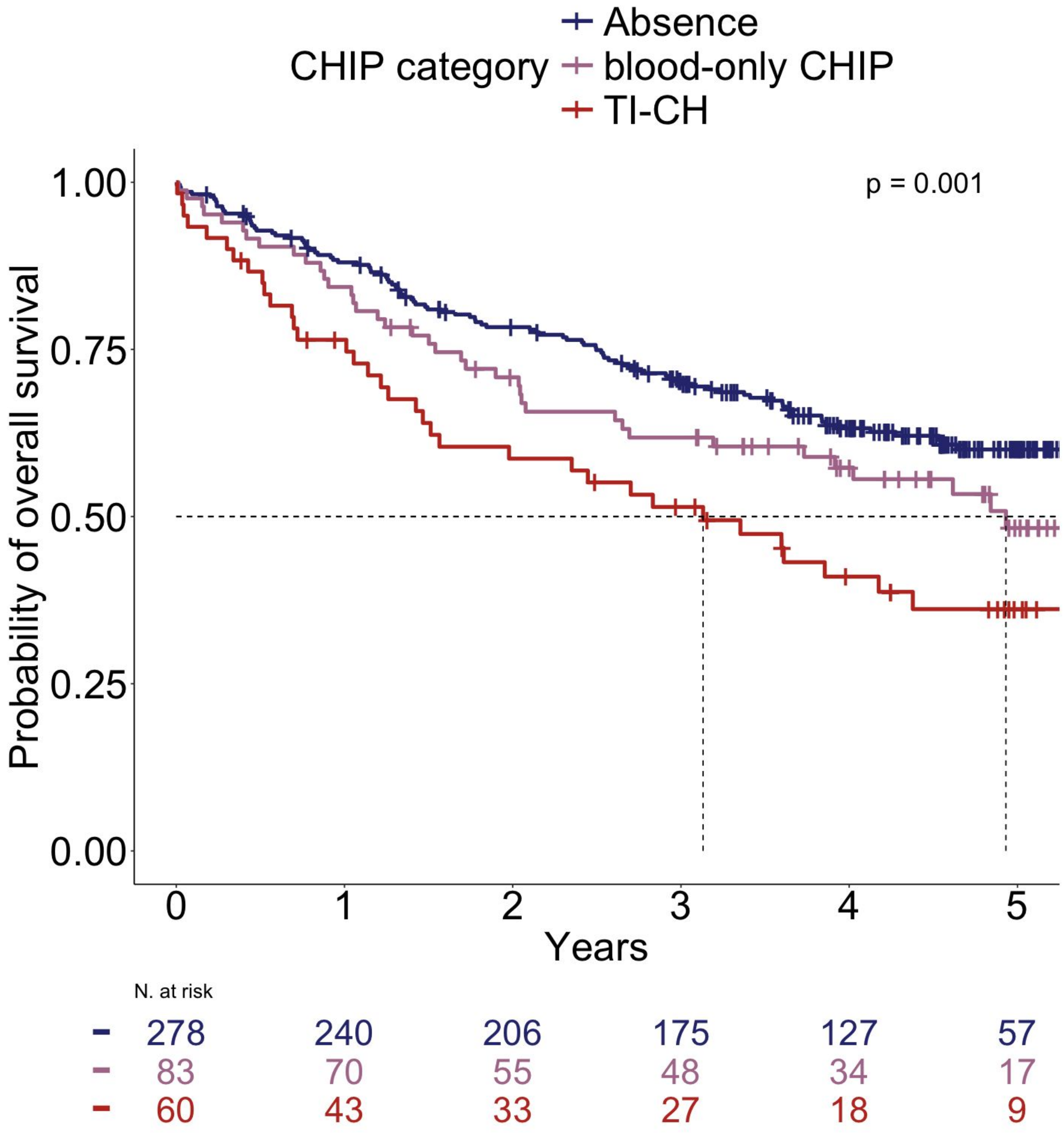

Figure S14. Multivariable Cox models for recurrence-free survival in TRACERx, in the subset of patients with CHIP (**A.**) or the full cohort (**B-E.**), accounting for demographics (age, sex), adjuvant treatment, smoking status, tumor stage, and histology, as well as NSCLC genetic drivers (**B.**), tumor purity (which inversely correlates with immune tumor infiltration) and a pathology-derived score of tumor-infiltrating neutrophils (**C.**), an estimate of the fraction of myeloid cells in the tumor microenvironment (**D.**), and the normal cell fraction (NCF, see section 1.2.4 of the Supplementary Methods). Age in years was used as a continuous variable, and divided by 5 so that the hazard ratio represents the change in risk for an increase of 5 years.

The analysis shown in **B.** was performed on the subset of 385 patients where the genomic instability index was available. The genome instability index estimates the proportion of the genome with aberrant copy number compared to the median ploidy, weighted on a per chromosome basis [PMID: 37046096]. Other NSCLC genetic drivers included the main lung tumor driver mutations (*TP53*, *KRAS*, *STK11*, and *EGFR* mutations) and oncogenic isoforms (*MET* exon 14 skipping, and *RET*, *ALK*, and *ROS1* fusions).

The analysis shown in **C.** was performed on the subset of 357 patients where a pathology-derived score of tumor-infiltrating neutrophils was available.

The analysis shown in **D.** was performed on the subset of 388 patients where the fraction of myeloid cells within the tumor microenvironment estimated from RNAseq data through CIBERSORTx deconvolution was available [PMID: 31061481].

The analysis shown in **E.** used a 4% threshold in the NCF of the CHIP mutations, i.e., less or more than 4% of CHIP-mutant cells within the non-tumoral cells, which is equivalent to the established 2% VAF criteria for CHIP in the blood. This analysis was performed on the subset of 408 patients where ploidy was successfully estimated from copy-number analysis. **E1.** Kaplan-Meier probability estimates of recurrence-free survival across patients without CHIP (blue, n=278), patients with CHIP and NCF below 4% (purple, n=26), and patients with CHIP at NCF above 4% (red, n=104). **E2.** Multivariable analysis for recurrence-free survival using NCF. Patients with NCF above 4% had shorter recurrence-free survival compared to patients with NCF below 4% or without CHIP. NCF above 4% was an independent predictor of outcomes. **E3.** Relationship between NCF, TI-CH and recurrence free survival. 57/57 (100%) of patients with TI-CH had NCF above 4% and poor outcomes (yellow). Conversely, patients with NCF above 4% were categorized both as TI-CH (55%, 57/104) and blood-only CHIP (45%, 47/104), the former with intermediate outcomes (green). This indicates that the definition of TI-CH is conservative and captures patients with a robust infiltration of mutant hematopoietic clones in the tumor microenvironment.

A Multivariable analysis for recurrence-free survival within the subset of TRACERx patients with CHIP (n=143)

Variable		N	Hazard ratio	p
**CHIP category**	blood-only CHIP	83	Reference	
	TI-CH	60	1.62 (1.02, 2.56)	0.04
**Age by 5y**		143	1.20 (1.01, 1.41)	0.03
**Sex**	Female	58	Reference	
	Male	85	1.27 (0.76, 2.13)	0.36
**Adjuvant treatment**	No	104	Reference	
	Yes	39	0.96 (0.55, 1.68)	0.90
**Smoking**	Current	55	Reference	
	Former/Never	88	0.67 (0.40, 1.12)	0.13
**Stage**	I	77	Reference	
	II	44	4.49 (2.53, 7.97)	<0.001
	III	22	3.56 (1.80, 7.03)	<0.001
**Histology**	LUAD	80	Reference	
	LUSC	50	1.24 (0.74, 2.08)	0.41
	Other	13	1.45 (0.69, 3.06)	0.33

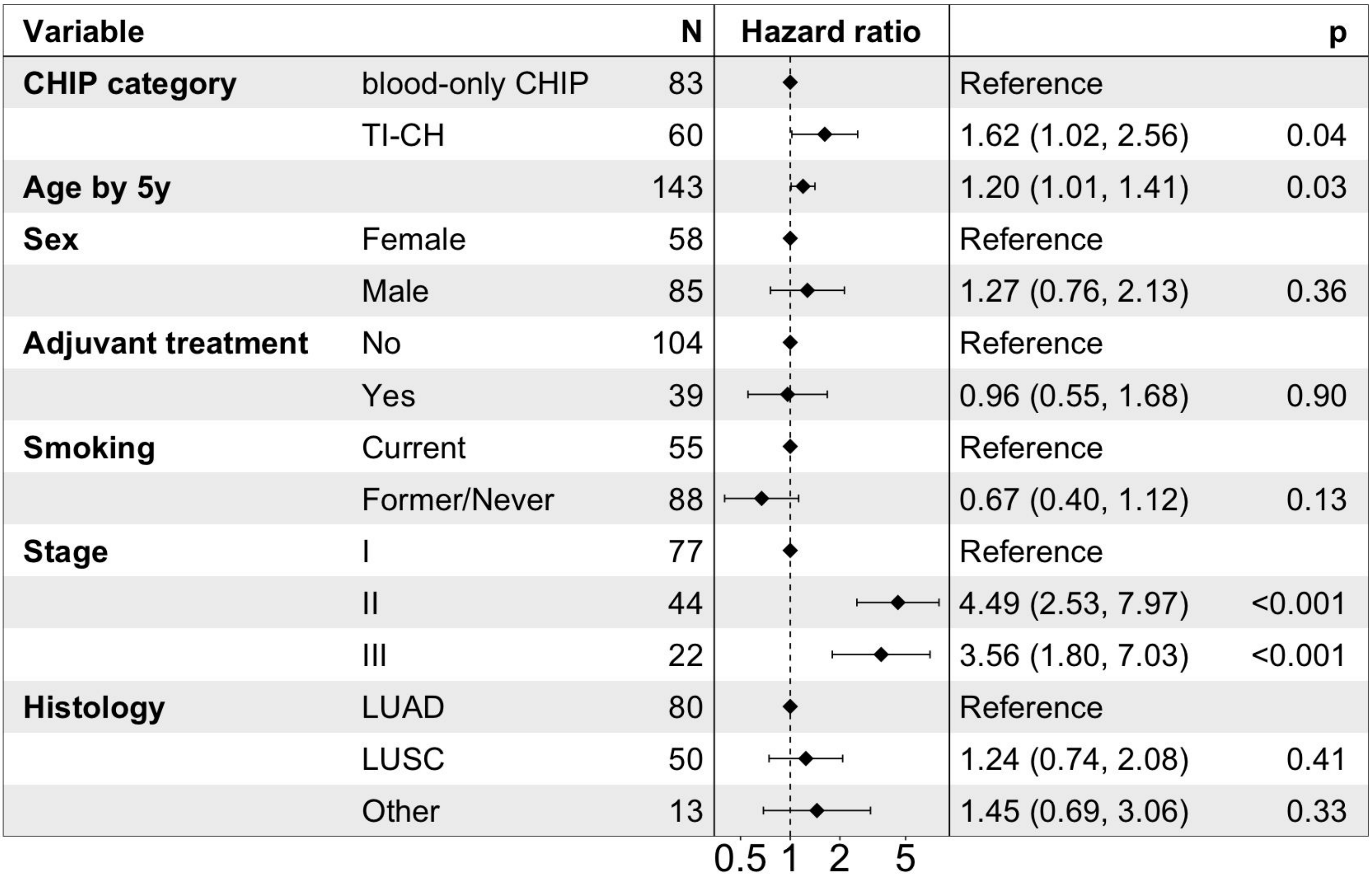

B Multivariable analysis in TRACERx for recurrence-free survival accounting for the NSCLC genetic drivers

Variable		N	Hazard ratio	p
**CHIP category**	Absence	254	Reference	
	blood-only CHIP	75	1.15 (0.78, 1.70)	0.49
	TI-CH	56	2.13 (1.43, 3.17)	<0.001
**Age by 5y**		385	1.12 (1.01, 1.24)	0.03
**Sex**	Female	172	Reference	
	Male	213	1.26 (0.92, 1.72)	0.15
**Adjuvant treatment**	No	263	Reference	
	Yes	122	0.96 (0.67, 1.39)	0.84
**Smoking**	Current	159	Reference	
	Former	200	0.91 (0.65, 1.26)	0.56
	Never	26	0.36 (0.16, 0.83)	0.02
**Stage**	I	192	Reference	
	II	120	2.14 (1.44, 3.18)	<0.001
	III	73	4.36 (2.90, 6.57)	<0.001
**Histology**	LUAD	218	Reference	
	LUSC	125	1.30 (0.86, 1.94)	0.21
	Other	42	2.13 (1.32, 3.43)	0.002
**TP53 lung driver mutation**	Wild type	132	Reference	
	Mutant	253	0.74 (0.52, 1.07)	0.11
**KRAS lung driver mutation**	Wild type	274	Reference	
	Mutant	111	1.24 (0.83, 1.86)	0.29
**STK11 lung driver mutation**	Wild type	324	Reference	
	Mutant	61	0.99 (0.65, 1.51)	0.96
**EGFR lung driver mutation**	Wild type	357	Reference	
	Mutant	28	0.92 (0.46, 1.87)	0.82
**Oncogenic Isoforms**	Absence	367	Reference	
	Presence	18	1.62 (0.83, 3.19)	0.16
**Genome Instability Index**		385	0.78 (0.29, 2.09)	0.62

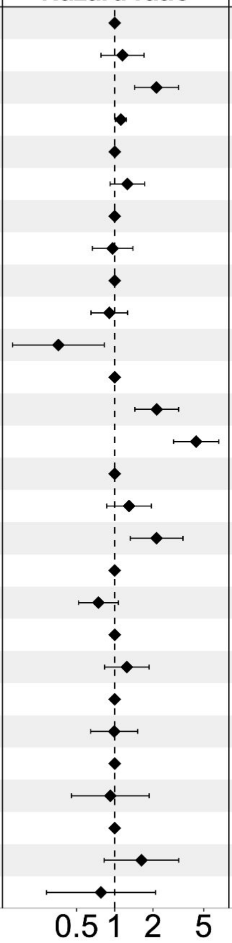

C

Multivariable analysis in TRACERx for recurrence-free survival accounting for purity and tumor-infiltrating neutrophils

Variable		N	Hazard ratio	p
**CHIP category**	Absence	239	Reference	
	blood-only CHIP	66	1.29 (0.87, 1.91)	0.2
	TI-CH	52	1.88 (1.25, 2.81)	0.002
**Age by 5y**		357	1.08 (0.98, 1.20)	0.13
**Sex**	Female	157	Reference	
	Male	200	1.04 (0.76, 1.43)	0.79
**Adjuvant treatment**	No	235	Reference	
	Yes	122	0.78 (0.54, 1.14)	0.2
**Smoking**	Current	152	Reference	
	Former	183	1.08 (0.77, 1.52)	0.65
	Never	22	0.78 (0.38, 1.63)	0.51
**Stage**	I	166	Reference	
	II	116	1.89 (1.26, 2.84)	0.002
	III	75	3.59 (2.36, 5.47)	<0.001
**Histology**	LUAD	194	Reference	
	LUSC	120	0.98 (0.69, 1.40)	0.92
	Other	43	1.98 (1.27, 3.08)	0.002
**Tumor Purity**		357	0.98 (0.43, 2.24)	0.97
**Infiltrating Neutrophils**	low	226	Reference	
	high	131	1.70 (1.22, 2.38)	0.002

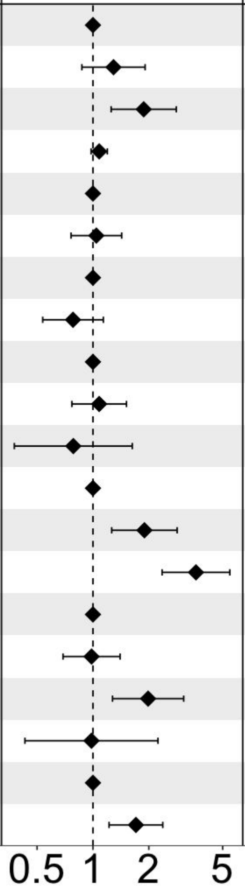

D

Multivariable analysis in TRACERx for recurrence-free survival accounting for the fraction of myeloid cells in the tumor

Variable		N	Hazard ratio	p
**CHIP category**	Absence	255	Reference	
	blood-only CHIP	76	1.26 (0.87, 1.83)	0.21
	TI-CH	57	1.86 (1.26, 2.74)	0.002
**Age by 5y**		388	1.10 (1.00, 1.21)	0.05
**Sex**	Female	168	Reference	
	Male	220	1.13 (0.84, 1.53)	0.43
**Adjuvant treatment**	No	259	Reference	
	Yes	129	0.85 (0.59, 1.21)	0.35
**Smoking**	Current	169	Reference	
	Former	193	1.00 (0.73, 1.38)	0.99
	Never	26	0.55 (0.27, 1.13)	0.1
**Stage**	I	186	Reference	
	II	126	2.27 (1.55, 3.33)	<0.001
	III	76	4.00 (2.67, 5.98)	<0.001
**Histology**	LUAD	218	Reference	
	LUSC	123	1.07 (0.77, 1.48)	0.7
	Other	47	1.77 (1.16, 2.70)	0.008
**Estimated % of myeloid cells**		388	1.03 (0.88, 1.21)	0.73

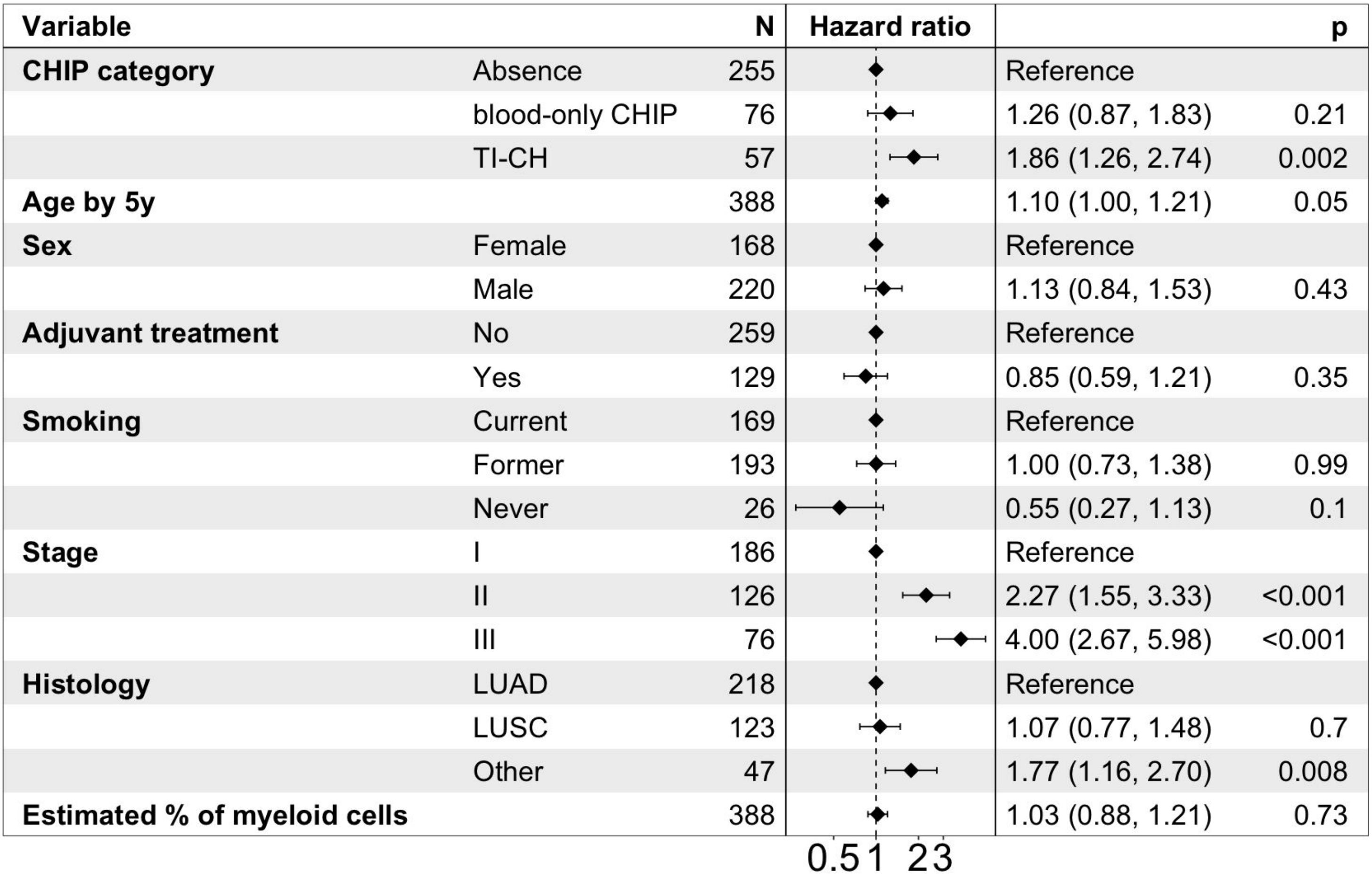

E

Univariate and multivariable analysis in TRACERx for recurrence-free survival using the normal cell fraction (NCF) of CHIP mutations

E1.

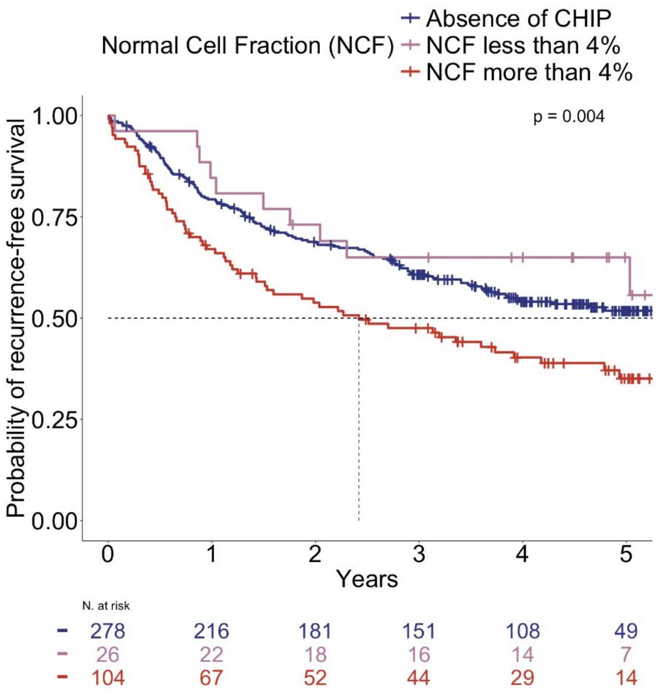

E2.

Variable		N	Hazard ratio		p
NCF category	Absence of CHIP	278		Reference	
	NCF less than 4%	26		1.03 (0.55, 1.93)	0.93
	NCF more than 4%	104		1.58 (1.15, 2.18)	0.005
Age by 5y		408		1.12 (1.02, 1.23)	0.02
Sex	Female	183		Reference	
	Male	225		1.05 (0.78, 1.41)	0.77
Adjuvant treatment	No	280		Reference	
	Yes	128		0.82 (0.57, 1.17)	0.27
Smoking	Current	174		Reference	
	Former/Never	234		0.82 (0.60, 1.12)	0.21
Stage	I	205		Reference	
	II	127		2.10 (1.43, 3.07)	<0.001
	III	76		4.19 (2.83, 6.21)	<0.001
Histology	LUAD	230		Reference	
	LUSC	131		1.31 (0.95, 1.81)	0.09
	Other	47		2.06 (1.36, 3.13)	<0.001
			0.5 1 2 5		

E3.

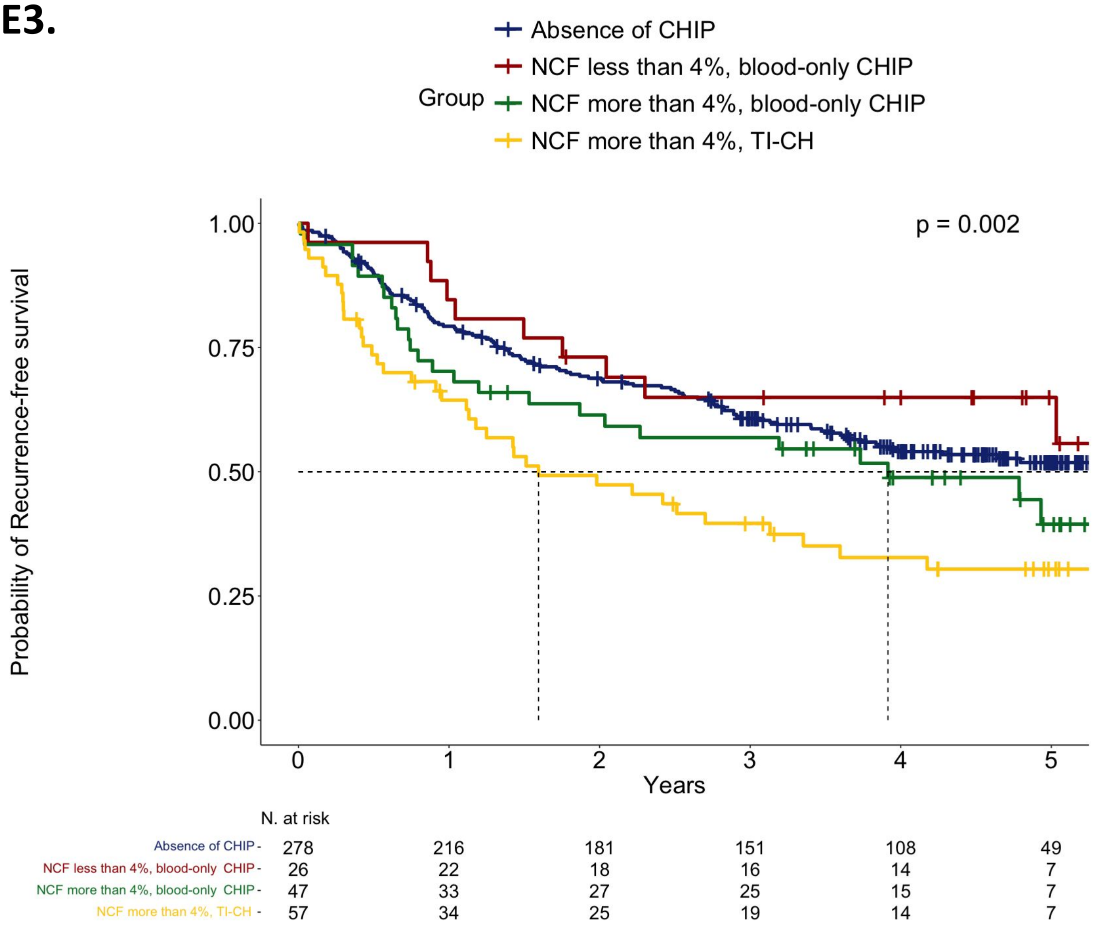

Figure S15. Cause-specific multivariable Cox models across several endpoints in TRACERx, for the risk of all-cause mortality (**A**), the risk of lung-cancer related death (**B**), and the risk of tumor recurrence or a new primary lung cancer (**C**). Models are adjusted for demographics (age, sex), adjuvant treatment, smoking status, tumor stage, and histology. Age in years was used as a continuous variable, and divided by 5 so that the hazard ratio represents the change in risk for an increase of 5 years.

A

Risk of all-cause mortality

Variable		N	Hazard ratio	p
**CHIP category**	Absence	278	Reference	
	blood-only CHIP	83	1.40 (0.96, 2.04)	0.08
	TI-CH	60	1.94 (1.30, 2.90)	0.001
**Age by 5y**		421	1.13 (1.02, 1.25)	0.02
**Sex**	Female	188	Reference	
	Male	233	1.01 (0.73, 1.38)	0.97
**Adjuvant treatment**	No	287	Reference	
	Yes	134	0.62 (0.43, 0.90)	0.01
**Smoking**	Current	180	Reference	
	Former	211	0.96 (0.68, 1.34)	0.8
	Never	30	0.41 (0.18, 0.93)	0.03
**Stage**	I	210	Reference	
	II	132	2.77 (1.86, 4.14)	<0.001
	III	79	5.15 (3.38, 7.85)	<0.001
**Histology**	LUAD	240	Reference	
	LUSC	134	1.30 (0.93, 1.83)	0.13
	Other	47	2.35 (1.53, 3.63)	<0.001

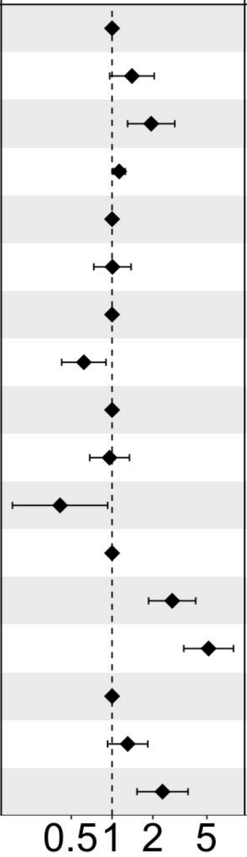

B

Risk of lung-cancer related death

Variable		N	Hazard ratio	p
**CHIP category**	Absence	278	Reference	
	blood-only CHIP	83	1.40 (0.96, 2.04)	0.08
	TI-CH	60	1.94 (1.30, 2.90)	0.001
**Age by 5y**		421	1.13 (1.02, 1.25)	0.02
**Sex**	Female	188	Reference	
	Male	233	1.01 (0.73, 1.38)	0.97
**Adjuvant treatment**	No	287	Reference	
	Yes	134	0.62 (0.43, 0.90)	0.01
**Smoking**	Current	180	Reference	
	Former	211	0.96 (0.68, 1.34)	0.8
	Never	30	0.41 (0.18, 0.93)	0.03
**Stage**	I	210	Reference	
	II	132	2.77 (1.86, 4.14)	<0.001
	III	79	5.15 (3.38, 7.85)	<0.001
**Histology**	LUAD	240	Reference	
	LUSC	134	1.30 (0.93, 1.83)	0.13
	Other	47	2.35 (1.53, 3.63)	<0.001

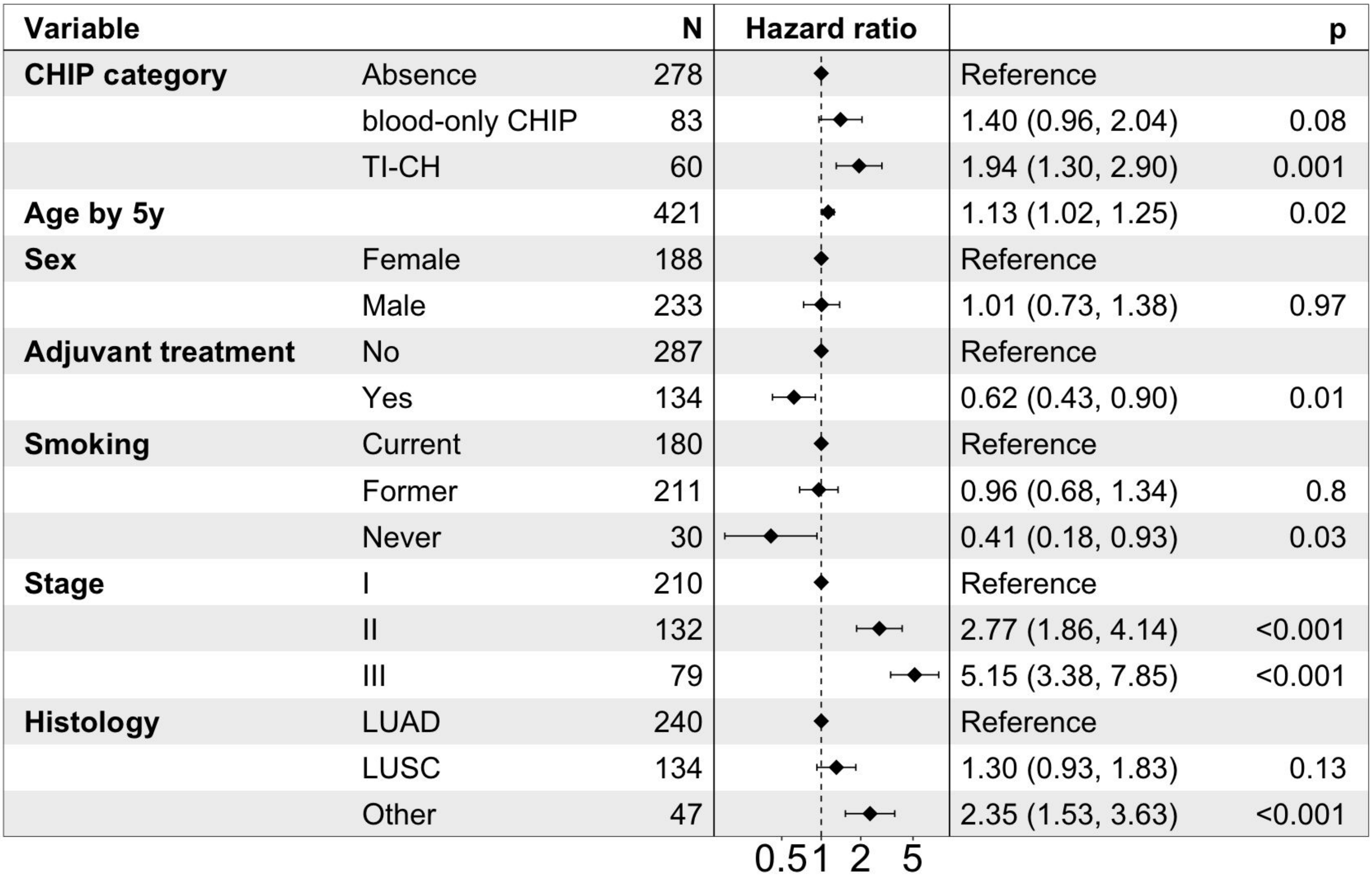

C

Risk of tumor recurrence or a new primary lung cancer

Variable		N	Hazard ratio	p
**CHIP category**	Absence	278	Reference	
	blood-only CHIP	83	1.26 (0.84, 1.89)	0.26
	TI-CH	60	1.76 (1.13, 2.75)	0.01
**Age by 5y**		421	1.08 (0.97, 1.19)	0.16
**Sex**	Female	188	Reference	
	Male	233	0.98 (0.71, 1.36)	0.92
**Adjuvant treatment**	No	287	Reference	
	Yes	134	1.10 (0.74, 1.63)	0.65
**Stage**	I	210	Reference	
	II	132	2.12 (1.36, 3.30)	<0.001
	III	79	4.27 (2.74, 6.65)	<0.001
**Histology**	LUAD	240	Reference	
	LUSC	134	1.30 (0.92, 1.85)	0.14
	Other	47	1.92 (1.18, 3.11)	0.008

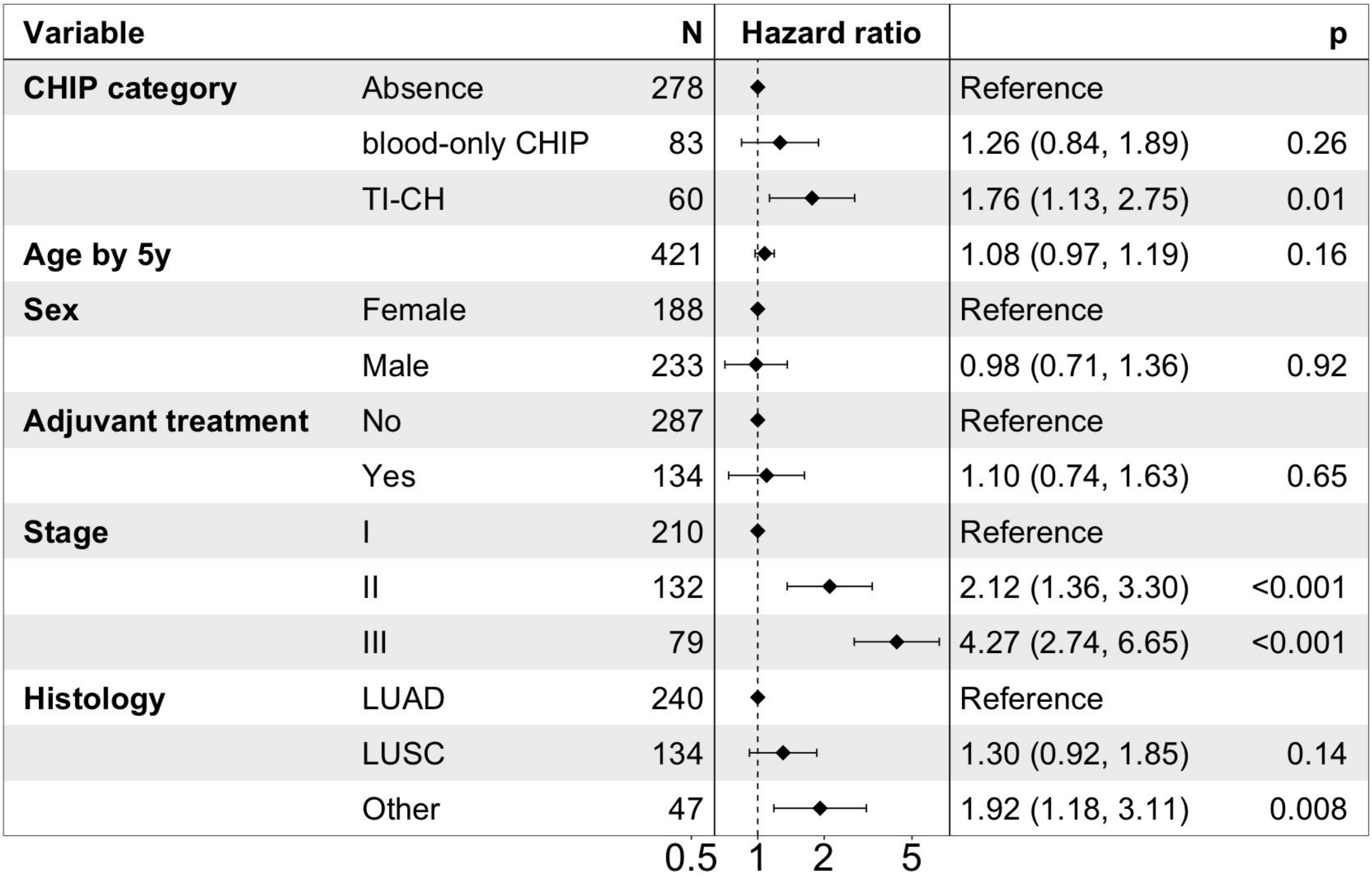

Figure S16. Ratio of the variant allele fraction (VAF) in tumor over blood of the CHIP mutations in a multivariable Cox model for recurrence-free survival in TRACERx. The model accounts for demographics (age, sex), adjuvant treatment, smoking status, tumor stage, and histology. Age in years was used as a continuous variable, and divided by 5 so that the hazard ratio represents the change in risk for an increase of 5 years.

Risk of death or tumor recurrence in patients with CHIP (n=143)

Variable		N	Hazard ratio		p
**VAF Ratio Tumor over Blood**		143		2.27 (1.01, 5.11)	0.05
**Age by 5y**		143		1.18 (1.00, 1.40)	0.05
**Sex**	Female	58		Reference	
	Male	85		1.28 (0.77, 2.12)	0.34
**Adjuvant treatment**	No	104		Reference	
	Yes	39		0.90 (0.51, 1.57)	0.71
**Smoking**	Current	55		Reference	
	Former/Never	88		0.71 (0.42, 1.19)	0.19
**Stage**	I	77		Reference	
	II	44		4.24 (2.39, 7.51)	<0.001
	III	22		3.17 (1.56, 6.43)	0.001
**Histology**	LUAD	80		Reference	
	LUSC	50		1.17 (0.69, 1.99)	0.56
	Other	13		1.41 (0.66, 3.01)	0.37

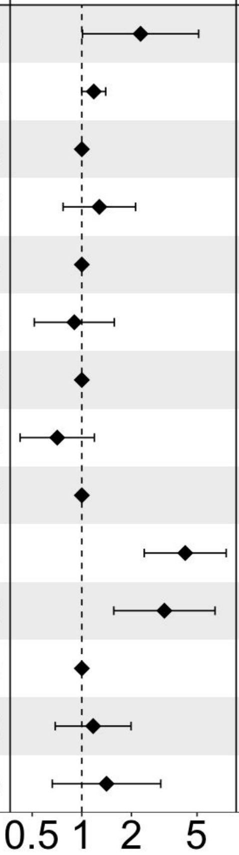

Figure S17. Multivariable Cox model for the risk of all-cause mortality in the MSK-IMPACT validation cohort of stage I-III NSCLC patients. The model accounts for demographics (age, sex, race), prior treatment, smoking status, and histology. The validation cohort consisted of 2,602 patients with stage I-III NSCLC from the MSK-IMPACT cohort, from which 1,890 had available overall survival data. Age in years was used as a continuous variable, and divided by 5 so that the hazard ratio represents the change in risk for an increase of 5 years.

Risk of all-cause mortality
NSCLC validation cohort

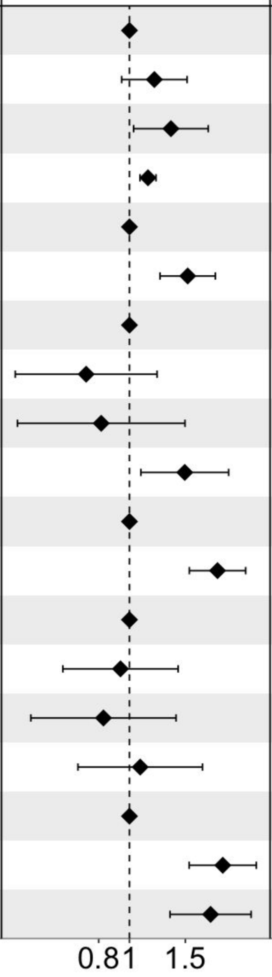

Variable		N	Hazard ratio		p
**CHIP Category**	Absence	1233		Reference	
	blood-only CHIP	417		1.20 (0.94, 1.52)	0.14
	TI-CH	240		1.35 (1.03, 1.77)	0.03
**Age by 5y**		1890		1.14 (1.08, 1.21)	<0.001
**Sex**	Female	1155		Reference	
	Male	735		1.53 (1.25, 1.87)	<0.001
**Race**	White	1333		Reference	
	Asian	112		0.73 (0.44, 1.22)	0.23
	Black	61		0.81 (0.44, 1.50)	0.51
	Other/Unknown	384		1.49 (1.09, 2.05)	0.01
**Prior Treatment**	No	1454		Reference	
	Yes/Unknown	436		1.89 (1.54, 2.32)	<0.001
**Smoking**	Current	90		Reference	
	Former	729		0.94 (0.62, 1.42)	0.76
	Never	195		0.83 (0.49, 1.40)	0.48
	Unknown	876		1.08 (0.69, 1.70)	0.74
**Histology**	LUAD	1419		Reference	
	LUSC	263		1.97 (1.54, 2.51)	<0.001
	Other	208		1.80 (1.34, 2.42)	<0.001

Figure S18. CHIP in 31,556 patients with matched blood and primary tumors profiled with MSK-IMPACT.

A. Frequency distribution of CHIP mutated genes in the MSK-IMPACT cohort of patients with matched blood and primary tumors analyzed (n=31,556).

B. Multivariable logistic regression for the presence of CHIP in MSK-IMPACT. Explanatory covariates include demographic variables (age, sex, race), smoking status, prior treatment, and tumor stage. Age in years was used as a continuous variable, and divided by 5.

A

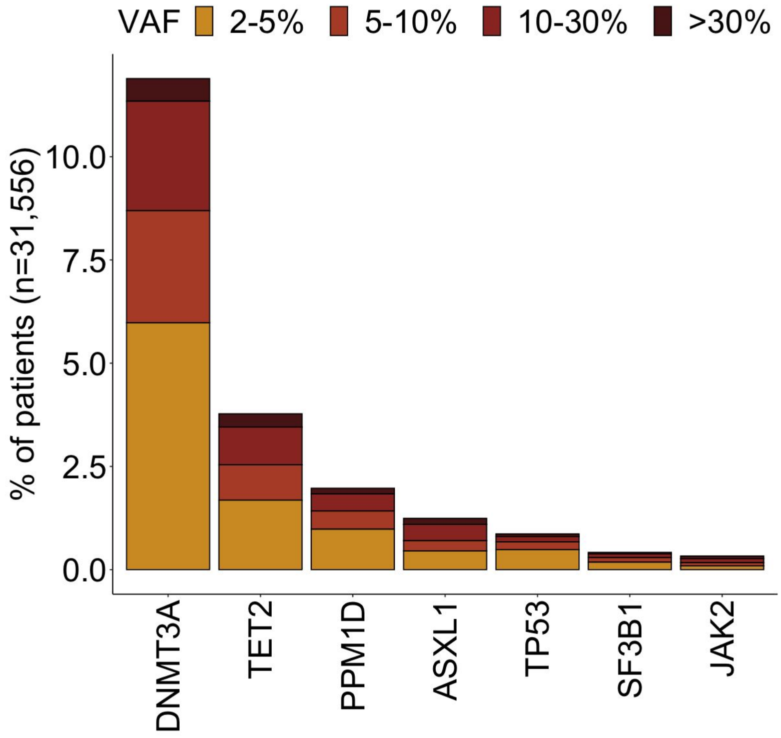

B

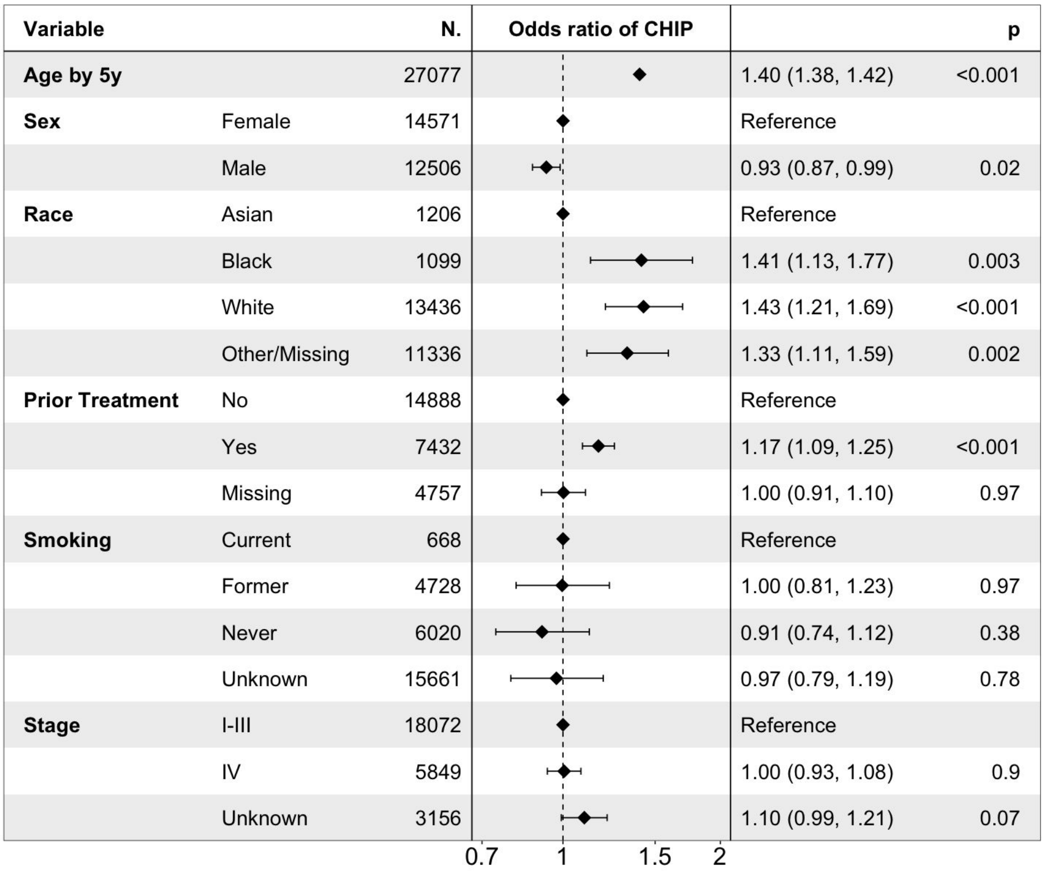

Variable		N.	Odds ratio of CHIP		p
**Age by 5y**		27077		1.40 (1.38, 1.42)	<0.001
**Sex**	Female	14571		Reference	
	Male	12506		0.93 (0.87, 0.99)	0.02
**Race**	Asian	1206		Reference	
	Black	1099		1.41 (1.13, 1.77)	0.003
	White	13436		1.43 (1.21, 1.69)	<0.001
	Other/Missing	11336		1.33 (1.11, 1.59)	0.002
**Prior Treatment**	No	14888		Reference	
	Yes	7432		1.17 (1.09, 1.25)	<0.001
	Missing	4757		1.00 (0.91, 1.10)	0.97
**Smoking**	Current	668		Reference	
	Former	4728		1.00 (0.81, 1.23)	0.97
	Never	6020		0.91 (0.74, 1.12)	0.38
	Unknown	15661		0.97 (0.79, 1.19)	0.78
**Stage**	I-III	18072		Reference	
	IV	5849		1.00 (0.93, 1.08)	0.9
	Unknown	3156		1.10 (0.99, 1.21)	0.07

Figure S19. Gene frequency distribution in CHIP and TI-CH from the primary pan-cancer MSK IMPACT cohort (n=31,556). Frequency of mutated genes for the full cohort (top row) and across cancer types within patients with CHIP (left, green) and within patients with TI-CH (right, blue). Only cancer types with more than 150 patients in total were included in the analysis.

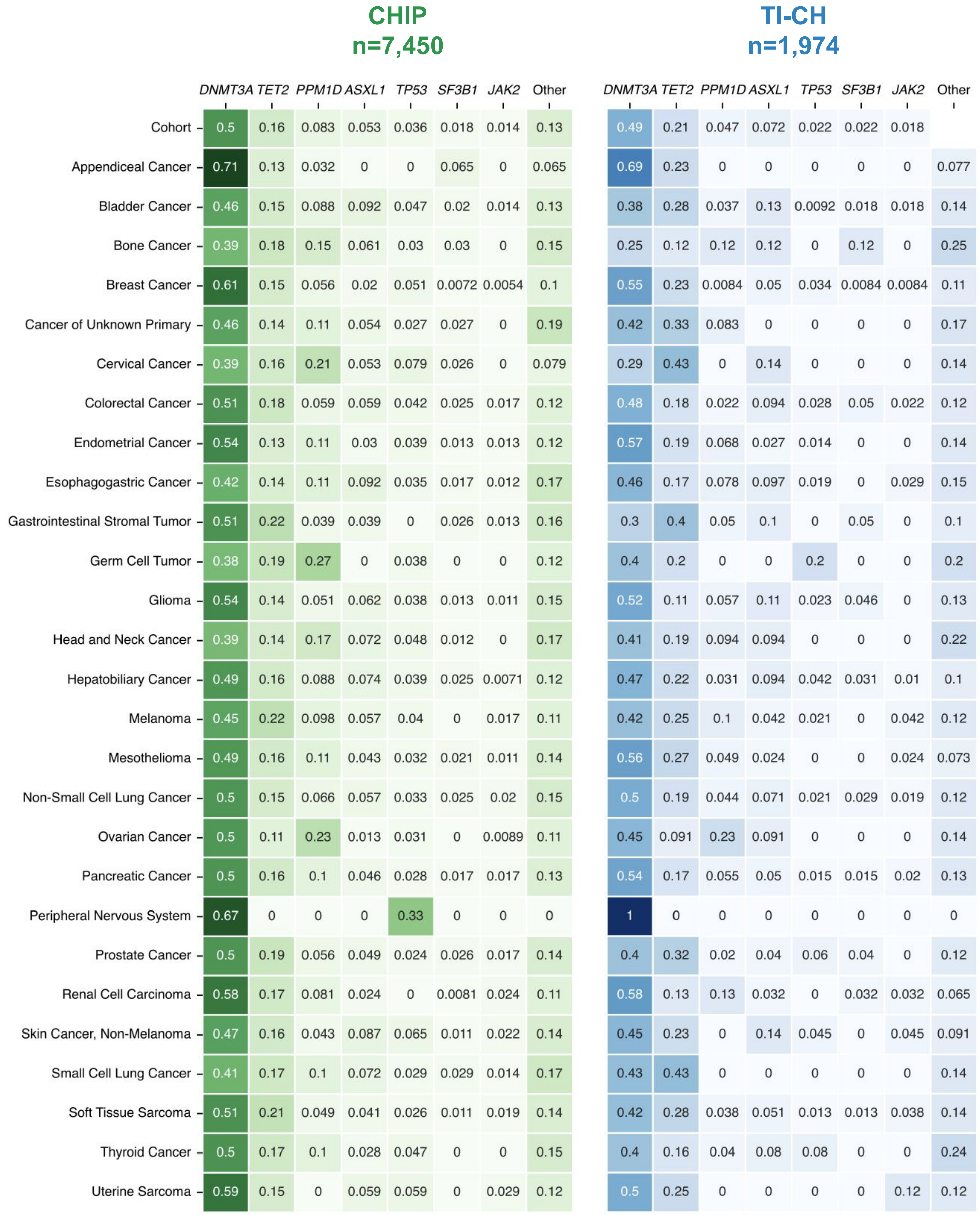

Figure S20. Multivariable Cox model for the risk of all-cause mortality in the primary MSK-IMPACT cohort of patients with stage I-III disease. The model accounts for demographics (age, sex, race), prior treatment, and smoking status. Age in years was used as a continuous variable, and divided by 5 so that the hazard ratio represents the change in risk for an increase of 5 years.

Risk of all-cause mortality
Pan-cancer Stage I-III cohort (n=14,694)

Variable		N	Hazard ratio	p
**CHIP Category**	Absence	11241	Reference	
	blood-only CHIP	2543	1.15 (1.06, 1.25)	<0.001
	TI-CH	910	1.44 (1.28, 1.62)	<0.001
**Age by 5y**		14694	1.10 (1.08, 1.11)	<0.001
**Sex**	Female	8290	Reference	
	Male	6404	1.37 (1.29, 1.46)	<0.001
**Race**	White	8498	Reference	
	Asian	696	0.95 (0.81, 1.11)	0.50
	Black	624	1.16 (1.00, 1.35)	0.05
	Other/Unknown	4876	1.52 (1.39, 1.66)	<0.001
**Prior Treatment**	No	9586	Reference	
	Yes	4847	1.70 (1.59, 1.81)	<0.001
	Unknown	261	1.50 (1.06, 2.11)	0.02
**Smoking**	Current	436	Reference	
	Former	3019	0.93 (0.79, 1.10)	0.38
	Never	3748	0.94 (0.79, 1.10)	0.43
	Unknown	7491	0.85 (0.71, 1.00)	0.06

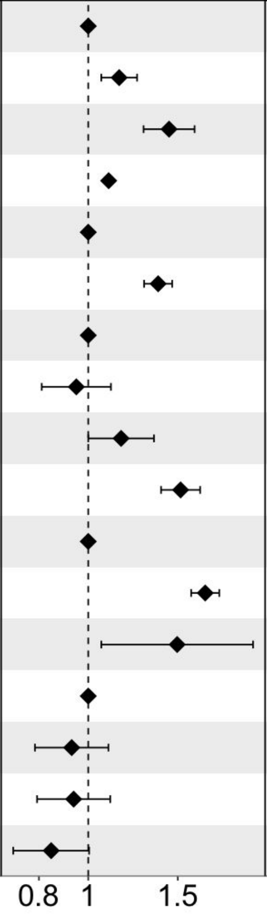

Figure S21. Ratio of the variant allele fraction (VAF) in tumor over blood of the CHIP mutations in a multivariable Cox model for overall survival in MSK-IMPACT.

The ratio was calculated for patients with CHIP (n=7,450 and 5,114 with overall survival data) from the primary MSK-IMPACT cohort (n=31,556). The model accounts for demographics (age, sex, race), prior treatment, smoking status, tumor stage, and tumor purity. Age in years was used as a continuous variable, and divided by 5 so that the hazard ratio represents the change in risk for an increase of 5 years. Similarly, tumor purity in % was used as a continuous variable, and divided by 10.

Risk of all-cause mortality
in patients with CHIP in MSK-IMPACT

Variable		N	Hazard ratio		p
**VAF Ratio Tumor over Blood**		5114		1.61 (1.31, 1.97)	<0.001
**Age by 5y**		5114		1.08 (1.06, 1.11)	<0.001
**Sex**	Female	2707		Reference	
	Male	2407		1.12 (1.03, 1.23)	0.01
**Race**	White	3232		Reference	
	Asian	182		1.02 (0.81, 1.28)	0.89
	Black	209		1.15 (0.93, 1.43)	0.20
	Other/Unknown	1491		1.27 (1.12, 1.45)	<0.001
**Prior Treatment**	No	3151		Reference	
	Yes/Unknown	1963		1.39 (1.27, 1.53)	<0.001
**Smoking**	Current	136		Reference	
	Former	1238		0.94 (0.73, 1.21)	0.64
	Never	1103		0.92 (0.71, 1.19)	0.54
	Unknown	2637		0.93 (0.72, 1.21)	0.59
**Stage**	I-III	3415		Reference	
	IV	1166		1.74 (1.57, 1.92)	<0.001
	Unknown	533		1.81 (1.55, 2.13)	<0.001
**Tumor Purity by 10%**		5114		1.06 (1.04, 1.09)	<0.001

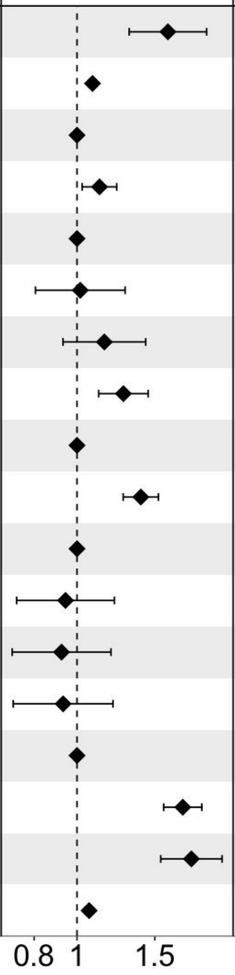

Figure S22. TI-CH in metastatic samples in MSK-IMPACT (n=17,795). Proportion of patients with CHIP (gray) and with TI-CH (red) for each cancer type. Patients with matched blood and metastatic tumor samples were selected (n=17,795), with only one metastatic sample analyzed per patient. Only cancer types with more than 150 patients were included in the analysis. On the y-axis patients are classified according to the diagnosis of the primary cancer type.

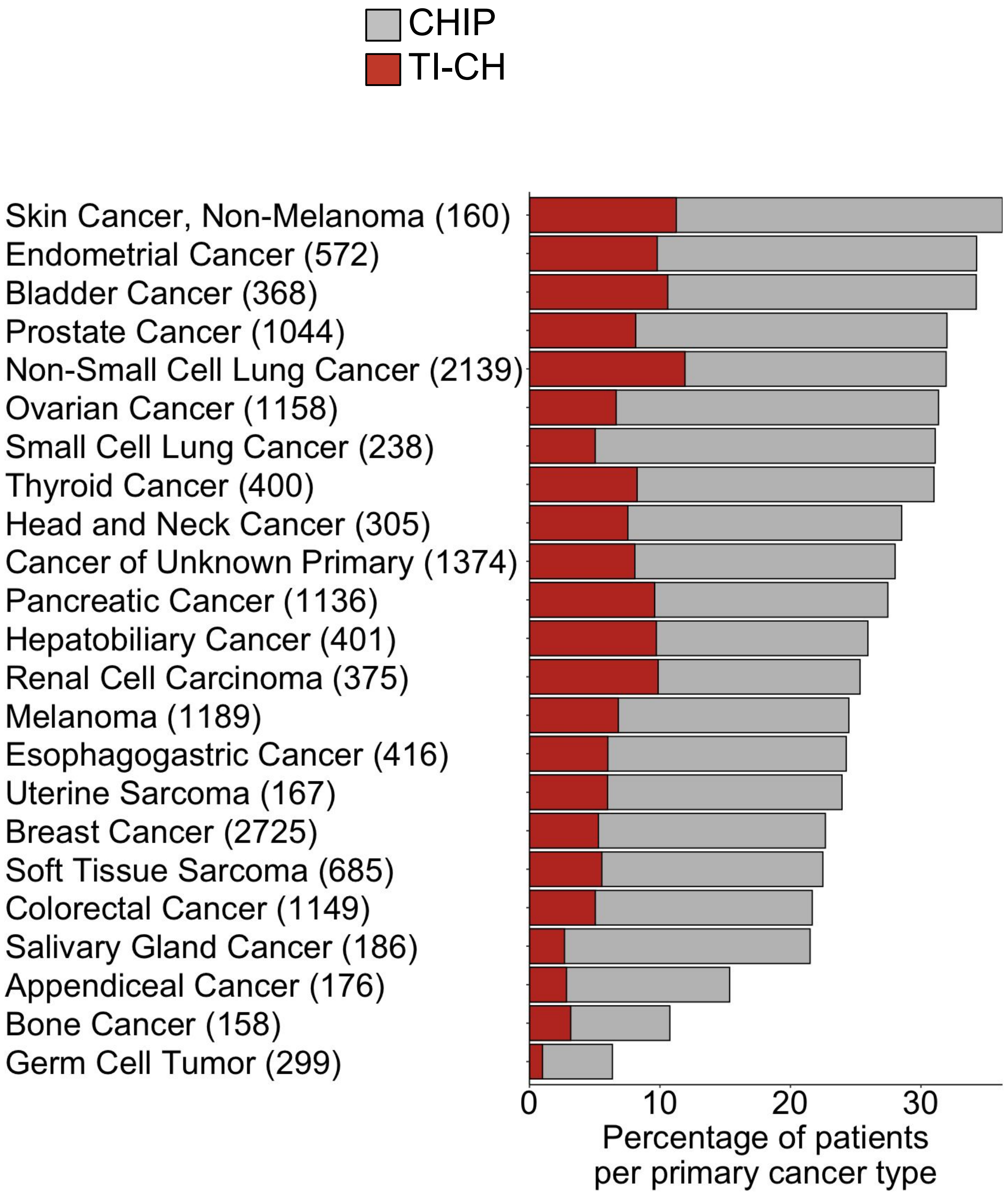

Figure S23. Longitudinal analysis of TI-CH in paired primary-metastasis and autopsy

A. TI-CH in paired primary tumors and progression metastases in 54 patients from MSK-IMPACT. Patients were positive for TI-CH in primary tumors. The VAF of the CHIP clones in both primary tumors and metastases is depicted on the y-axis. Color represents the most frequently mutated genes. The median inter-sampling time between primary tumors and metastases was 427 days (IQR 209-692).

B. TI-CH analysis in two TRACERx patients enrolled in the PEACE program. Left: representation of the number of samples and organs analyzed in two patients harboring TI-CH clones in primary tumors (CRUK0719 with *DNMT3A* and *TET2* mutations, CRUK0516 with a *DNMT3A* mutation). A total of 27 and 36 samples were analyzed for CRUK0719 and CRUK051, respectively, encompassing 9 different organs, at time of primary surgery, recurrence, and autopsy. Right: VAF distribution of CHIP clones across the different samples at time of primary surgery, recurrence, and autopsy.

A

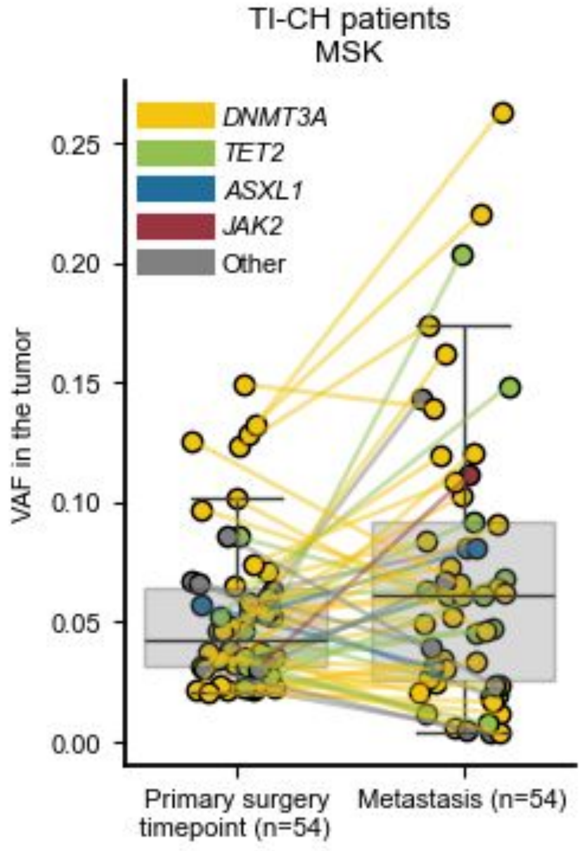

TI-CH patients
MSK
DNMT3A
TET2
ASXL1
JAK2
Other
VAF in the tumor
0.25
0.20
0.15
0.10
0.05
0.00
Primary surgery timepoint (n=54)
Metastasis (n=54)

B

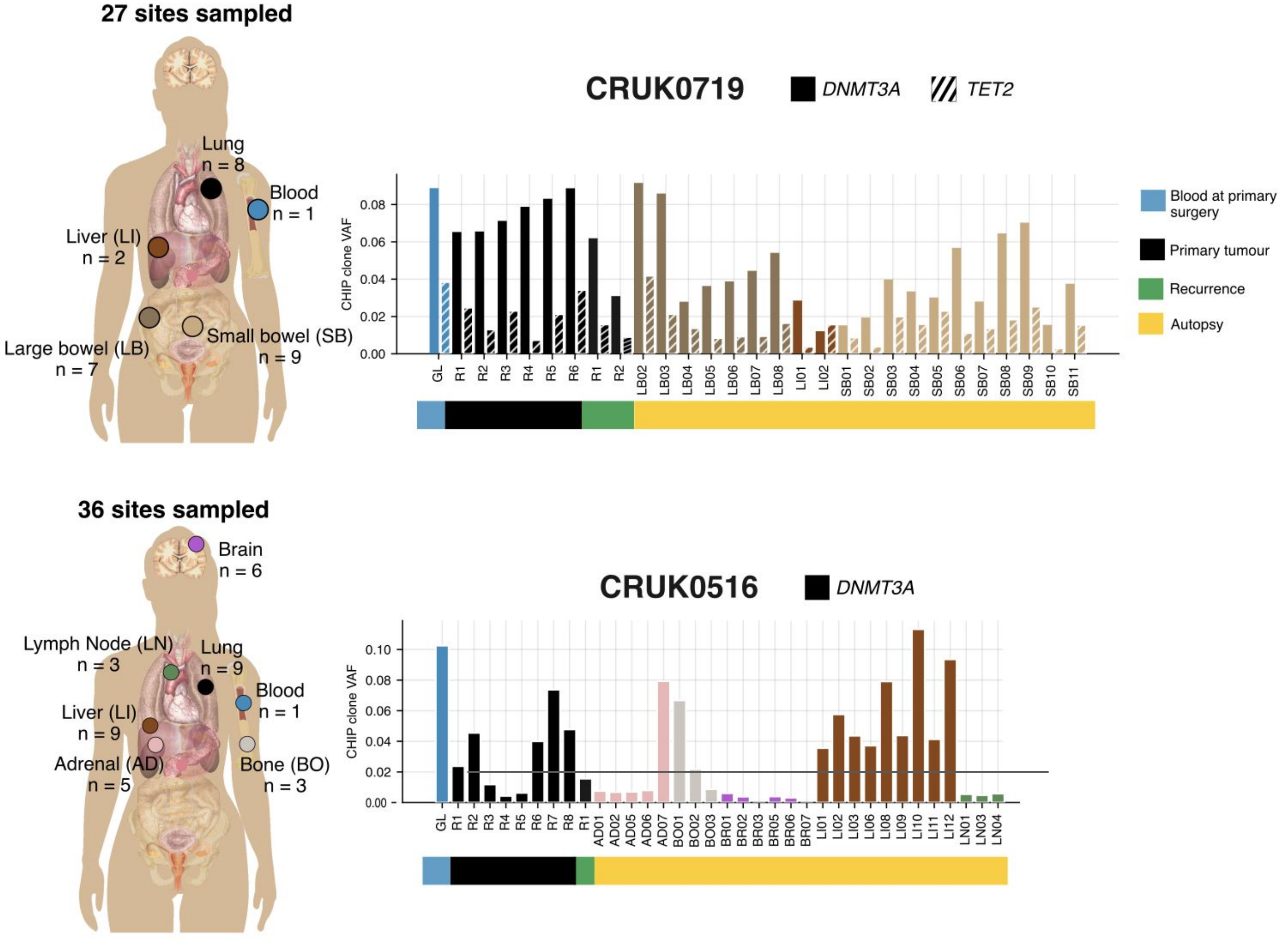

27 sites sampled
Lung n = 8
Blood n = 1
Liver (LI) n = 2
Large bowel (LB) n = 7
Small bowel (SB) n = 9
CRUK0719
DNMT3A
TET2
CHIP clone VAF
0.08
0.06
0.04
0.02
0.00
GL R1 R2 R3 R4 R5 R6 R1 R2 LB02 LB03 LB04 LB05 LB06 LB07 LB08 LI01 LI02 SB01 SB02 SB03 SB04 SB05 SB06 SB07 SB08 SB09 SB10 SB11
Blood at primary surgery
Primary tumour
Recurrence
Autopsy
36 sites sampled
Brain n = 6
Lymph Node (LN) n = 3
Lung n = 9
Blood n = 1
Liver (LI) n = 9
Adrenal (AD) n = 5
Bone (BO) n = 3
CRUK0516
DNMT3A
CHIP clone VAF
0.10
0.08
0.06
0.04
0.02
0.00
GL R1 R2 R3 R4 R5 R6 R7 R8 R1 AD01 AD02 AD05 AD06 AD07 BO01 BO02 BO03 BR01 BR02 BR03 BR05 BR06 BR07 LI01 LI02 LI03 LI06 LI08 LI09 LI10 LI11 LI12 LN01 LN03 LN04

Figure S24. Gene-specificity of TI-CH and prediction of TI-CH within patients with CHIP in the primary MSK-IMPACT cohort.

A. Mutated gene frequency distribution in CHIP (n=7,450) and TI-CH (n=1,974) in the primary MSK-IMPACT cohort (n=31,556).

B. Frequency of TI-CH within patients with gene-specific CHIP. For example, within 3,753 patients with *DNMT3A*-mutant CHIP, 926 (25%) had TI-CH.

C. Ratio of tumor over blood variant allele frequency (VAF) of the CHIP mutations in patients with CHIP. *TET2* mutations had the largest relative tumor-infiltrating clone size compared to other CHIP gene mutations (median tumor over blood VAF ratio of 0.18 versus 0.13; sum of the ranks 4.2×10^6, $p<0.001$).

D. Multivariable logistic regression for the presence of TI-CH within CHIP patients in the primary MSK-IMPACT cohort. Explanatory covariates include demographics (age, sex, race), prior treatment, smoking status, and tumor stage. Tumor purity, the variant allele fraction (VAF) of the CHIP mutations in the blood, and the type of CHIP mutated genes (*DNMT3A*, *TET2*, *ASXL1*, *PPM1D*, *TP53* or others) are also included. Age in years was used as a continuous variable, and divided by 5. Tumor purity in % was used as a continuous variable, and divided by 10.

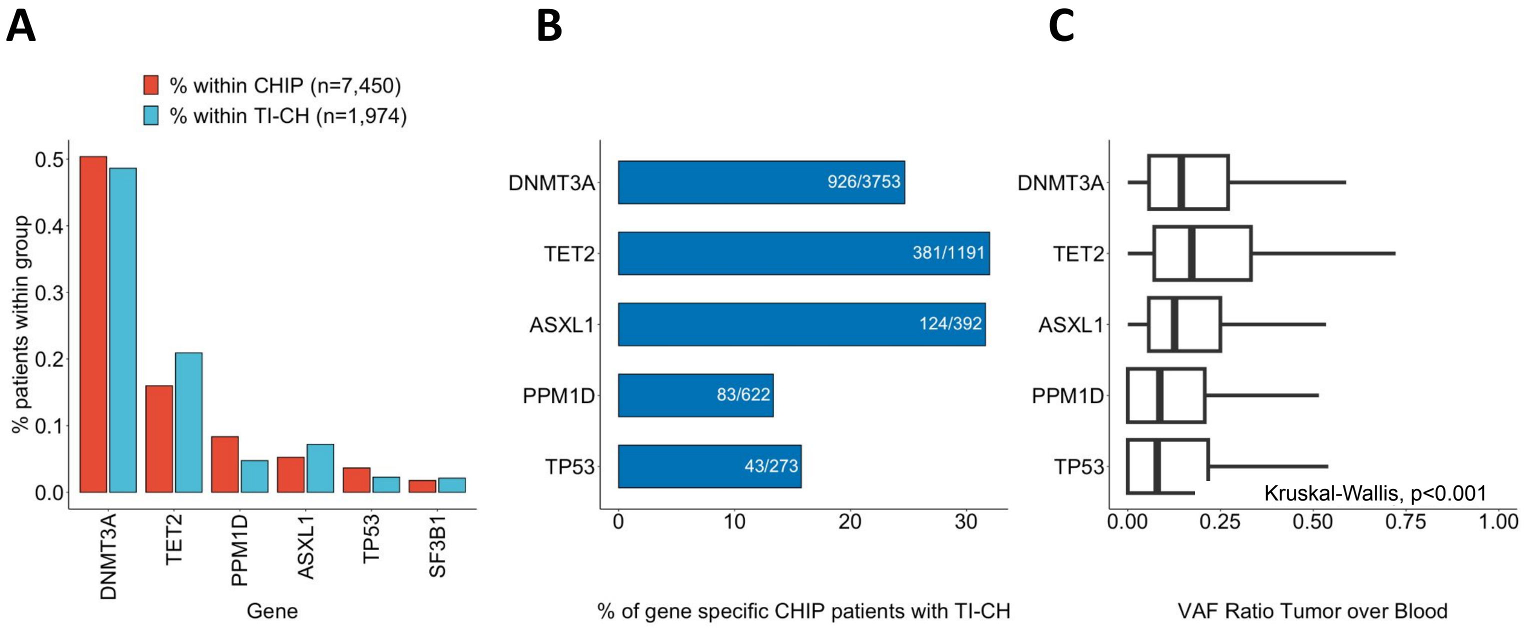

D

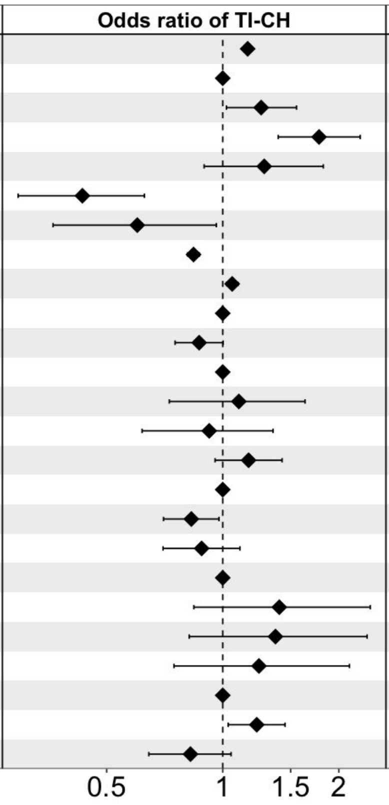

Variable		N.	Odds ratio of TI-CH	p
**VAF in the blood**		6194	1.16 (1.15, 1.17)	<0.001
**Gene**	Other	984	Reference	
	DNMT3A	3196	1.26 (1.02, 1.55)	0.03
	TET2	1004	1.78 (1.39, 2.27)	<0.001
	ASXL1	307	1.28 (0.89, 1.82)	0.17
	PPM1D	473	0.43 (0.29, 0.63)	<0.001
	TP53	230	0.60 (0.36, 0.96)	0.04
**Purity by 10%**		6194	0.84 (0.81, 0.87)	<0.001
**Age by 5y**		6194	1.06 (1.02, 1.10)	0.001
**Sex**	Female	3288	Reference	
	Male	2906	0.87 (0.75, 1.00)	0.05
**Race**	White	3330	Reference	
	Asian	186	1.10 (0.73, 1.63)	0.64
	Black	232	0.92 (0.62, 1.35)	0.69
	Other/Unknown	2446	1.17 (0.96, 1.43)	0.13
**Prior Treatment**	No	3271	Reference	
	Yes	1907	0.83 (0.70, 0.98)	0.03
	Unknown	1016	0.88 (0.70, 1.11)	0.28
**Smoking**	Current	142	Reference	
	Former	1282	1.40 (0.84, 2.41)	0.21
	Never	1143	1.37 (0.82, 2.37)	0.24
	Unknown	3627	1.24 (0.75, 2.13)	0.42
**Stage**	I-III	4156	Reference	
	IV	1387	1.23 (1.03, 1.45)	0.02
	Unknown	651	0.82 (0.64, 1.05)	0.12

Figure S25. Experimental mouse model of CHIP.

A. Schematic of the *in vivo* experiment to study lung cancer in Tet2-mutant CHIP.

B. Representative flow plot of the gating strategy to distinguish Tet2-mutant transferred cells ($CD45.2^+$), wild-type transferred cells ($CD45.1^+$)], and wild-type recipient derived cells ($CD45.1^+$ $CD45.2^+$).

C. Expansion of Tet2-mutant versus wild-type transferred cells in the blood of Tet2-mutant CHIP mice 6 and 16 weeks after bone marrow transplant (n=9 biological replicates); ***p<0.001.

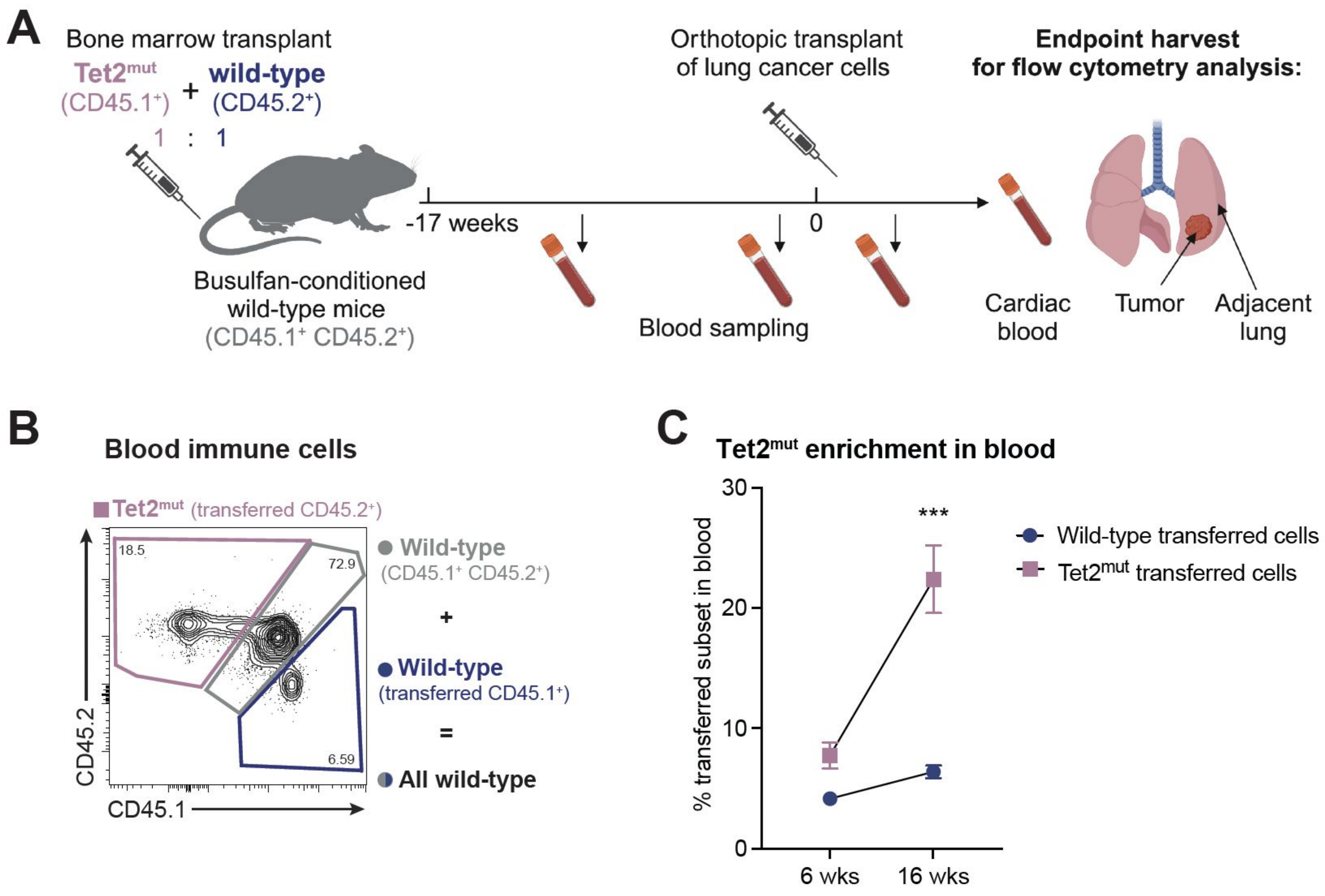

Figure S26. Flow cytometry immune cell gating strategy in the mouse model of CHIP.

A. Murine blood cell gating strategy.

B. Murine lung cell gating strategy.

C. Murine tumor cell gating strategy

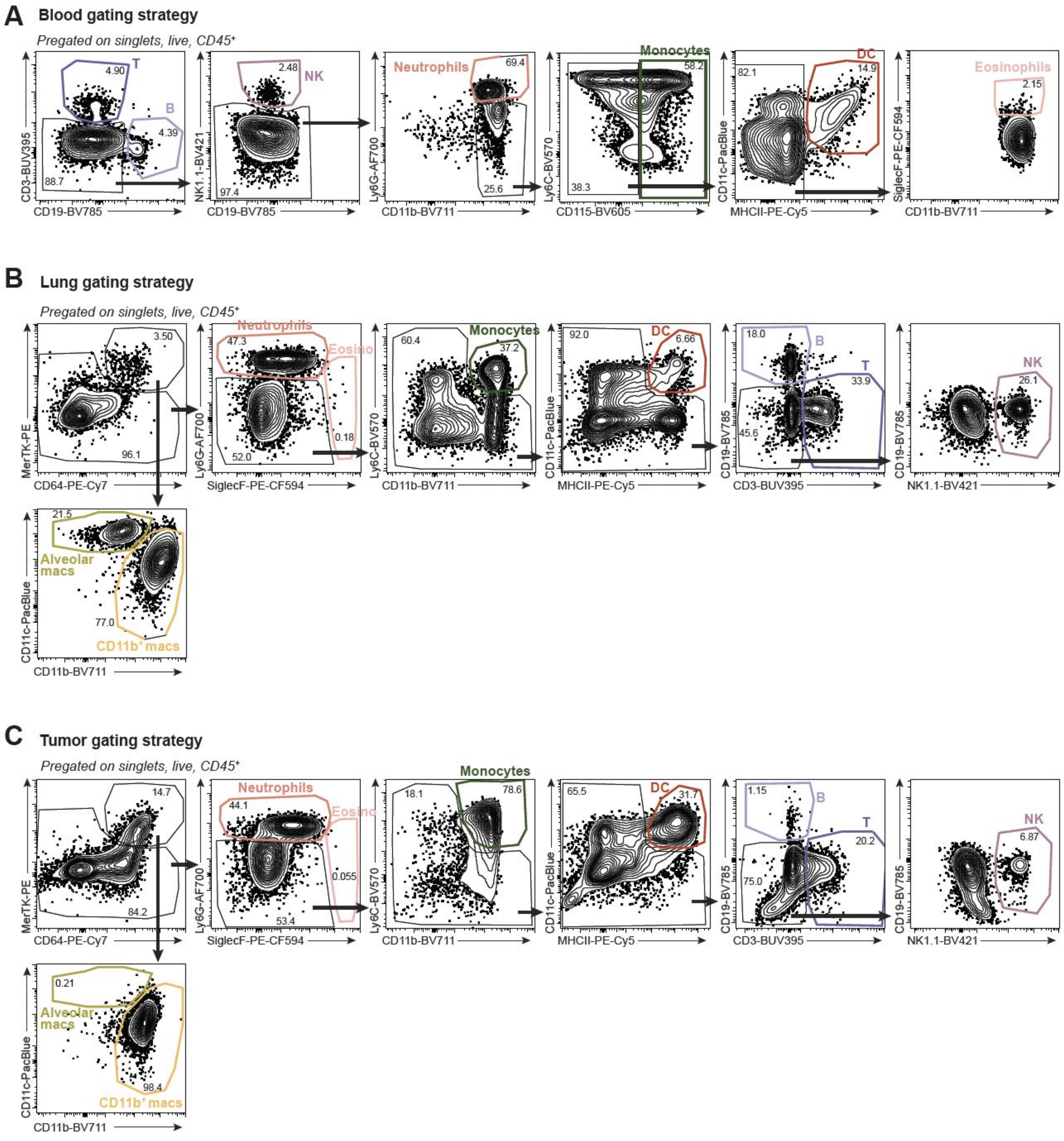

Figure S27. Impact of Tet2-mutant CHIP on the lung tumor microenvironment composition in mice.

A. Immune composition in the blood, lung and tumor of Tet2-mutant CHIP mice inoculated with 3LL lung tumor cells and analyzed at endpoint (n=9 biological replicates).

B. Percent of Tet2-mutant cells across major immune cell populations in lung tumors (n=9 biological replicates); *p<0.05, **p<0.01,***p<0.001, ns = not significant.

C-E. Correlation between percent of Tet2-mutant cells in blood and (**C**) Tet2-mutant vs wild-type mononuclear phagocyte cell infiltration in the tumor, (**D**) Tet2-mutant vs wild-type granulocyte infiltration in the tumor and (**E**) Tet2-mutant mononuclear phagocytes vs Tet2-mutant granulocyte infiltration in the tumor. The data points shown in E are the same as those corresponding to Tet2-mutant conditions from C and D. (n=9 biological replicates). Abbreviation: MNP = mononuclear phagocytes.

F. Schematic of *ex vivo* transwell experiment to study immune cell migration towards 3LL tumor cells.

G. Percent of Tet2-mutant or wild-type neutrophils that migrated to the bottom chamber in the presence or absence of 3LL lung tumor cells (blood from 1-4 mice was pooled per datapoint); ns = not significant.

H-I. *In vivo* accumulation of monocytes, $CD11b^+$ macrophages (**H**) and neutrophils (**I**) quantified as cells/gram in tumors divided by cells/gram in lungs (n=7 biological replicates); *p<0.05, ns = not significant.

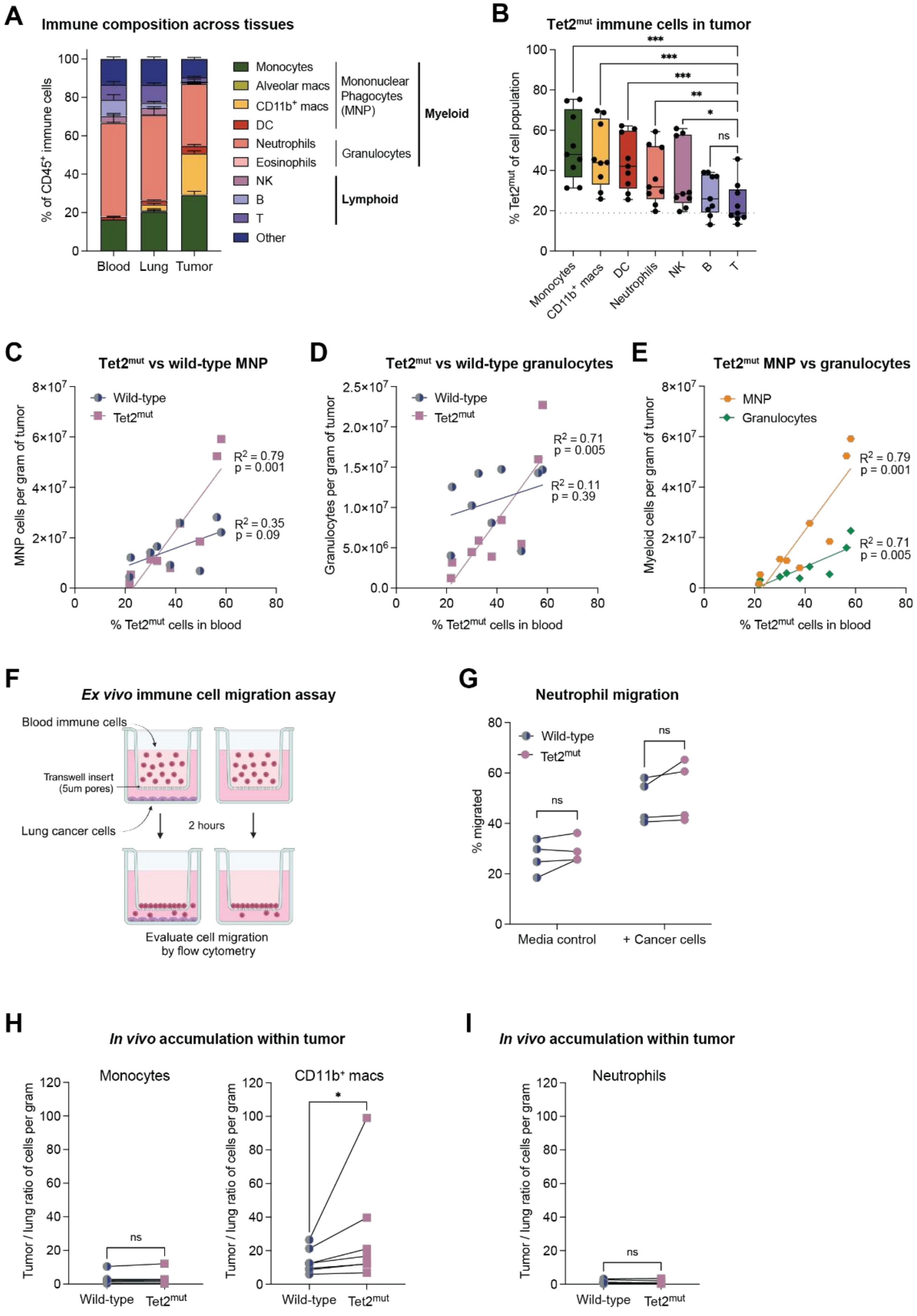
A
Immune composition across tissues
% of CD45+ immune cells
Blood
Lung
Tumor
Monocytes
Alveolar macs
CD11b+ macs
DC
Neutrophils
Eosinophils
NK
B
T
Other
Mononuclear Phagocytes (MNP)
Granulocytes
Myeloid
Lymphoid
B
Tet2mut immune cells in tumor
% Tet2mut of cell population
Monocytes
CD11b+ macs
DC
Neutrophils
NK
B
T
ns
C
Tet2mut vs wild-type MNP
MNP cells per gram of tumor
Wild-type
Tet2mut
R2 = 0.79
p = 0.001
R2 = 0.35
p = 0.09
% Tet2mut cells in blood
D
Tet2mut vs wild-type granulocytes
Granulocytes per gram of tumor
R2 = 0.71
p = 0.005
R2 = 0.11
p = 0.39
E
Tet2mut MNP vs granulocytes
Myeloid cells per gram of tumor
MNP
Granulocytes
R2 = 0.79
p = 0.001
R2 = 0.71
p = 0.005
F
Ex vivo immune cell migration assay
Blood immune cells
Transwell insert (5um pores)
Lung cancer cells
2 hours
Evaluate cell migration by flow cytometry
G
Neutrophil migration
% migrated
Media control
+ Cancer cells
H
In vivo accumulation within tumor
Monocytes
CD11b+ macs
Tumor / lung ratio of cells per gram
Wild-type
Tet2mut
I
In vivo accumulation within tumor
Neutrophils

Figure S28. Human tumor-myeloid co-culture experimental setup.

A. Schematic of the human tumor-myeloid cell co-culture experiment.

B. Human myeloid cell gating strategy.

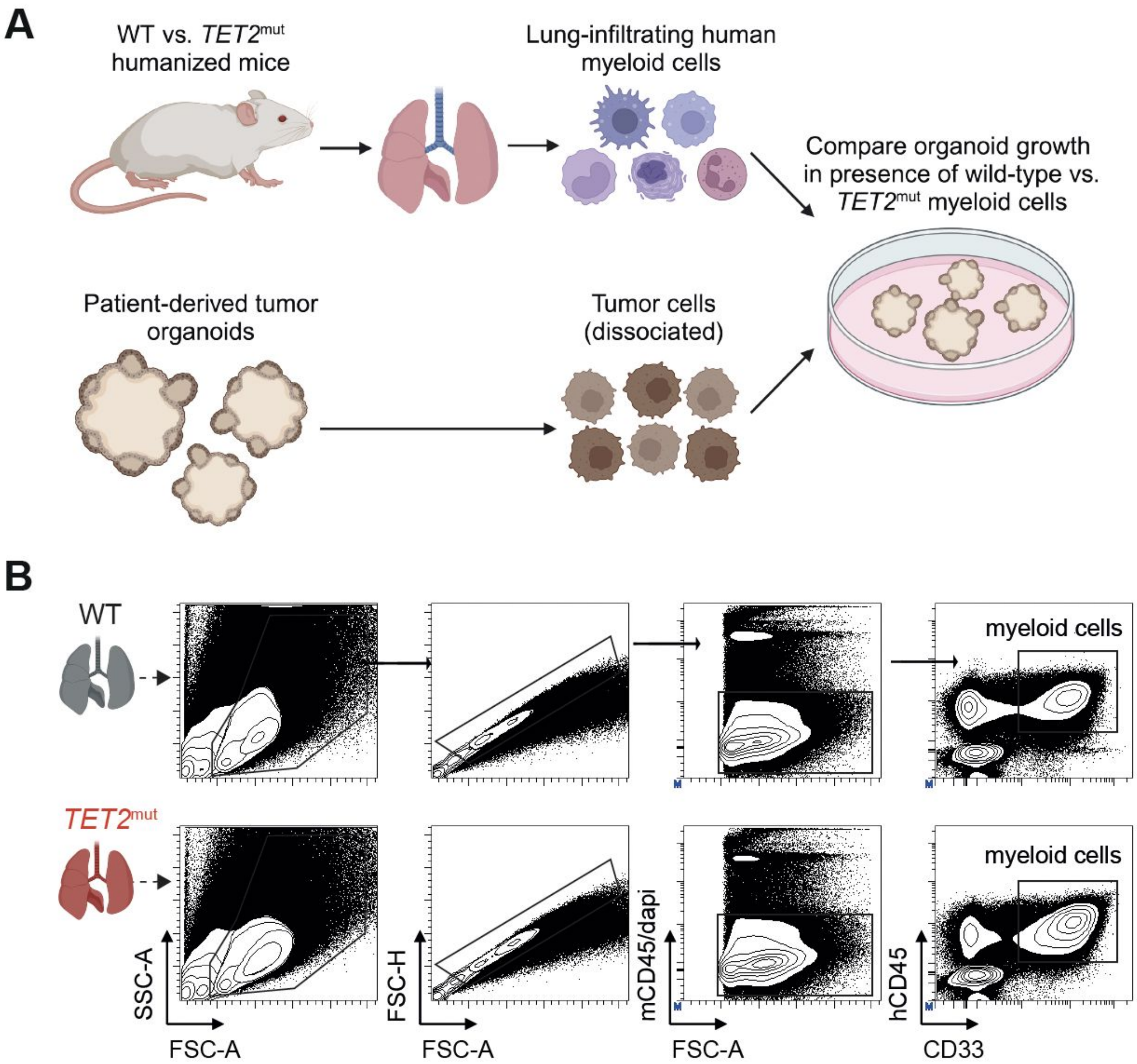

3. Supplementary Tables

3.1. Table S1

List of myeloid driver genes and consensus rules for pathogenicity of CHIP mutations.

Gene	Rules for Putative Pathogenecity	Accession Number
ARID2	Frameshift/nonsense/splice-site	ENST00000334344
ASXL1	Frameshift/nonsense/splice-site in exon 12-13	ENST00000375687
ASXL2	Frameshift/nonsense/splice-site in exon 12-13	ENST00000435504
ATM	Frameshift/nonsense/splice-site, Nonsynonymous AA 337, 2832, 2875, 2888, 3008	ENST00000278616
BCOR	Frameshift/nonsense/splice-site	ENST00000378444
BCORL1	Frameshift/nonsense/splice-site	ENST00000378444
BRAF	Nonsynonymous AA 458-712	ENST00000288602
BRCC3	Frameshift/nonsense/splice-site	ENST00000369462
CALR	Frameshift (C-terminal) AA 333-417	ENST00000316448
CBL	Frameshift/nonsense/splice-site, Nonsynonymous in Linker/RING finger domains AA 345-434	ENST00000264033
CEBPA	Frameshift/nonsense/splice-site	ENST00000498907
CHEK2	Frameshift/nonsense/splice-site	ENST00000382580
CREBBP	Frameshift/nonsense/splice-site, Nonsynonymous AA 1344-1639	ENST00000262367
CSF1R	Nonsynonymous AA 301, 969	ENST00000286301
CSF3R	Nonsynonymous AA 615, 618, Frameshift/nonsense/splice_site AA 618-863	ENST00000373103
CTCF	Frameshift/nonsense/splice-site	ENST00000264010
CUX1	Frameshift/nonsense/splice-site	ENST00000360264
DNMT3A	Frameshift/nonsense/splice-site, Nonsynonymous AA 290-912	ENST00000264709
DNMT3B	Frameshift/nonsense/splice-site	ENST00000328111
EED	Frameshift/nonsense/splice-site, Nonsynonymous AA 240, 363	ENST00000263360
EP300	Frameshift/nonsense/splice-site	ENST00000263253
ETNK1	Nonsynonymous AA 243-245	ENST00000266517
ETV6	Frameshift/nonsense/splice-site	ENST00000396373
EZH2	Frameshift/nonsense/splice-site, Nonsynonymous AA 500-751	ENST00000320356
FLT3	Nonsynonymous AA 800-850, 569-700	ENST00000241453
FOXP1	Frameshift/nonsense/splice-site	ENST00000491238
GATA2	Frameshift/nonsense/splice-site, Nonsynonymous AA 250-398	ENST00000341105
GNAS	Nonsynonymous AA 201, 227, 374, 844	ENST00000371100
GNB1	Nonsynonymous AA 57, 76, 77, 78, 80, 81, 88, 89	ENST00000378609
IDH1	Nonsynonymous AA 132, 178	ENST00000415913
IDH2	Nonsynonymous AA 140, 172	ENST00000330062
IKZF1	Frameshift/nonsense/splice-site	ENST00000439701
JAK2	Nonsynonymous AA 505-547, 617, 682, 683	ENST00000381652
KDM6A	Frameshift/nonsense/splice-site	ENST00000377967
KIT	Nonsynonymous AA 502-503, 540-600, 810-830	ENST00000288135
KMT2A	Frameshift/nonsense/splice-site	ENST00000534358
KMT2C	Frameshift/nonsense/splice-site	ENST00000262189
KMT2D	Frameshift/nonsense/splice-site, Nonsynonymous AA 5000-5537	ENST00000301067
KRAS	Nonsynonymous AA 12-14, 33, 58-64, 117, 146	ENST00000256078
LUC7L2	Frameshift/nonsense/splice-site	ENST00000541515

MGA	Frameshift/nonsense/splice-site	ENST00000219905
MPL	Nonsynonymous AA 500-520, 591, 592	ENST00000372470
MYD88	Nonsynonymous AA 217, 219, 240, 251, 265, 266, 273	ENST00000417037
NF1	Frameshift/nonsense/splice-site	ENST00000358273
NOTCH1	Frameshift/nonsense/splice-site at C-terminal end	ENST00000277541
NOTCH2	Frameshift/nonsense/splice-site at C-terminal end	ENST00000256646
NPM1	Frameshift AA 260-300	ENST00000296930
NRAS	Nonsynonymous AA 12-14, 60-64, 74, 146	ENST00000369535
PDS5B	Frameshift/nonsense/splice-site	ENST00000315596
PHF6	Frameshift/nonsense/splice-site, Nonsynonymous AA 42-132, 235-330	ENST00000332070
PHIP	Frameshift/nonsense/splice-site	ENST00000275034
PIGA	Frameshift/nonsense/splice-site	ENST00000333590
PPM1D	Frameshift/nonsense/splice-site in exon 5 or 6	ENST00000305921
PRPF40B	Frameshift/nonsense/splice-site	ENST00000548825
PRPF8	Nonsynonymous AA 1591, 1594, 1598	ENST00000572621
PTEN	Frameshift/nonsense/splice-site	ENST00000371953
PTPN11	Nonsynonymous AA 40-81, 112-197, 265-593	ENST00000351677
RAD21	Frameshift/nonsense/splice-site	ENST00000297338
RUNX1	Frameshift/nonsense/splice-site, Nonsynonymous AA 73-230	ENST00000300305
SETBP1	Nonsynonymous AA 855-880	ENST00000282030
SETD2	Frameshift/nonsense/splice-site	ENST00000409792
SF1	Frameshift/nonsense/splice-site	ENST00000377387
SF3A1	Frameshift/nonsense/splice-site	ENST00000215793
SF3B1	Nonsynonymous AA 500-1000	ENST00000335508
SH2B3	Frameshift/nonsense/splice-site, Nonsynonymous AA 364-459	ENST00000341259
SMC1A	Frameshift/nonsense/splice-site	ENST00000322213
SMC3	Frameshift/nonsense/splice-site	ENST00000361804
SRSF2	Nonsynonymous AA 57, 85-100, 107	ENST00000392485
STAG2	Frameshift/nonsense/splice-site	ENST00000218089
STAT3	Nonsynonymous in SH2 domain AA 584-674	ENST00000264657
SUZ12	Frameshift/nonsense/splice-site	ENST00000322652
TET2	Frameshift/nonsense/splice-site, Nonsynonymous AA 1104-1481, 1843-2002	ENST00000540549
TP53	Frameshift/nonsense/splice-site, Nonsynonymous, Synonymous T125T	ENST00000269305
U2AF1	Nonsynonymous AA 34-35, 156-157	ENST00000291552
U2AF2	Nonsynonymous AA 18, 143, 144, 187, 190	ENST00000308924
WT1	Frameshift/nonsense/splice-site	ENST00000332351
ZRSR2	Frameshift/nonsense/splice-site	ENST00000307771

3.2. Table S2

Baseline clinical parameters, CHIP mutations, and blood counts over time for patient CRUK1214.

		Surgery<br>T0	Follow-up<br>T0 + 127 days	Follow-up<br>T0 + 153 days	Follow-up<br>T0 + 175 days	Follow-up<br>T0 + 177 days	Follow-up<br>T0 + 181 days	Follow-up<br>T0 + 182 days	Follow-up<br>T0 + 183 days	Follow-up<br>T0 + 184 days	Follow-up<br>T0 + 185 days	Death<br>T0 + 200 days
**Baseline parameters**		Male										
		76 years old										
		Former smoker										
		Lung Adenocarcinoma										
		Stage IIB										
		No adjuvant treatment										
**CHIP mutations**	*TET2* R550*	Blood VAF 43%										
		Tumor VAF 26%										
	SRSF2 P95L	Blood VAF 43%										
		Tumor VAF 20%										
**Blood counts**		Hb	108	118	120	130	115	114	115	127	124	
		WBC	7.7	7.3	6.3	9.4	7.3	6.7	8.1	7.8	7.6	
		Plt	517	448	433	533	383	370	377	385	393	
		Neutrophils	6.2	5.5	4.7	7.3	5.4	5.3	6.6	6.2	6	
		Lymphocytes	0.8	0.9	0.9	1.1	0.8	0.7	0.7	0.7	0.8	
		Monoyctes	0.7	0.8	0.6	0.9	0.9	0.7	0.7	0.8	0.7	

Hb Hemoglobin in g/L
WBC white blood cells x10^9/L
Platelets x10^9/L
Neutrophils x10^9/L
Lymphocytes x10^9/L
Monocytes x10^9/L

3.3. Table S3

TRACERx characteristics across the categories of patients (i) without CHIP; (ii) with CHIP in absence of TI-CH (blood-only CHIP); and (iii) with TI-CH.

	TRACERx Primary Stage I-III NSCLC (n=421)		
	**No CHIP**	**blood-only CHIP**	**TI-CH**
**N (%)**	278 (66%)	83 (20%)	60 (14%)
**Age in years, median (IQR)**	68 (62-74)	71 (65-77)	74 (68-79)
**Sex, No. (%)**			
Female	130 (47%)	39 (47%)	19 (32%)
Male	148 (53%)	44 (53%)	41 (68%)
**Smoking status, No. (%)**			
Current smoker	125 (45%)	34 (41%)	21 (35%)
Former smoker	130 (47%)	44 (53%)	37 (62%)
Never smoker	23 (8%)	5 (6%)	2 (3%)
**Race, No (%)**			
White	267 (96%)	79 (96%)	58 (97%)
Asian	2 (1%)	0 (0%)	0 (0%)
Black	4 (1%)	2 (2%)	1 (2%)
Other/Missing	5 (2%)	2 (2%)	1 (2%)
**Histology, No (%)**			
LUAD	160 (58%)	53 (64%)	27 (45%)
LUSC	84 (30%)	25 (30%)	25 (42%)
Other	34 (12%)	5 (6%)	8 (13%)
**Main oncogenic driver, No (%)**			
TP53 mutation	176 (63%)	60 (72%)	43 (72%)
KRAS mutation	87 (31%)	25 (30%)	15 (25%)
STK11 mutation	37 (13%)	15 (18%)	11 (18%)
EGFR mutation	22 (8%)	5 (6%)	2 (3%)
Oncogenic isoforms$	15 (5%)	3 (4%)	1 (2%)

$: *RET*, *ROS1*, *ALK* and *MET* oncogenic isoforms

IQR: interquartile range; No: number; CI: confidence interval.

3.4. Table S4

Main cancer types in the MSK-IMPACT cohort. Only cancer types with 20 or more patients with primary or metastatic tumors included in the study are shown.

Cancer Type (primary tumor diagnosis)	N. of patients with primary tumor analyzed	N. of patients with a metastatic tumor (any site) analyzed
Non-Small Cell Lung Cancer	3770	2139
Colorectal Cancer	3729	1149
Breast Cancer	2898	2725
Endometrial Cancer	2220	572
Prostate Cancer	2021	1044
Glioma	1946	65
Pancreatic Cancer	1922	1136
Bladder Cancer	1712	368
Esophagogastric Cancer	1460	416
Soft Tissue Sarcoma	1383	685
Hepatobiliary Cancer	1063	401
Ovarian Cancer	994	1158
Renal Cell Carcinoma	804	375
Melanoma	622	1189
Germ Cell Tumor	396	299
Thyroid Cancer	359	400
Mesothelioma	336	58
Gastrointestinal Stromal Tumor	318	116
Skin Cancer, Non-Melanoma	288	160
Head and Neck Cancer	280	305
Bone Cancer	251	158
Cervical Cancer	243	111
Small Cell Lung Cancer	198	238
Peripheral Nervous System	198	104
Cancer of Unknown Primary	166	1374
Uterine Sarcoma	165	167
Appendiceal Cancer	154	176
CNS Cancer	134	14
Salivary Gland Cancer	122	186
Small Bowel Cancer	121	39
Ampullary Cancer	107	23
Gastrointestinal Neuroendocrine Tumor	105	107
Nerve Sheath Tumor	99	23
Anal Cancer	99	52
Sellar Tumor	98	6
Retinoblastoma	94	2
Adrenocortical Carcinoma	66	34
Sex Cord Stromal Tumor	54	41
Breast Sarcoma	52	13
Thymic Tumor	48	28
Wilms Tumor	46	13
Vaginal Cancer	44	15
Mature B-Cell Neoplasms	39	5
Miscellaneous Brain Tumor	36	3
Embryonal Tumor	31	11
Tubular Adenoma of the Colon	26	0
Histiocytosis	25	5
Miscellaneous Neuroepithelial Tumor	23	10

4. Supplementary References

1. Frankell AM, Dietzen M, Al Bakir M, et al. The evolution of lung cancer and impact of subclonal selection in TRACERx. Nature 2023;616(7957):525–33.

2. Enfield KSS, Colliver E, Lee C, et al. Spatial Architecture of Myeloid and T Cells Orchestrates Immune Evasion and Clinical Outcome in Lung Cancer. Cancer Discov 2024;14(6):1018–47.

3. Cheng DT, Mitchell TN, Zehir A, et al. Memorial Sloan Kettering-Integrated Mutation Profiling of Actionable Cancer Targets (MSK-IMPACT): A Hybridization Capture-Based Next-Generation Sequencing Clinical Assay for Solid Tumor Molecular Oncology. J Mol Diagn 2015;17(3):251–64.

4. Kundra R, Zhang H, Sheridan R, et al. OncoTree: A Cancer Classification System for Precision Oncology. JCO Clin Cancer Inform 2021;5:221–30.

5. Zehir A, Benayed R, Shah RH, et al. Mutational landscape of metastatic cancer revealed from prospective clinical sequencing of 10,000 patients. Nat Med 2017;23(6):703–13.

6. Nguyen B, Fong C, Luthra A, et al. Genomic characterization of metastatic patterns from prospective clinical sequencing of 25,000 patients. Cell 2022;185(3):563–75.e11.

7. Bolton KL, Ptashkin RN, Gao T, et al. Cancer therapy shapes the fitness landscape of clonal hematopoiesis. Nat Genet 2020;52(11):1219–26.

8. Stonestrom AJ, Menghrajani KN, Devlin SM, et al. High-risk and silent clonal hematopoietic genotypes in patients with nonhematologic cancer. Blood Adv 2024;8(4):846–56.

9. Niroula A, Sekar A, Murakami MA, et al. Distinction of lymphoid and myeloid clonal hematopoiesis. Nat Med 2021;27(11):1921–7.

10. Kar SP, Quiros PM, Gu M, et al. Genome-wide analyses of 200,453 individuals yield new insights into the causes and consequences of clonal hematopoiesis. Nat Genet 2022;54(8):1155–66.

11. Pich O, Reyes-Salazar I, Gonzalez-Perez A, Lopez-Bigas N. Discovering the drivers of clonal hematopoiesis. Nat Commun 2022;13(1):4267.

12. Vlasschaert C, Mack T, Heimlich JB, et al. A practical approach to curate clonal hematopoiesis of indeterminate potential in human genetic data sets. Blood 2023;141(18):2214–23.

13. Satas G, Zaccaria S, El-Kebir M, Raphael BJ. DeCiFering the elusive cancer cell fraction in tumor heterogeneity and evolution. Cell Syst 2021;12(10):1004–18.e10.

14. Tarabichi M, Salcedo A, Deshwar AG, et al. A practical guide to cancer subclonal reconstruction from DNA sequencing. Nat Methods 2021;18(2):144–55.

15. Álvarez-Prado ÁF, Maas RR, Soukup K, et al. Immunogenomic analysis of human brain metastases reveals diverse immune landscapes across genetically distinct tumors. Cell Rep Med 2023;4(1):100900.

16. Van der Auwera GA, Carneiro MO, Hartl C, et al. From FastQ data to high confidence variant calls: the Genome Analysis Toolkit best practices pipeline. Curr Protoc Bioinformatics 2013;43(1110):11.10.1–11.10.33.

17. Rodriguez-Meira A, O'Sullivan J, Rahman H, Mead AJ. TARGET-Seq: A Protocol for High-Sensitivity Single-Cell Mutational Analysis and Parallel RNA Sequencing. STAR Protoc 2020;1(3):100125.

18. Aegerter H, Kulikauskaite J, Crotta S, et al. Influenza-induced monocyte-derived alveolar macrophages confer prolonged antibacterial protection. Nat Immunol 2020;21(2):145–57.

19. Molina-Arcas M, Moore C, Rana S, et al. Development of combination therapies to maximize the impact of KRAS-G12C inhibitors in lung cancer. Sci Transl Med 2019;11(510).

20. Mugarza E, van Maldegem F, Boumelha J, et al. Therapeutic KRAS inhibition drives effective interferon-mediated antitumor immunity in immunogenic lung cancers. Sci Adv 2022;8(29):eabm8780.

21. Hynds RE, Huebner A, Pearce DR, et al. Representation of genomic intratumor heterogeneity in multi-region non-small cell lung cancer patient-derived xenograft models. Nat Commun 2024;15(1):4653.

22. Cattaneo CM, Dijkstra KK, Fanchi LF, et al. Tumor organoid-T-cell coculture systems. Nat Protoc 2020;15(1):15–39.

23. Kim M, Mun H, Sung CO, et al. Patient-derived lung cancer organoids as in vitro cancer models for therapeutic screening. Nat Commun 2019;10(1):3991.

24. Dijkstra KK, Monkhorst K, Schipper LJ, et al. Challenges in Establishing Pure Lung Cancer Organoids Limit Their Utility for Personalized Medicine. Cell Rep 2020;31(5):107588.

25. Huerga Encabo H, Aramburu IV, Garcia-Albornoz M, et al. Loss of TET2 in human hematopoietic stem cells alters the development and function of neutrophils. Cell Stem Cell 2023;30(6):781–99.e9.

26. Huerga Encabo H, Ulferts R, Sharma A, Beale R, Bonnet D. Infecting human hematopoietic stem and progenitor cells with SARS-CoV-2. STAR Protoc

2021;2(4):100903.

27. Schindelin J, Arganda-Carreras I, Frise E, et al. Fiji: an open-source platform for biological-image analysis. Nat Methods 2012;9(7):676–82.
